# Left ventricular-arterial coupling parameters predict incident cardiovascular events and mortality in UK Biobank

**DOI:** 10.1016/j.jocmr.2026.102709

**Published:** 2026-03-06

**Authors:** Chaoyang Lin, Yixin Yang, Qianyao Lai, Jiahui Chen, Enhao Wei, Shuyun Wu, Lijun Luo, Maosen Lin, Feng Hu, Lin Fan, Enhui Yao

**Affiliations:** a Department of Cardiology, Fujian Medical University Union Hospital, Fujian Cardiovascular Medical Center, Fujian Institute of Coronary Artery Disease, Fujian Cardiovascular Research Center, Fuzhou, PR China; b School of Health, Fujian Medical University, Fuzhou, PR China; c Department of Colorectal Surgery, Clinical Oncology School of Fujian Medical University, Fuzhou, PR China

**Keywords:** Ventricular-arterial coupling, Cardiac magnetic resonance, Incident cardiovascular events, Cardiovascular mortality, Survival analysis

## Abstract

**Background:**

Ventricular-arterial coupling (VAC) is fundamental to cardiovascular efficiency, but its value for predicting cardiovascular disease (CVD) in the general population is unclear. This study aimed to evaluate the predictive value of three novel non-invasive VAC parameters—the ratio of arterial stiffness index to global longitudinal strain (ASI/GLS), estimated pulse wave velocity to GLS (ePWV/GLS), and left ventricular end-systolic volume to stroke volume (LVESV/LVSV)—for incident atrial fibrillation (AF), stroke, heart failure (HF), coronary heart disease (CHD), all-cause and CVD mortality.

**Methods:**

We analyzed UK Biobank participants free of baseline significant structural CVD or prior major cardiac surgery. VAC parameters were derived from arterial stiffness metrics and cardiac magnetic resonance (CMR). Associations were assessed using multivariable Cox or Fine-Gray models with false discovery rate (FDR) correction. Incremental prognostic value was evaluated using likelihood ratio tests, C-indices, and continuous net reclassification improvement (NRI).

**Results:**

The study included 38,144 participants (mean age 63.5 ± 7.5 years; 47.2% men) with a median follow-up of 4.82 years. After adjustment for clinical features, all three VAC parameters were associated with increased risk of AF, stroke, HF, CHD, all-cause and CVD mortality (except ASI/GLS for CHD). For incident HF, the addition of VAC parameters yielded substantial incremental value, raising C-indices to 0.789–0.835 with NRIs of 11.0%–24.1%. Beyond some respective conventional CMR indices, all three VAC parameters remained independent predictors for incident stroke, whereas ePWV/GLS was specifically independent for incident AF (hazard ratio [HR] 1.19, 95% confidence interval [CI] 1.10–1.29), HF (HR 1.26, 95% CI 1.11–1.43), and CHD (HR 1.11, 95% CI 1.001–1.23). Furthermore, while VAC parameters were independently associated with all-cause mortality regardless of LVEF, their associations with CVD mortality were largely attenuated by LVEF.

**Conclusion:**

In the general population, non-invasive VAC parameters predict various adverse cardiovascular outcomes. Incorporating ASI/GLS or ePWV/GLS into traditional risk assessment enhances prognostic value in specific clinical scenarios.

## Graphical abstract

**Figure:**
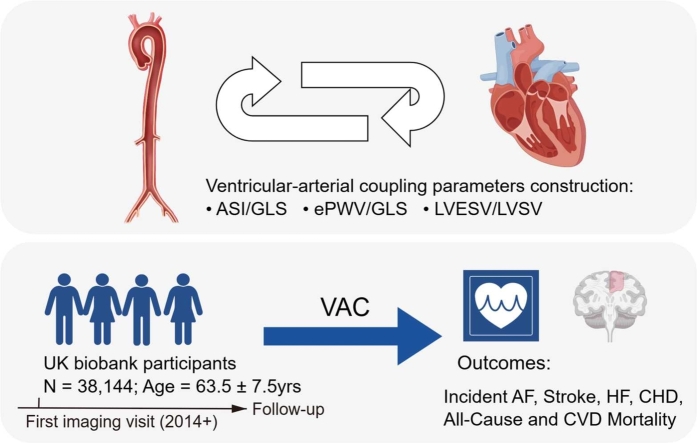
VAC parameters predict incident cardiovascular events and mortality. *AF* atrial fibrillation, *ASI* arterial stiffness index, *CHD* coronary heart disease, *CVD* cardiovascular disease, *ePWV* estimated pulse wave velocity, *GLS* left ventricular global longitudinal strain, *HF* heart failure, *LVESV* left ventricular end-systolic volume, *LVSV* left ventricular stroke volume, *VAC* ventricular-arterial coupling

## 1 Introduction

Ventricular-arterial coupling (VAC) is a fundamental physiological concept describing the dynamic interaction between the heart's pumping function and the properties of the arterial system. By governing the efficiency of energy transfer, this mechanism ensures optimal organ perfusion and defines the overall performance of the cardiovascular system 1, 2. The adaptation of VAC to balance ventricular end-systolic and arterial elastance originates from invasive pressure-volume (P-V) relationship analysis in cardiac function assessment using cardiac catheterization [3]. With aging, synchronized stiffening of the arterial system and left ventricle (LV) maintains resting VAC and ensures energetic efficiency. However, this adaptation is characterized by enhanced load-sensitivity and diminished functional reserve, a change that may foreshadow the transition from physiological aging to pathological conditions such as heart failure (HF) [4].

The prognostic relevance of VAC spans diverse cardiovascular disease (CVD), holding potential to guide clinical decision-making [1]. While various non-invasive methods have evolved to quantify it—from the classic ratio of LV effective arterial elastance to end-systolic elastance (Ea/Ees) to novel integrative indices such as the ratio of pulse wave velocity to LV global longitudinal strain (PWV/GLS)—their utility has been predominantly demonstrated in patients with established disease, particularly hypertension and HF [1]. Although emerging evidence suggests these VAC markers could offer incremental value beyond traditional risk factors 1, 5, their ability to predict new-onset CVD and mortality in the general population remains largely unexplored.

In the context of the UK Biobank, where the reference standard carotid-femoral pulse wave velocity (cfPWV) is unavailable, the arterial stiffness index (ASI) 6, 7, 8 and estimated PWV (ePWV) 9, 10 serve as practical surrogates. Both metrics have demonstrated significant correlations with cfPWV and independent prognostic capacity for CVD. We also derived cardiovascular magnetic resonance (CMR) metrics, including GLS. Therefore, the objective of this study was to assess the predictive value of three non-invasive VAC parameters (ASI/GLS, ePWV/GLS, and the ratio of LV end-systolic volume to stroke volume [LVESV/LVSV]) for incident atrial fibrillation (AF), stroke, HF, coronary heart disease (CHD), all-cause, and CVD mortality in this large general population cohort. Furthermore, we sought to determine whether these integrated parameters yield additional predictive value over existing evidence-based CMR indices for each respective outcome.

## 2 Methods

### 2.1 Study population and study design

**Fig. 1:**
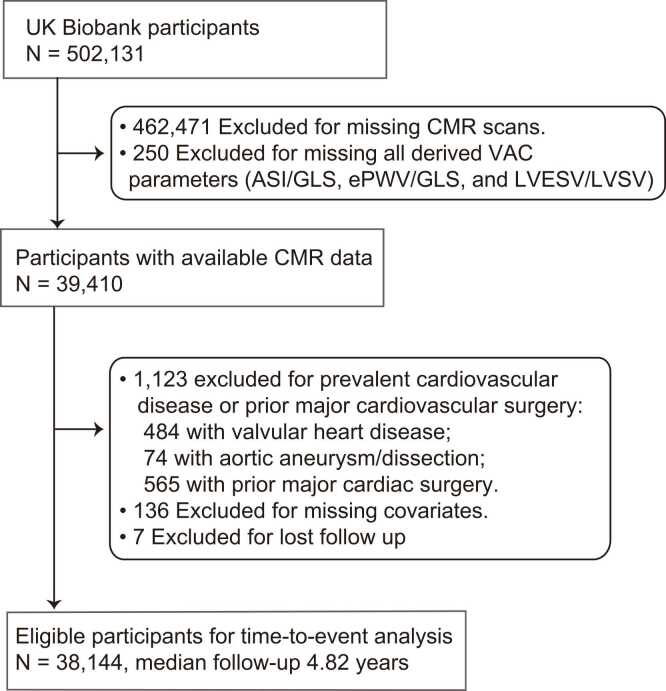
Flowchart of the study. This flowchart illustrates the selection process for eligible participants from the UK Biobank cohort. Major cardiac surgery was defined as structural, congenital, valvular, aortic surgery, and coronary revascularization. *ASI* arterial stiffness index, *CMR* cardiovascular magnetic resonance, *ePWV* estimated pulse wave velocity, *GLS* left ventricular global longitudinal strain, *LVESV* left ventricular end-systolic volume, *LVSV* left ventricular stroke volume, *VAC* ventricular-arterial coupling.

This study adhered to the Strengthening the Reporting of Observational studies in Epidemiology (STROBE) cohort guidelines (Supplementary Table S1). Between 2006 and 2010, the UK Biobank recruited more than 500,000 volunteers aged 37 to 73. Starting in 2014, the UK Biobank initiated a multi-modal imaging enhancement, and by early 2020, nearly 50,000 participants had undergone an imaging assessment that included CMR [11]. Our study cohort was selected from individuals who attended the first imaging visit and had available CMR data. From this group, we excluded participants with prevalent valvular heart disease, aortic aneurysm, and dissection at baseline. We also excluded those with a history of major cardiac surgery, defined as structural, congenital, valvular, aortic surgery, and coronary revascularization (Supplementary Table S2). These exclusion criteria were applied because such pre-existing conditions or interventions may alter cardiac and vascular hemodynamics, thereby compromising the accuracy of VAC assessment [1]. After further excluding participants with missing covariate data and those lost to follow-up, the final cohort included 38,144 individuals (Fig. 1).

### 2.2 Ethical review

This study adhered to the Declaration of Helsinki. The UK Biobank project received ethical approval from the NHS National Research Ethics Service, initially on June 17, 2011 (Reference 11/NW/0382), with an extension granted on June 18, 2021 (Reference 21/NW/0157). Written informed consent was obtained from all participants, and none of the subjects included in this analysis had withdrawn their consent.

### 2.3 Ascertainment of ASI

Pulse wave ASI was assessed via a rapid, non-invasive, and cost-effective technique that requires no specialized expertise and minimizes operator dependency (Data-Field 21021) [12]. During the initial imaging visit, ASI was evaluated with the PulseTrace PCA2 device (CareFusion, San Diego, California), which captured arterial pulse waveforms through finger-based photoplethysmography using an infrared sensor clipped to the end of the index finger. The reading was taken over 10–15 s. The measurement was repeated on a larger finger or thumb if the waveform was not fully visible or failed to stabilize within a minute after sensor attachment. The waveform morphology reflected the transit time of pulse waves traveling through the lower body’s arterial system and returning as reflected waves [13]. Notably, validation studies have established ASI as a robust surrogate for arterial stiffness, demonstrating both a significant correlation with cfPWV 7, 8 and independent predictive value for cardiovascular events, mortality, and 10-year atherosclerotic cardiovascular disease (ASCVD) risk 6, 14.

### 2.4 Cardiac magnetic resonance

CMR imaging was performed on 1.5-T MAGNETOM Aera scanners (Syngo Platform VD13A, Siemens Healthineers, Erlangen, Germany) following a standardized protocol detailed previously [15]. The protocol included the acquisition of balanced steady-state free precession (bSSFP) cine images in both long- and short-axis views. All volumetric, functional, and strain phenotypes were obtained directly as a derived data field from the UK Biobank (Category 157), generated using a machine learning-based automated pipeline detailed by Bai et al. [16]. This pipeline utilized a fully convolutional network, trained on manual annotations from 3975 participants, for cardiac segmentation, and employed non-rigid image registration for motion tracking. The generation process incorporated a rigorous quality control protocol where all segmentations were first manually reviewed by trained CMR experts, and a subsequent automated check excluded participants with failed motion tracking or physiologically implausible strain values. For the construction of VAC, the principal indices extracted from this quality-controlled dataset were LVESV, LVSV, and GLS, which was derived from motion tracking results on the long-axis four-chamber view. Additionally, we extracted LV indices including LV ejection fraction (LVEF), LV end-diastolic volume (LVEDV), and LV mass (LVM); and left atrial (LA) metrics including LA ejection fraction (LAEF), maximal LA volume (LAVmax), and minimal LA volume (LAVmin), to facilitate correlation analysis and comparative prognostic modeling.

### 2.5 Construction of VAC parameters

Historically, VAC has been conceptualized as the Ea/Ees ratio, for which the gold-standard measurement relies on invasive P-V loop analysis, thus limiting its routine clinical application 17, 18. While P-V loops excel at characterizing global chamber hemodynamics, they may not directly reflect intrinsic myocardial tissue deformation [18]. Accordingly, recent perspectives emphasize that an approach that couples the respective gold-standard non-invasive markers for arterial load (via PWV) and LV contractility (via GLS) would be more appropriate for characterizing VAC in many settings 1, 19. Given the unavailability of cfPWV, our study utilized two validated surrogates for arterial stiffness. First, we employed the ASI, which has demonstrated high correlation with cfPWV and independent prognostic capacity for CVD and mortality 6, 7, 8. Second, we utilized the ePWV, calculated from age and mean blood pressure (MBP), which has demonstrated robust correlations with cfPWV and independent predictive capacity 9, 10.

Based on this framework, we constructed novel VAC parameters of interest: the ASI/GLS and ePWV/GLS ratios, which pair surrogates of arterial stiffness (ASI and ePWV, respectively) with CMR-derived GLS. The ePWV was calculated using the following formula: $\mathrm{ePWV}=\;9.587-0.402\times \mathrm{age}+4.560\times 10^{-3}\times {\mathrm{age}}^{2}-2.621\times 10^{-5}\times {\mathrm{age}}^{2}\times \mathrm{MBP}+3.176\times 10^{-3}\times \mathrm{age}\times \mathrm{MBP}-1.832\times 10^{-2}\times \mathrm{MBP}.$ MBP was calculated as diastolic blood pressure + 0.4 × (systolic blood pressure - diastolic blood pressure). Regarding the traditional Ea/Ees ratio, although the benchmark single-beat estimation method developed by Chen et al. is considered the non-invasive reference method 5, 20, it was not feasible for the UK Biobank cohort. This method requires the calculation of a normalized time variable, tNd, which is defined as the ratio of the pre-ejection period to the total systolic period and, in turn, necessitates precise valvular event timings [5]. These timings are typically derived from high-temporal-resolution modalities like aortic Doppler ultrasonography, which were not part of the standardized UK Biobank CMR imaging protocol [21]. To address this limitation, we utilized the LVESV/LVSV ratio as a surrogate. By approximating Ea as LV end-systolic pressure over stroke volume (LVESP/LVSV) and Ees as LV end-systolic pressure over end-systolic volume (LVESP/LVESV), the Ea/Ees ratio mathematically simplifies to LVESV/LVSV. This metric, despite its simplicity, demonstrates comparable correlation with invasively derived VAC 5, 19.

### 2.6 Ascertainment of covariates

All baseline covariates were ascertained exclusively from the participant's first imaging visit via touchscreen questionnaires, physical measurements, and verbal interviews. Socio-demographic factors include age, sex, and race (white vs non-white). Anthropometric and lifestyle factors include body mass index (BMI), smoking status (never, previous, or current), alcohol intake frequency (never, monthly to weekly, or daily), and healthy physical activity. Healthy physical activity was defined as meeting the criteria of ≥150 min of moderate or ≥75 min of vigorous activity per week, or an equivalent combination [22]. Clinical and family history covariates include a family history of heart disease (in parents) and prevalent comorbidities such as hypertension, dyslipidemia, diabetes, and CHD. These comorbidities were defined using standardized diagnostic codes from the International Classification of Diseases (ICD-9 and ICD-10), with detailed definitions provided in Supplementary Table S3.

### 2.7 Clinical outcomes of interest

The outcomes of interest for this study were incident AF, stroke, HF, CHD, all-cause mortality, and total CVD mortality (ICD-10 codes I00–I99). These outcomes were ascertained through self-reported diagnoses (utilized to identify prevalent cases at baseline), hospital episode records, and death registry data, with detailed diagnostic codes provided in Supplementary Table S4. For incident analyses, participants with a prevalent diagnosis of the specific endpoint prior to the first imaging visit were excluded. Follow-up duration was calculated from the date of the first imaging assessment until the first occurrence of the specific outcome event, death, or the censoring date (December 19, 2022).

### 2.8 Statistical analysis

Baseline characteristics for continuous variables are expressed as mean ± standard deviation (SD) or median (interquartile range, IQR), depending on distribution skewness, and for categorical and discrete variables as counts and percentages. Analysis was conducted separately for each outcome (AF, stroke, HF, CHD, all-cause, and CVD mortality) and for each VAC parameter (ASI/GLS, ePWV/GLS, and LVESV/LVSV). Quality control was performed by removing statistical outliers for the individual VAC components and the VAC ratios, if their values were four times the IQR (4 × IQR) below the first quartile or above the third quartile [23]. Associations between VAC and incident outcomes were assessed using multivariable Cox proportional hazards regression to derive hazard ratios (HRs) with 95% confidence intervals (CIs). For CVD mortality, Fine-Gray subdistribution hazard models were used to account for non-CVD death as a competing risk. Additionally, cause-specific Cox models were constructed to evaluate the associations of VAC with CVD and non-CVD mortality. Multicollinearity was assessed using the variance inflation factor (VIF), and the proportional hazards assumption was checked.

We constructed several models to assess the associations. First, models were adjusted for age and sex only. Second, we further adjusted for clinical features, including race, education, BMI, smoking status, alcohol intake frequency, healthy physical activity, family history of heart disease, prevalent hypertension, dyslipidemia, diabetes, and CHD (excluded in analyses predicting incident CHD). Subsequently, to evaluate whether VAC provides incremental value beyond existing imaging markers, we additionally adjusted for outcome-specific CMR metrics **(**with volumes and mass indexed to body surface area**)** based on previous literature. Specifically, we adjusted for LAVmax-I 24, 25, 26 and LAEF 24, 26 for AF, while LAVmax-I 25, 27 for stroke. For incident HF, the models included LV global function index (LVGFI) 28, 29, LVEDVI [30], and LVEF [31]. While for CHD, we included LVESVI [32], LVEDVI 30, 33, and LVEF [31]. Lastly, LVMI 34, 35 and LVEF [31] were used for all-cause and CVD mortality. LVGFI (%) was calculated as [LVSV/(LVEDV+LVESV/2+LVM/1.05)]×100 [28].

All models used a complete-case only approach (see Supplemental Table S5 for sample sizes and detailed exclusion reasons). To make effect-sizes comparable, we standardized them to have a mean value of 0 and SD of 1 for regression analysis as a continuous dimension. The Benjamini-Hochberg false discovery rate (FDR) was applied for multiple testing correction. E-value was calculated to estimate the potential impact of unmeasured confounding. To assess the incremental value of VAC, we compared the predictive performance of models with and without VAC parameters. Model goodness-of-fit was investigated using likelihood ratio tests (LRT), reporting the χ^2^ statistic. We evaluated model discrimination using Harrell’s C-indices and compared these indices [36]. We also calculated the continuous net reclassification improvement (NRI). Notably, the LRT was considered sufficient to evaluate added predictive value, given the vastly inferior statistical properties of C-index comparisons in nested models 37, 38. Moreover, to determine whether VAC provides incremental information over its constituent components, comparisons of C-indices and LRT were performed in stepwise nested models conditional on clinical features [39]. To explore potential non-linear associations, VAC parameters were categorized into quartiles, with the quartile exhibiting the lowest risk serving as the reference in models adjusted for clinical features. Incidence rates were calculated per 1000 person-years. Univariable cumulative risk curves were generated and compared using the log-rank test or Gray's test, as appropriate. We fitted restricted cubic spline (RCS) regressions to investigate the dose-response relationship. The number of knots (3–7) of the RCS was selected based on the minimum Akaike Information Criterion (AIC) to balance model flexibility and parsimony. Subgroup analyses stratified by age, sex, BMI, hypertension, and dyslipidemia were performed to evaluate the consistency of associations. Finally, Pearson correlation coefficients were calculated to examine the relationships between arterial stiffness, CMR metrics, and VAC parameters. A two-sided *P*-value < 0.05 was considered statistically significant. FDR q-value < 0.05 denoted significance after multiple testing correction. All analyses were conducted using R statistical software (version 4.5.0).

## 3 Results

### 3.1 Participant characteristics

The baseline characteristics of the study population are shown in Table 1. The study population had a mean age of 63.5 ± 7.5 years, with 47.2% men, and was predominantly of white ethnicity (93.1%). Most participants were non-smokers or had quit (62.7% and 33.8%, respectively). In terms of clinical comorbidities, 38.3% had prevalent hypertension, 36.5% had dyslipidemia, 5.6% had diabetes, and 5.3% had CHD. The median (IQR) values of the VAC parameters were 0.51 (0.40–0.64) m**·**s^-1^**·**%^-1^ for ASI/GLS, 0.56 (0.49–0.65) m**·**s^-1^**·**%^-1^ for ePWV/GLS, and 0.67 (0.57–0.79) for LVESV/LVSV.

**Table 1:** Baseline characteristics of the study participants

Characteristics	N = 38,144
*Demographics and lifestyle*	
Age, years, mean± SD	63.5± 7.5
Sex (men), n (%)	18,007 (47.2%)
Race (white), n (%)	35,512 (93.1%)
Education (college or above), n (%)	18,487 (48.5%)
BMI, kg/m^2^, median (IQR)	25.9 (23.5, 28.8)
Smoking status, n (%)	
Never	23,904 (62.7%)
Previous	12,893 (33.8%)
Current	1347 (3.5%)
Alcohol intake frequency, n (%)	
Never	2501 (6.6%)
Month to week	29,204 (76.6%)
Daily	6439 (16.9%)
Healthy physical activity (yes), n (%)	24,573 (64.4%)
Family history of heart disease (yes), n (%)	19,299 (50.6%)
*Comorbidities*	
Hypertension (yes), n (%)	14,608 (38.3%)
Dyslipidemia (yes), n (%)	13,914 (36.5%)
Diabetes (yes), n (%)	2144 (5.6%)
Coronary heart disease (yes), n (%)	2010 (5.3%)
*Arterial stiffness & CMR metrics*	
ASI, m/s, median (IQR)	9.41 (7.44, 11.33)
ePWV, m/s, mean±SD	10.51± 1.64
BSA, m^2^, mean±SD	1.86± 0.21
GLS, %, mean±SD	−18.5± 2.8
LVEF, %, mean±SD	59.6± 6.1
LVEDV, mL, mean±SD	147.3± 33.5
LVESV, mL, median (IQR)	57.1 (46.6, 70.4)
LVSV, mL, mean±SD	87.2± 19.2
LVGFI, %, mean±SD	47.8± 6.8
LVM, g, mean±SD	85.6± 22.0
LAEF, %, mean±SD	61.3± 9.4
LAVmax, mL, median (IQR)	69.8 (56.7, 85.2)
LAVmin, mL, median (IQR)	27.0 (19.8, 35.4)
*VAC parameters*	
ASI/GLS, m**·**s^-1^**·**%^-1^, median (IQR)	0.51 (0.40, 0.64)
ePWV/GLS, m**·**s^-1^**·**%^-1^, median (IQR)	0.56 (0.49, 0.65)
LVESV/LVSV, median (IQR)	0.67 (0.57, 0.79)

Values are given as mean ± standard deviation, median (IQR), or number (percentage). *ASI* arterial stiffness index, *BMI* body mass index, *BSA* body surface area, *CMR* cardiac magnetic resonance, *ePWV* estimated pulse wave velocity, *GLS* left ventricular global longitudinal strain, *IQR* interquartile range, *LAEF* left atrial ejection fraction, *LAVmax* maximal left atrial volume, *LAVmin* minimal left atrial volume, *LVEDV* left ventricular end-diastolic volume, *LVEF* left ventricular ejection fraction, *LVESV* left ventricular end-systolic volume, *LVGFI* left ventricular global function index, *LVM* left ventricular mass, *LVSV* left ventricular stroke volume, *SD* standard deviation, *VAC* ventricular-arterial coupling

### 3.2 Association between VAC and cardiovascular morbidity and mortality

**Fig. 2:**
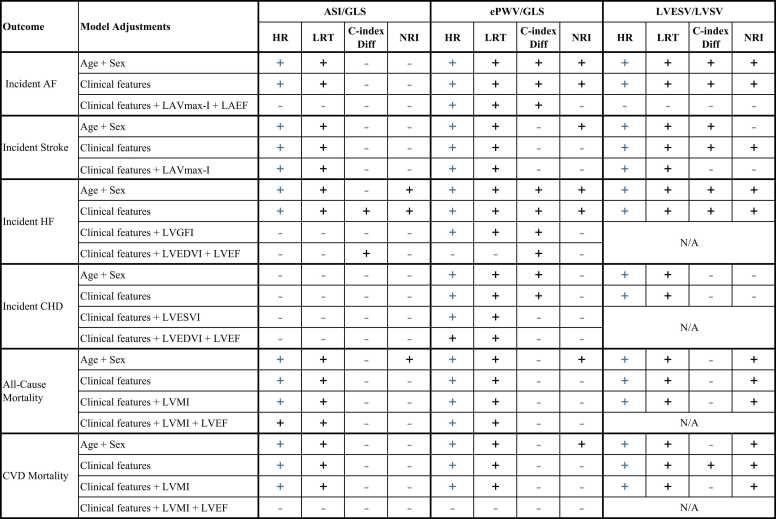
Summary of Findings: VAC parameters predict adverse outcomes. Symbols summarize the statistical significance of the association (HR), improvement in model discrimination (C-index Diff), and model fit (LRT).  denotes *P*-value ≥ 0.05; Black + denotes *P*-value < 0.05;  indicates statistical significance after multiple testing correction (FDR q-value < 0.05) for HR. Clinical features included age, sex, race, education, body mass index, smoking status, alcohol intake frequency, healthy physical activity, family history of heart disease, prevalent hypertension, dyslipidemia, diabetes, and prevalent CHD (excluded from models predicting incident CHD). *AF* atrial fibrillation, *ASI/GLS* ratio of arterial stiffness index to global longitudinal strain, *CHD* coronary heart disease, *CVD* cardiovascular disease, *Diff* difference, *ePWV/GLS* ratio of estimated pulse wave velocity to global longitudinal strain, *FDR* false discovery rate, *HF* heart failure, *HR* hazard ratio, *LAEF* left atrial ejection fraction, *LAVmax-I* maximum left atrial volume index, *LRT* likelihood ratio test, *LVEDVI* left ventricular end-diastolic volume index, *LVEF* left ventricular ejection fraction, *LVESVI* left ventricular end-systolic volume index, *LVESV/LVSV* ratio of left ventricular end-systolic volume to stroke volume, *LVGFI* left ventricular global function index, *LVMI* left ventricular mass index, *N/A* not applicable, *VAC* ventricular-arterial coupling

Over a median follow-up of 4.82 years, there were 727 incident cases of AF, 331 cases of stroke, 255 cases of HF, 600 cases of CHD, and 707 all-cause deaths, including 113 CVD deaths. Table 2 and Table 3 present the multivariable associations and the incremental value of VAC parameters for predicting these events. Fig. 2 briefly illustrates the summary findings.

**Table 2:** Multivariable association and incremental value of VAC parameters for predicting incident AF, Stroke, HF, and CHD

Multivariable Model	Association of VAC with outcome			Incremental predictive value of VAC		
	Case / Total	HR (95% CI)		LRT Δχ²	C-index (Change)	Total NRI
*Incident AF (Case = 727, Total = 37,154)*						
Age + Sex + ASI/GLS	611 / 32,468	1.16 (1.08–1.25)***		15.2***	0.710 (↑ 0.003)	2.0%
Clinical features + ASI/GLS	611 / 32,468	1.15 (1.07–1.24)***		12.7***	0.730 (↑ 0.002)	1.4%
Clinical features + LAVmax-I + LAEF + ASI/GLS	594 / 31,857	1.05 (0.98–1.13)		1.7	0.763 (↑ 0.001)	-3.5%
Age + Sex + ePWV/GLS	670 / 35,844	1.44 (1.34–1.55)***		81.9***	0.721 (↑ 0.017)***	7.1%***
Clinical features + ePWV/GLS	670 / 35,844	1.41 (1.31–1.52)***		72.3***	0.737 (↑ 0.012)**	7.3%**
Clinical features + LAVmax-I + LAEF + ePWV/GLS	651 / 35,168	1.19 (1.10–1.29)***		18.2***	0.763 (↑ 0.003)*	2.6%
Age + Sex + LVESV/LVSV	695 / 36,897	1.24 (1.16–1.32)***		36.8***	0.717 (↑ 0.009)**	5.6%*
Clinical features + LVESV/LVSV	695 / 36,897	1.24 (1.16–1.32)***		37.7***	0.738 (↑ 0.010)**	6.8%**
Clinical features + LAVmax-I + LAEF + LVESV/LVSV	660 / 35,589	1.06 (0.99–1.14)		2.8	0.765 (↑ 0.001)	-1.9%
*Incident Stroke (Case = 331, Total = 37,562)*						
Age + Sex + ASI/GLS	282 / 32,833	1.19 (1.07–1.32)**		9.6**	0.684 (↑ 0.004)	5.3%
Clinical features + ASI/GLS	282 / 32,833	1.18 (1.06–1.32)**		9.0**	0.700 (↑ 0.004)	3.9%
Clinical features + LAVmax-I + ASI/GLS	278 / 32,213	1.18 (1.06–1.31)**		8.8**	0.707 (↑ 0.004)	3.2%
Age + Sex + ePWV/GLS	312 / 36,220	1.28 (1.14–1.43)***		15.9***	0.674 (↑ 0.007)	7.2%*
Clinical features + ePWV/GLS	312 / 36,220	1.24 (1.11–1.39)***		12.5***	0.689 (↑ 0.008)	4.8%
Clinical features + LAVmax-I + ePWV/GLS	308 / 35,534	1.22 (1.09–1.37)***		10.9***	0.693 (↑ 0.006)	3.4%
Age + Sex + LVESV/LVSV	320 / 37,270	1.17 (1.05–1.29)**		8.4**	0.665 (↑ 0.008)*	6.5%
Clinical features + LVESV/LVSV	320 / 37,270	1.16 (1.05–1.28)**		8.2**	0.680 (↑ 0.008)*	6.5%*
Clinical features + LAVmax-I + LVESV/LVSV	310 / 35,952	1.13 (1.02–1.26)*		5.6*	0.686 (↑ 0.004)	4.2%
*Incident HF (Case = 255, Total = 37,965)*						
Age + Sex + ASI/GLS	220 / 33,167	1.31 (1.17–1.47)***		19.8***	0.738 (↑ 0.007)	9.7%*
Clinical features + ASI/GLS	220 / 33,167	1.27 (1.14–1.43)***		16.4***	0.789 (↑ 0.010)*	11.0%*
Clinical features + LVGFI + ASI/GLS	218 / 33,049	0.96 (0.85–1.08)		0.5	0.823 (↓ 0.001)	10.5%
Clinical features + LVEDVI + LVEF + ASI/GLS	203 / 32,518	1.00 (0.88–1.14)		<0.1	0.846 (↑ <0.001)*	-6.9%
Age + Sex + ePWV/GLS	230 / 36,603	1.75 (1.57–1.94)***		84.5***	0.777 (↑ 0.052)***	22.6%***
Clinical features + ePWV/GLS	230 / 36,603	1.67 (1.51–1.85)***		75.1***	0.818 (↑ 0.035)***	21.7%***
Clinical features + LVGFI + ePWV/GLS	229 / 36,479	1.26 (1.11–1.43)***		11.6***	0.831 (↑ 0.005)*	-6.4%
Clinical features + LVEDVI + LVEF + ePWV/GLS	212 / 35,897	1.14 (0.99–1.31)		3.2	0.855 (↑ 0.004)*	-13.3%
Age + Sex + LVESV/LVSV	224 / 37,681	1.82 (1.67–1.99)***		137.0***	0.799 (↑ 0.067)***	24.4%***
Clinical features + LVESV/LVSV	224 / 37,681	1.76 (1.61–1.91)***		126.9***	0.835 (↑ 0.049)***	24.1%***
*Incident CHD (Case = 600, Total = 36,131)*						
Age + Sex + ASI/GLS	537 / 31,593	1.03 (0.95–1.12)		0.5	0.641 (↑ <0.001)	0.7%
Clinical features + ASI/GLS	537 / 31,593	1.00 (0.92–1.09)		<0.1	0.685 (↑ <0.001)	-0.2%
Clinical features + LVESVI + ASI/GLS	522 / 30,990	1.00 (0.92–1.09)		<0.1	0.688 (↑ <0.001)	-1.2%
Clinical features + LVEDVI + LVEF + ASI/GLS	522 / 30,990	0.99 (0.91–1.08)		<0.1	0.689 (↑ <0.001)	4.7%
Age + Sex + ePWV/GLS	566 / 34,838	1.17 (1.07–1.28)***		10.9***	0.650 (↑ 0.006)*	4.2%
Clinical features + ePWV/GLS	566 / 34,838	1.13 (1.03–1.24)**		6.6*	0.690 (↑ 0.003)*	4.0%
Clinical features + LVESVI + ePWV/GLS	551 / 34,182	1.12 (1.01–1.23)*		4.8*	0.692 (↑ 0.002)	2.8%
Clinical features + LVEDVI + LVEF + ePWV/GLS	551 / 34,182	1.11 (1.001–1.23)*		3.8*	0.692 (↑ 0.001)	3.6%
Age + Sex + LVESV/LVSV	593 / 35,868	1.13 (1.05–1.22)**		10.0**	0.646 (↑ 0.004)	1.5%
Clinical features + LVESV/LVSV	593 / 35,868	1.14 (1.06–1.23)***		11.9***	0.691 (↑ 0.004)	3.0%

Hazard ratios (HRs) are expressed per 1-standard deviation (SD) increase in VAC parameters. Clinical features included age, sex, race, education, body mass index, smoking status, alcohol intake frequency, healthy physical activity, family history of heart disease, prevalent hypertension, dyslipidemia, diabetes, and CHD (excluded in the models predicting incident CHD). Multivariable Cox proportional hazards models were used. The proportional hazards assumption was not violated. All variance inflation factors were < 4; notably, LVESV/LVSV was not included in models with LVGFI, LVEF, LVESVI or LVEDVI due to their mathematical derivation and resulting multicollinearity. Values in parentheses for C-index indicate the absolute change (↑ increase, ↓decrease) compared to the corresponding reference model without VAC. Bold values for HR indicate statistical significance after multiple testing correction (FDR q-value < 0.05).

*AF* atrial fibrillation, *ASI/GLS* ratio of arterial stiffness index to global longitudinal strain, *CHD* coronary heart disease, *CI* confidence interval, *ePWV/GLS* ratio of estimated pulse wave velocity to global longitudinal strain, *FDR* false discovery rate, *HF* heart failure, *HR* hazard ratio, *LAEF* left atrial ejection fraction, *LAVmax-I* maximal left atrial volume index, *LRT* likelihood ratio test, *LVEDVI* left ventricular end-diastolic volume index, *LVEF* left ventricular ejection fraction, *LVESVI* left ventricular end-systolic volume index, *LVESV/LVSV* ratio of left ventricular end-systolic volume to stroke volume, *LVGFI* left ventricular global function index, *NRI* net reclassification improvement, *SD* standard deviation, *VAC* ventricular-arterial coupling

*** *P* < 0.001

** *P* < 0.01

* *P* < 0.05

**Table 3:** Multivariable association and incremental value of VAC parameters for predicting All-Cause and CVD mortality

Multivariable Model	Association of VAC with outcome			Incremental predictive value of VAC		
	Case / Total	HR (95% CI)		LRT Δχ²	C-index (Change)	Total NRI
*All-Cause Mortality (Case = 707, Total = 38,144)*						
Age + sex + ASI/GLS	618 / 33,314	1.14 (1.06–1.22)***		11.7***	0.724 (↑ 0.001)	4.4% *
Clinical features + ASI/GLS	618 / 33,314	1.11 (1.04–1.20)**		8.1**	0.739 (↑ 0.002)	3.6%
Clinical features + LVMI + ASI/GLS	591 / 32,659	1.12 (1.04–1.20)**		8.1**	0.738 (↑ 0.002)	4.0%
Clinical features + LVMI + LVEF + ASI/GLS	591 / 32,659	1.08 (1.003–1.17)*		4.0*	0.739 (↑ 0.001)	-0.2%
Age + sex + ePWV/GLS	659 / 36,761	1.18 (1.09–1.28)***		15.9***	0.725 (↑ 0.003)	5.8% *
Clinical features + ePWV/GLS	659 / 36,761	1.16 (1.07–1.26)***		13.0***	0.740 (↑ 0.003)	4.3%
Clinical features + LVMI + ePWV/GLS	630 / 36,048	1.16 (1.07–1.26)***		11.6***	0.738 (↑ 0.002)	3.7%
Clinical features + LVMI + LVEF + ePWV/GLS	630 / 36,048	1.12 (1.02–1.22)*		5.4*	0.739 (↑ 0.001)	0.1%
Age + sex + LVESV/LVSV	686 / 37,841	1.15 (1.07–1.23)***		14.5***	0.720 (↑ 0.001)	8.0% **
Clinical features + LVESV/LVSV	686 / 37,841	1.13 (1.06–1.21)***		12.8***	0.736 (↑ 0.002)	7.7% **
Clinical features + LVMI + LVESV/LVSV	664 / 37,191	1.14 (1.06–1.22)***		13.2***	0.736 (↑ 0.002)	7.6% ***
*CVD Mortality (Case = 113, Total = 38,144)*						
Age + sex + ASI/GLS	93 / 33,314	1.35 (1.11–1.63)**		10.7**	0.735 (↑ 0.006)	13.1%
Clinical features + ASI/GLS	93 / 33,314	1.32 (1.09–1.60)**		9.3**	0.763 (↑ 0.005)	9.2%
Clinical features + LVMI + ASI/GLS	88 / 32,659	1.30 (1.08–1.58)**		8.5**	0.770 (↑ 0.003)	9.7%
Clinical features + LVMI + LVEF + ASI/GLS	88 / 32,659	1.14 (0.94–1.39)		2.0	0.782 (↑ 0.000)	0.5%
Age + sex + ePWV/GLS	103 / 36,761	1.47 (1.22–1.76)***		16.3***	0.754 (↑ 0.019)	13.7% *
Clinical features + ePWV/GLS	103 / 36,761	1.41 (1.18–1.69)***		13.3***	0.773 (↑ 0.013)	11.3%
Clinical features + LVMI + ePWV/GLS	97 / 36,048	1.31 (1.08–1.59)**		7.0**	0.777 (↑ 0.007)	5.9%
Clinical features + LVMI + LVEF + ePWV/GLS	97 / 36,048	1.08 (0.86–1.35)		0.5	0.786 (↑ 0.001)	-3.2%
Age + sex + LVESV/LVSV	106 / 37,841	1.53 (1.33–1.76)***		29.4***	0.758 (↑ 0.022)	17.2% ***
Clinical features + LVESV/LVSV	106 / 37,841	1.51 (1.32–1.73)***		28.1***	0.785 (↑ 0.020)*	17.2% **
Clinical features + LVMI + LVESV/LVSV	101 / 37,191	1.47 (1.28–1.70)***		23.5***	0.791 (↑ 0.015)	16.8% *

Hazard ratios (HRs) are expressed per 1-standard deviation (SD) increase in VAC parameters. Clinical features included age, sex, race, education, body mass index, smoking status, alcohol intake frequency, healthy physical activity, family history of heart disease, prevalent hypertension, dyslipidemia, diabetes, and CHD. Multivariable Cox proportional hazards models were used for all-cause mortality. † For CVD mortality, Fine-Gray subdistribution hazard models were used to account for non-CVD death as a competing risk. The proportional hazards assumption was not violated. All variance inflation factors were < 4; notably, LVESV/LVSV was not included in models with LVEF due to their mathematical derivation and resulting multicollinearity. Values in parentheses for C-index indicate the absolute change (↑ increase, ↓decrease) compared to the corresponding reference model without VAC. Bold values for HR indicate statistical significance after multiple testing correction (FDR q-value < 0.05).

*ASI/GLS* ratio of arterial stiffness index to global longitudinal strain, *CHD* coronary heart disease, *CI* confidence interval, *CVD* cardiovascular disease, *ePWV/GLS* ratio of estimated pulse wave velocity to global longitudinal strain, *FDR* false discovery rate, *HR* hazard ratio, *LRT* likelihood ratio test, *LVEF* left ventricular ejection fraction, *LVESV/LVSV* ratio of left ventricular end-systolic volume to stroke volume, *LVMI* left ventricular mass index, *NRI* net reclassification improvement, *VAC* ventricular-arterial coupling

*** *P* < 0.001

* *P* < 0.05

** *P* < 0.01

#### 3.2.1 VAC and incident AF

After adjustment for clinical features, all three VAC parameters were significantly associated with incident AF (Table 2). The HRs per 1-SD increase were 1.15 (95% CI: 1.07–1.24) for ASI/GLS, 1.41 (95% CI: 1.31–1.52) for ePWV/GLS, and 1.24 (95% CI: 1.16–1.32) for LVESV/LVSV. The addition of ePWV/GLS and LVESV/LVSV raised the C-indices to 0.737 and 0.738, with corresponding NRIs of 7.3% and 6.8%, respectively, whereas ASI/GLS did not yield such improvements. Notably, the association of ePWV/GLS with AF remained robust after further adjustment for LAVmax-I and LAEF. In this fully adjusted model, adding ePWV/GLS improved model fit (LRT Δχ² = 18.2, *P* < 0.001) and the C-index to 0.763, although the NRI improvement did not reach statistical significance. Conversely, the associations of ASI/GLS and LVESV/LVSV with AF risk were non-significant after further adjusting for LAVmax-I and LAEF.

RCS analyses indicated non-linear associations between all three VAC parameters and new-onset AF (Supplementary Fig. S1). When categorized into quartiles, ASI/GLS exhibited a U-shaped pattern (lowest risk in Q3) with elevated risk in both Q1 (HR 1.40, 95% CI: 1.10–1.78) and Q4 (HR 1.47, 95% CI: 1.18–1.83). In contrast, the risk peaked in Q4 for both ePWV/GLS (HR 2.51, 95% CI: 1.77–3.57 vs Q1) and LVESV/LVSV (HR 1.52, 95% CI: 1.23–1.87 vs Q2) (Supplementary Table S6 and Fig. S2). All three VAC parameters substantially improved model fit over their constituent components (LRT Δχ² = 34.2, 28.3, 29.9, respectively; all *P* < 0.001; Supplementary Fig. S3).

#### 3.2.2 VAC and incident Stroke

All three VAC parameters were strongly associated with incident stroke when adjusted for clinical features (Table 2). The HRs per 1-SD increase were 1.18 (95% CI: 1.06–1.32) for ASI/GLS, 1.24 (95% CI: 1.11–1.39) for ePWV/GLS, and 1.16 (95% CI: 1.05–1.28) for LVESV/LVSV. These associations remained significant after further adjustment for LAVmax-I, with the inclusion of VAC parameters improving model fit (all LRT *P* < 0.05) despite non-significant improvements in the C-index and NRI. RCS analyses revealed a non-linear relationship exclusively for ASI/GLS (*P* for non-linearity = 0.029), with the lowest risk observed in Q2 (Supplementary Fig. S4–S5 and Table S6). Additionally, ASI/GLS was the only parameter that nominally improved model fit over its constituent components (LRT Δχ² = 4.8, *P* = 0.029) (Supplementary Fig. S6).

#### 3.2.3 VAC and incident HF

When adjusted for clinical features, all three VAC parameters were markedly associated with new-onset HF, with HRs per 1-SD increase of 1.27 (95% CI: 1.14–1.43) for ASI/GLS, 1.67 (95% CI: 1.51–1.85) for ePWV/GLS, and 1.76 (95% CI: 1.61–1.91) for LVESV/LVSV. The inclusion of each VAC parameter significantly improved the C-index, NRI, and model fit (Table 2). Specifically, the C-indices increased to 0.789 for ASI/GLS, 0.818 for ePWV/GLS, and 0.835 for LVESV/LVSV, accompanied by NRIs of 11.0%, 21.7%, and 24.1%, respectively. Model fit was consistently enhanced (LRT Δχ² = 16.4, 75.1, and 126.9, respectively; all *P* < 0.001). Furthermore, the association of ePWV/GLS with HF risk remained robust after further adjustment for LVGFI (HR 1.26, 95% CI: 1.11–1.43), with sustained improvements in model fit and discrimination. However, this association was non-significant when adjusted for LVEDVI and LVEF. In contrast, ASI/GLS failed to demonstrate incremental value over these conventional CMR metrics.

Non-linear relationships were found for ASI/GLS (*P* for non-linearity = 0.014) and LVESV/LVSV (*P* for non-linearity = 0.032), but not for ePWV/GLS (Supplementary Fig. S7). The risk of HF was markedly elevated in Q4 across all VAC parameters (Supplementary Table S6 and Fig. S8). Specifically, the HRs for Q4 were 1.66 (95% CI: 1.13–2.44) for ASI/GLS (vs Q2); 4.61 (95% CI: 2.39–8.87) for ePWV/GLS (vs Q1); and 3.75 (95% CI: 2.55–5.53) for LVESV/LVSV (vs Q1). Only LVESV/LVSV improved model fit over its constituent components, conditional on clinical features (Supplementary Fig. S9).

#### 3.2.4 VAC and incident CHD

After adjustment for clinical features, ePWV/GLS and LVESV/LVSV were associated with increased risk of CHD and improved model fit, whereas ASI/GLS was not (Table 2). The HRs per 1-SD increase were 1.13 (95% CI: 1.03–1.24) for ePWV/GLS and 1.14 (95% CI: 1.06–1.23) for LVESV/LVSV. Furthermore, after further adjustment for LVESVI or the combination of LVEDVI and LVEF, ePWV/GLS retained its association with CHD and continued to improve model fit. No non-linear associations were detected, nor did any of the VAC parameters yield incremental value over their constituent components for predicting CHD (Supplementary Fig. S10–S12 and Table S6).

#### 3.2.5 VAC and mortality

All three VAC parameters were robustly associated with all-cause mortality, both after adjustment for clinical features and further adjustment for LVMI alone or in combination with LVEF, and consistently improved model fit (Table 3). The HRs per 1-SD increase from the clinical features-adjusted model were 1.11 (95% CI: 1.04–1.20) for ASI/GLS, 1.16 (95% CI: 1.07–1.26) for ePWV/GLS, and 1.13 (95% CI: 1.06–1.21) for LVESV/LVSV. Although the lowest risk was observed in Q2 for ASI/GLS (Supplementary Table S7), no non-linear relationship was found for any of the three VAC parameters, nor did any of them yield incremental value over their constituent components (Supplementary Figs. S13-S15).

Regarding CVD mortality, all three VAC parameters were associated with the outcome and improved model fit even after further adjustment for LVMI (Table 3). However, these associations were attenuated to non-significance when LVEF was additionally adjusted. The HRs per 1-SD increase from the clinical features-adjusted model were 1.32 (95% CI: 1.09–1.60) for ASI/GLS, 1.41 (95% CI: 1.18–1.69) for ePWV/GLS, and 1.51 (95% CI: 1.32–1.73) for LVESV/LVSV. RCS analysis revealed a non-linear association only for ASI/GLS (*P* for non-linearity = 0.004). Risk was substantially elevated in Q4 for ASI/GLS (HR 2.44 [95% CI: 1.29–4.61] vs Q2), ePWV/GLS (HR 3.16 [95% CI: 1.08–9.26] vs Q1), and LVESV/LVSV (HR 3.62 [95% CI: 1.89–6.90] vs Q1) (Supplementary Table S7). However, none of the VAC parameters statistically improved model fit beyond their constituent components for CVD mortality (Supplementary Fig. S16-S18). Cause-specific associations of VAC with CVD and non-CVD mortality were displayed in Supplementary Table S8.

### 3.3 Additional analysis and sensitivity analyses

The E-values for unmeasured confounding assessment are presented in Supplementary Table S9. Subgroup analyses were performed across factors including age, sex, BMI, hypertension, and dyslipidemia (Supplementary Fig. S19), where associations with incident HF remained robust across almost all strata. Pearson correlation analysis revealed the inter-relationships between arterial stiffness, CMR metrics, and VAC parameters (Supplementary Fig. S20). A high correlation was observed between ASI/GLS and ePWV/GLS (r = 0.811), partly attributable to the shared GLS component. LVESV/LVSV correlated weakly with ASI/GLS (r = 0.18) and ePWV/GLS (r = 0.207), but exhibited a very strong inverse correlation with LVEF (r = −0.954). Regarding LA metrics, correlations with all VAC parameters were weak (absolute coefficients < 0.27).

## 4 Discussion

In this large, population-based cohort study, we highlight the significant predictive value of three non-invasive VAC parameters (ASI/GLS, ePWV/GLS, and LVESV/LVSV) for incident AF, stroke, HF, CHD, and both all-cause and CVD mortality (except ASI/GLS for CHD). In terms of incremental prognostic value, all VAC parameters predicted incident stroke beyond LAVmax-I, whereas ePWV/GLS specifically yielded incremental value for incident AF, HF, and CHD over traditional clinical risk factors and outcome-specific CMR markers. Furthermore, while all three VAC parameters were independently associated with all-cause mortality independent of LVEF, their associations with CVD mortality were largely attenuated by LVEF. Among all outcomes, associations with incident HF were the most consistent across clinical subgroups.

### 4.1 Interpretation of VAC parameters and their implications

The classic Ea/Ees ratio (proxied by LVESV/LVSV) and LVEF are reciprocally related by the formula: Ea/Ees = (1/LVEF) - 1 [40]. This mathematical reciprocity underpins the strong inverse correlation observed (r = −0.954), implying that LVESV/LVSV offers limited incremental value beyond LVEF [1]. In contrast, the PWV/GLS ratio may be a more appropriate measure of VAC [19]. This superiority is supported by evidence that PWV/GLS correlates more consistently with CVD risk factors, whereas the Ea/Ees ratio can exhibit paradoxical, age-dependent associations [41]. In a study of 299 hypertensive patients, echocardiography-derived PWV/GLS, rather than the Ea/Ees index, was associated with impaired carotid-intima media thickness, reduced coronary-flow reserve, and diastolic function [42]. In a post-myocardial infarction cohort with LVEF ≥40%, the PWV/GLS ratio was found to be independently associated with major adverse cardiovascular events (MACE) after adjustment, whereas the Ea/Ees ratio was not [39]. In fact, the Ea/Ees ratio is relatively insensitive in HF with preserved ejection fraction (HFpEF), because Ea is determined by non-pulsatile factors (i.e., systemic vascular resistance and heart rate) and therefore fails to capture the increased pulsatile afterload from arterial stiffness and wave reflection, which is a key pathophysiological feature of HFpEF [43]. As both Ees and Ea often increase in parallel in this condition, the resulting Ea/Ees ratio can remain deceptively normal, masking the severe underlying pathology.

Although our analysis found weak direct correlations between VAC parameters and baseline LA metrics, a well-established link exists [1]. Impaired VAC, particularly from increased arterial stiffness, leads to an earlier return of reflected pressure waves. This augments LV late-systolic load, which is a key driver of impaired LV relaxation and elevated filling pressures 1, 44. The chronic pressure overload is a significant contributor to LA remodeling and dysfunction, a common substrate for both AF and HF 45, 46.

### 4.2 VAC parameters predict CVD outcomes

The predictive value of VAC for incident AF and stroke likely stems from shared underlying mechanisms rooted in LA remodeling. This structural change, driven by VAC-induced chronic pressure overload, serves as a critical substrate for AF [45]. However, the observed U-shaped pattern of ASI/GLS suggests a "J-curve" phenomenon at the lower extreme. Given that ASI correlates more strongly with diastolic than systolic blood pressure (β = 0.24 vs 0.10) [6], extremely low values reflect compromised diastolic coronary perfusion and subsequent subclinical myocardial injury 47, 48. Conversely, ePWV/GLS exhibits a linear risk profile because its ePWV component is largely driven by age. Furthermore, impaired VAC directly impacts the cerebral vasculature, as increased arterial stiffness attenuates the aorta’s natural buffering capacity and intensifies the transmission of damaging pulsatile energy into the peripheral microcirculation, leading to microvascular damage and an increased risk of CVD [49]. This hemodynamic stress is particularly relevant for the brain's low-resistance vascular beds, where it is known to cause endothelial dysfunction and increase susceptibility to both lacunar ischemic and hemorrhagic strokes [50]. Thus, VAC dysfunction may reflect a systemic state that promotes both cardioembolic and direct microvascular pathways to stroke. This is supported by the incremental value of all VAC parameters beyond LAVmax-I demonstrated in our study.

Our study found that ePWV/GLS and LVESV/LVSV were able to predict incident CHD. These findings are consistent with the understanding that CHD and aortic stiffness share common processes, where increased aortic stiffness may impair coronary blood flow reserve 51, 52. Specifically, elevated PWV correlated with diminished coronary flow reserve in CHD patients, even after successful revascularization [52]. Moreover, lower absolute GLS was linked to a high risk of myocardial infarction [53]. Impaired VAC affects myocardial perfusion and reduces cardiac efficiency, thereby exacerbating coronary artery ischemia 54, 55. Duc et al. found that patients with stable ischemic heart disease had significantly higher VAC values (measured by Ea/Ees) than controls, which improved (i.e., VAC decreased) during the 6-month follow-up after PCI [54]. In our study, the non-significant finding for ASI/GLS may be attributed to ASI being a composite measure modulated by both central stiffness and peripheral wave reflection [6]; the latter may therefore diminish its specificity for centrally-driven CHD [56]. This distinction may help explain our varied findings, since central aortic stiffness more directly impairs diastolic coronary perfusion and promotes subendocardial ischemia [51]. In contrast, ePWV/GLS incorporates age and blood pressure—established determinants of CVD risk. Additionally, LVESV/LVSV captures LV systolic dysfunction driven by chronically elevated afterload [51].

The predictive value of all VAC parameters for HF underscores the central role of VAC in its pathogenesis. Mechanistically, this likely reflects the ability of VAC to capture the subtle, subclinical interplay between vascular and myocardial pathology that precedes overt HF, a notion supported by research linking VAC to underlying biomarker profiles of neurohormonal stress (e.g., NT-proBNP) and extracellular matrix remodeling (e.g., MMPs) 57, 58. In HF with reduced ejection fraction (HFrEF), the process is often initiated by a decline in Ees, which triggers a compensatory neurohumoral activation (e.g., sympathetic/RAAS) that in turn increases Ea 1, 59, 60. In contrast, HFpEF is frequently driven by increased arterial stiffness, which is not fully captured by the traditional Ea/Ees ratio [1]. Refining this understanding, Pugliese et al. utilized a Doppler-based method to measure aortic arch pulse wave velocity (aaPWV), which more directly assesses the properties of the aortic segment critical for ventricular-arterial interactions [61]. Their work demonstrated not only that abnormal VAC correlates with HF severity and reduced functional capacity, but also that HFpEF can exhibit even more severe VAC impairment than HFrEF when assessed via aaPWV/GLS [61]. Additionally, aaPWV may better capture aortic pressure dynamics than cfPWV, particularly in late-systolic load patterns [61].

### 4.3 VAC parameters and mortality risk

While the prognostic roles of arterial stiffness and GLS are well-established 6, 53, studies on VAC (particularly PWV/GLS) have been limited to small, high-risk populations 1, 61, leaving a gap in general population validation. Our study provides the first large-scale, general-population evidence for VAC's predictive utility. We observed independent associations with all-cause mortality for all three VAC parameters, which persisted after further adjustment for LVMI and LVEF. Regarding CVD mortality, however, although associations remained significant after adjustment for LVMI, they became non-significant upon additional adjustment for LVEF, suggesting that VAC’s impact on CVD death may be mediated by overt systolic dysfunction. This underscores the importance of optimal heart-artery interaction for long-term survival.

### 4.4 Strengths and limitations

Our study is strengthened by the large, prospective cohort and the use of multiple objective quantification methods. However, several limitations warrant consideration. First, we relied on surrogate markers for arterial stiffness (ASI and ePWV); while validated, confirmation using reference methods or more direct assessments (e.g., cfPWV/GLS) is desirable. Second, VAC parameters were derived from a single assessment at baseline, which may not capture longitudinal trajectories with aging or the progression of subclinical disease. Third, despite comprehensive adjustments and quality control, residual confounding and potential MRI measurement inaccuracies cannot be entirely ruled out. Fourth, while event numbers for most outcomes satisfied the statistical rule-of-thumb (≥10 events per variable) [62], the limited number of CVD deaths needs cautious interpretation due to potential overfitting. Fifth, given the observed non-linear patterns (e.g., the U-shaped risk for ASI/GLS in AF), linear HRs may oversimplify risks at lower extremes despite our use of RCS. Finally, the UK Biobank’s healthy volunteer bias [63] and ethnic homogeneity may limit generalizability to diverse or high-risk populations, although the robust associations observed in this low-risk cohort underscore the clinical relevance of VAC.

### 4.5 Clinical perspectives

This study underscores the clinical potential of VAC in risk prediction for various new-onset CVDs and mortality. ASI/GLS and ePWV/GLS demonstrated incremental prognostic information for stroke (beyond LAVmax-I) and all-cause mortality (beyond LVMI and LVEF). Regarding CVD mortality, all VAC parameters provided added predictive value over LVMI, although this incremental benefit was not observed when further adjusted for LVEF. Moreover, ePWV/GLS extended this utility to incident AF (over LAVmax-I and LAEF), HF (over LVGFI), and CHD (over LVESVI or the combination of LVEDVI and LVEF). Therefore, VAC parameters prove valuable for enhancing preventive strategies by identifying at-risk individuals in specific clinical scenarios.

## 5 Conclusions

In the general population, VAC parameters (ASI/GLS, ePWV/GLS, and LVESV/LVSV) are independent predictors of incident AF, stroke, HF, CHD (except for ASI/GLS), and both all-cause and CVD mortality. Importantly, ASI/GLS and ePWV/GLS yielded additional predictive value beyond conventional CMR indices for specific cardiovascular outcomes. Further research is required to validate these findings across multi-ethnic cohorts and benchmark them against reference methods such as cfPWV/GLS.

## Funding

This study was supported by the Talent Start-up Capital Program of Fujian Medical University Union Hospital (2023XH027), the Fujian Provincial Natural Science Foundation of China (2024J08178), and the Key Discipline Construction Program of Traditional Chinese Medicine in Fujian Province (2024–363).

## Author contributions

CL, YY, and QL contributed equally to this work. FH conceived and designed the study. CL performed the statistical analysis. CL, YY, and QL interpreted the derived summary results and wrote the original draft. JC, EW, SW, LL, and ML contributed to the drafting and revision of the manuscript. LF and EY provided critical insights and revised the manuscript for important intellectual content. All authors approved the final manuscript.

## Declaration of competing interests

The authors declare that they have no financial or non-financial conflicts of interest.

## Data Availability

This study was conducted using data from the UK Biobank under Application Number 216062. The UK Biobank data can be accessed by researchers on application (https://www.ukbiobank.ac.uk/ register-apply/). The graphical abstract was created using Figdraw (ID: UYSWY67729).
