# Supplementary material for: Left ventricular-arterial coupling parameters predict incident cardiovascular events and mortality in UK Biobank
Source: J Cardiovasc Magn Reson. 2026 Mar 6;28(1):102709. doi: 10.1016/j.jocmr.2026.102709 (PMC13241742; doi:10.1016/j.jocmr.2026.102709)
Supplement: Supplementary file 1 — Supplementary material [file mmc1.docx]

**Supplementary Material**

Table of contents

[Table S1. Reporting checklist based on the STROBE cohort guidelines. 2](#_Toc217421721)

[Table S2. UK Biobank field codes used to define baseline exclusion variables. 4](#_Toc217421722)

[Table S3. UK Biobank field codes used to define covariates. 5](#_Toc217421723)

[Table S4. UK Biobank field codes used to define outcomes of interest. 9](#_Toc217421724)

[Table S5. Sample sizes and detailed exclusion reasons for each multivariable model. 11](#_Toc217421725)

[Table S6. Multivariable association of quartiles of VAC parameters with incident AF, Stroke, HF, and CHD. 14](#_Toc217421726)

[Table S7. Multivariable association of quartiles of VAC parameters with all-cause and CVD mortality. 16](#_Toc217421727)

[Table S8. Cause-specific associations of VAC parameters with CVD and non-CVD mortality. 18](#_Toc217421728)

[Table S9. E-values for unmeasured confounding assessment across multivariable models. 19](#_Toc217421729)

[Figure S1. RCS analysis of the association between VAC parameters and incident AF. 22](#_Toc217421730)

[Figure S2. Cumulative incidence of AF according to quartiles of VAC parameters. 23](#_Toc217421731)

[Figure S3. Incremental value of VAC parameters over its constituent components for incident AF. 26](#_Toc217421732)

[Figure S4. RCS analysis of the association between VAC parameters and incident stroke. 30](#_Toc217421733)

[Figure S5. Cumulative incidence of stroke according to quartiles of VAC parameters. 31](#_Toc217421734)

[Figure S6. Incremental value of VAC parameters over its constituent components for incident stroke. 34](#_Toc217421735)

[Figure S7. RCS analysis of the association between VAC parameters and incident HF. 38](#_Toc217421736)

[Figure S8. Cumulative incidence of HF according to quartiles of VAC parameters. 39](#_Toc217421737)

[Figure S9. Incremental value of VAC parameters over its constituent components for incident HF. 42](#_Toc217421738)

[Figure S10. RCS analysis of the association between VAC parameters and incident CHD. 46](#_Toc217421739)

[Figure S11. Cumulative incidence of CHD according to quartiles of VAC parameters. 47](#_Toc217421740)

[Figure S12. Incremental value of VAC parameters over its constituent components for incident CHD. 50](#_Toc217421741)

[Figure S13. RCS analysis of the association between VAC parameters and all-cause mortality. 54](#_Toc217421742)

[Figure S14. Cumulative incidence of all-cause mortality according to quartiles of VAC parameters. 55](#_Toc217421743)

[Figure S15. Incremental value of VAC parameters over its constituent components for all-cause mortality. 58](#_Toc217421744)

[Figure S16. RCS analysis of the association between VAC parameters and CVD mortality. 62](#_Toc217421745)

[Figure S17. Cumulative incidence of CVD mortality according to quartiles of VAC parameters. 63](#_Toc217421746)

[Figure S18. Incremental value of VAC parameters over its constituent components for CVD mortality. 66](#_Toc217421747)

[Figure S19. Subgroup analysis of the multivariable association between VAC parameters and adverse outcomes. 70](#_Toc217421748)

[Figure S20. Pearson correlation between arterial stiffness, CMR metrics and VAC parameters. 76](#_Toc217421749)

## Table S1. Reporting checklist based on the STROBE cohort guidelines.

|  | Item No | Recommendation | Checked |
| --- | --- | --- | --- |
| **Title and abstract** | 1 | (*a*) Indicate the study’s design with a commonly used term in the title or the abstract | ✔ |
|  |  | (*b*) Provide in the abstract an informative and balanced summary of what was done and what was found | ✔ |
| Introduction | | |  |
| Background/rationale | 2 | Explain the scientific background and rationale for the investigation being reported | ✔ |
| Objectives | 3 | State specific objectives, including any prespecified hypotheses | ✔ |
| Methods | | |  |
| Study design | 4 | Present key elements of study design early in the paper | ✔ |
| Setting | 5 | Describe the setting, locations, and relevant dates, including periods of recruitment, exposure, follow-up, and data collection | ✔ |
| Participants | 6 | (*a*) Give the eligibility criteria, and the sources and methods of selection of participants. Describe methods of follow-up | ✔ |
|  |  | (*b*) For matched studies, give matching criteria and number of exposed and unexposed | N/A |
| Variables | 7 | Clearly define all outcomes, exposures, predictors, potential confounders, and effect modifiers. Give diagnostic criteria, if applicable | ✔ |
| Data sources/ measurement | 8* | For each variable of interest, give sources of data and details of methods of assessment (measurement). Describe comparability of assessment methods if there is more than one group | ✔ |
| Bias | 9 | Describe any efforts to address potential sources of bias | ✔ |
| Study size | 10 | Explain how the study size was arrived at | ✔ |
| Quantitative variables | 11 | Explain how quantitative variables were handled in the analyses. If applicable, describe which groupings were chosen and why | ✔ |
| Statistical methods | 12 | (*a*) Describe all statistical methods, including those used to control for confounding | ✔ |
|  |  | (*b*) Describe any methods used to examine subgroups and interactions | ✔ |
|  |  | (*c*) Explain how missing data were addressed | ✔ |
|  |  | (*d*) If applicable, explain how loss to follow-up was addressed | ✔ |
|  |  | (*e*) Describe any sensitivity analyses | ✔ |
| Results | | |  |
| Participants | 13* | (a) Report numbers of individuals at each stage of study—eg numbers potentially eligible, examined for eligibility, confirmed eligible, included in the study, completing follow-up, and analysed | ✔ |
|  |  | (b) Give reasons for non-participation at each stage | N/A |
|  |  | (c) Consider use of a flow diagram | ✔ |
| Descriptive data | 14* | (a) Give characteristics of study participants (eg demographic, clinical, social) and information on exposures and potential confounders | ✔ |
|  |  | (b) Indicate number of participants with missing data for each variable of interest | ✔ |
|  |  | (c) Summarise follow-up time (eg, average and total amount) | ✔ |
| Outcome data | 15* | Report numbers of outcome events or summary measures over time | ✔ |
| Main results | 16 | (*a*) Give unadjusted estimates and, if applicable, confounder-adjusted estimates and their precision (eg, 95% confidence interval). Make clear which confounders were adjusted for and why they were included | ✔ |
|  |  | (*b*) Report category boundaries when continuous variables were categorized | ✔ |
|  |  | (*c*) If relevant, consider translating estimates of relative risk into absolute risk for a meaningful time period | ✔ |
| Other analyses | 17 | Report other analyses done—eg analyses of subgroups and interactions, and sensitivity analyses | ✔ |
| Discussion | | |  |
| Key results | 18 | Summarise key results with reference to study objectives | ✔ |
| Limitations | 19 | Discuss limitations of the study, taking into account sources of potential bias or imprecision. Discuss both direction and magnitude of any potential bias | ✔ |
| Interpretation | 20 | Give a cautious overall interpretation of results considering objectives, limitations, multiplicity of analyses, results from similar studies, and other relevant evidence | ✔ |
| Generalisability | 21 | Discuss the generalisability (external validity) of the study results | ✔ |
| Other information | | |  |
| Funding | 22 | Give the source of funding and the role of the funders for the present study and, if applicable, for the original study on which the present article is based | ✔ |

The STROBE checklist is made available under the Creative Commons Attribution License (CC-BY). For further details about the STROBE Initiative, please visit http://www.strobe-statement.org. N/A, not applicable.

## **Table S2.** UK Biobank field codes used to define baseline exclusion variables.

| **Variable** | **UKB Field ID or ICD code** | **Description** |
| --- | --- | --- |
| **Valvular heart disease** |  |  |
| Rheumatic valve disease |  |  |
| Mitral valve | First occurrences (131276) | I05 |
| Aortic valve | First occurrences (131278) | I06 |
| Tricuspid valve | First occurrences (131280) | I07 |
| Congenital valve disease or Marfan's syndrome |  |  |
| Pulmonary and tricuspid valves | First occurrences (132470) | Q22 |
| Aortic and mitral valves | First occurrences (132472) | Q23 |
| Marfan's syndrome | Diagnoses - ICD10 (41270) | Q874 |
| Endocarditis with Valvular Heart Disease |  |  |
| Valve unspecified | First occurrences (131330) | I38 |
| Heart valve disorders in diseases  classified elsewhere | First occurrences (131332) | I39 |
| Nonrheumatic heart diseases |  |  |
| Mitral valve disorders | First occurrences (131322) | I34 |
| Aortic valve disorders | First occurrences (131324) | I35 |
| Tricuspid valve disorders | First occurrences (131326) | I36 |
| Pulmonary valve disorders | First occurrences (131328) | I37 |
| Multiple valve disorders | First occurrences (131282) | I08 |
| **Aortic aneurysm and dissection** | Self-report (20002) |  |
|  | 1492 | Aortic aneurysm |
|  | 1591 | Aortic aneurysm rupture |
|  | 1592 | Aortic dissection |
|  | Diagnoses - ICD 9 (41271) |  |
|  | 441 | Aortic aneurysm |
|  | Diagnoses - ICD10 (41270) |  |
|  | I71 | Aortic aneurysm and dissection |
|  | First occurrences (131382) | I71 |
| **Cardiovascular surgery** |  |  |
| **Structural and congenital heart surgery** |  |  |
| Heart transplantation | OPCS4 (41272) | K01, K02 |
| Repair of complex congenital anomalies | OPCS4 (41272) | K04-K09, K17 |
| Repair of atrial/ventricular septal  defects and other septal procedures | OPCS4 (41272) | K10-K16 |
| Creation of cardiac conduits | OPCS4 (41272) | K18, K19 |
| Repair of atrium, ventricle, and pericardium | OPCS4 (41272) | K20, K22, K23, K24, K67, K69, K71 |
| Repair of congenital anomalies of great  vessels and pulmonary artery | OPCS4 (41272) | L01-L13 |
| **Valvular heart surgery** | OPCS4 (41272) | K25-K38 |
| **Aortic root and aortic surgery** | OPCS4 (41272) | K33, L16, L18-L23, L25-L28 |
| **Coronary revascularization** |  |  |
| Surgical, e.g., CABG | OPCS4 (41272) | K40-K48 |
| Percutaneous | OPCS4 (41272) | K49, K50, K75 |

Note: Three-digit ICD codes encompass all corresponding four-digit subcodes (e.g., I05 includes I05.0–I05.9). Abbreviations: ICD, International Classification of Diseases; OPCS-4, Office of Population Censuses and Surveys Classification of Interventions and Procedures version 4.

## Table S3. UK Biobank field codes used to define covariates.

| **Variable** | **UKB Field ID or ICD code** | **Description** |  |
| --- | --- | --- | --- |
| Age | 21003 | First imaging visit |  |
| Sex | 31 | - |  |
| Race | 21000 | - |  |
| BMI | 23104 | Via body composition by impedance |  |
|  | 21001 | Via body size measures |  |
| Smoking status | 20116 | - |  |
| Alcohol intake frequency | 1558 | - |  |
| Healthy physical activity | 100054 | Physical activity |  |
| Family history of heart diseases | 20107, 20110 | Illness of parents |  |
| Hypertension | Self-report (20002) |  |  |
|  | 1065 | Hypertension |  |
|  | 1072 | Essential hypertension |  |
|  | Diagnosed by doctor |  |  |
|  | 6150: 4 | High blood pressure |  |
|  | 2966 | Age high blood pressure diagnosed |  |
|  | Diagnoses – ICD 9 (41271) |  |  |
|  | 401-405 |  |  |
|  | Diagnoses – ICD10 (41270) |  |  |
|  | I10 | Essential (primary) hypertension |  |
|  | I11 | Hypertensive heart disease |  |
|  | I12 | Hypertensive renal disease |  |
|  | I13 | Hypertensive heart and renal disease |  |
|  | I15 | Secondary hypertension |  |
|  | First occurrences |  |  |
|  | 131286, 131288, 131290,  131292, 131294 | I10, I11, I12, I13, I15 |  |
|  | Antihypertensive medication | As below |  |
| Dyslipidemia | Self-reported 20002: 1473 | High cholesterol |  |
|  | Diagnoses – ICD 9 (41271)  272 | Disorders of lipoid metabolism |  |
|  | Diagnoses – ICD10 (41270) |  |  |
|  | E780 | Pure hypercholesterolaemia |  |
|  | E781 | Pure hyperglyceridaemia |  |
|  | E782 | Mixed hyperlipidaemia |  |
|  | E783 | Hyperchylomicronaemia |  |
|  | E784 | Other hyperlipidaemia |  |
|  | E785 | Hyperlipidaemia, unspecified |  |
|  | First occurrences |  |  |
|  | 130814 | E78, disorders of lipoprotein metabolism and other lipidaemias |  |
|  | Lipid-lowering medication | As below |  |
| Diabetes | Self-report (20002) |  |  |
|  | 1220 | Diabetes |  |
|  | 1222 | Type 1 diabetes |  |
|  | 1223 | Type 2 diabetes |  |
|  | Diagnosed by doctor |  |  |
|  | 2443 | Diabetes diagnosed by doctor |  |
|  | 2976 | Age diabetes diagnosed by doctor |  |
|  | Diagnoses – ICD 9 (41271)  250 | Diabetes mellitus |  |
|  | Diagnoses – ICD10 (41270) |  |  |
|  | E10 | Type 1 diabetes mellitus |  |
|  | E11 | Type 2 diabetes mellitus |  |
|  | E13 | Other specified diabetes mellitus |  |
|  | E14 | Unspecified diabetes mellitus |  |
|  | G590 | Diabetic mononeuropathy |  |
|  | G632 | Diabetic polyneuropathy |  |
|  | H280 | Diabetic cataract |  |
|  | H360 | Diabetic retinopathy |  |
|  | M142 | Diabetic arthropathy |  |
|  | N083 | Glomerular disorders in diabetes mellitus |  |
|  | O240 | Diabetes mellitus in pregnancy: Pre-existing type 1 diabetes mellitus |  |
|  | O241 | Diabetes mellitus in pregnancy: Pre-existing type 2 diabetes mellitus |  |
|  | O243 | Diabetes mellitus in pregnancy: Pre-existing diabetes mellitus, unspecified |  |
|  | O244 | Diabetes mellitus arising in pregnancy |  |
|  | O249 | Diabetes mellitus in pregnancy, unspecified |  |
|  | Y423 | Insulin and oral hypoglycaemic [antidiabetic] drugs |  |
|  | First occurrences (130706, 130708, 130712, 130714) | E10, E11, E13, E14 |  |
|  | HbA1c (30750, Glycated  haemoglobin) | ≥6.5% |  |
|  | Anti-diabetic medication | As below |  |
| Anti-diabetic medication | Medications (6153, 6177): 3 | Insulin |  |
|  | Self-reported medications (20003) | 1140884600, 1140874744, 1141152590, 1140874646, 1140874674, 1141171646, 1140883066,  1141168660 |  |
| Lipid-lowering medication | Medications (6153, 6177): 1 | Cholesterol lowering medication |  |
|  | Self-reported medications (20003) | 1141146138, 1141146234, 1141192414, 1140861958, 1140881748, 1141200040, 1140888648, 1141192410, 1140864592, 1140888594, 1140861954, 1140861924, 1141157260, 1140862026, 1140861944, 1140862026, 1140861928, 1140861926, 1141162544 |  |
| Antihypertensive medication | Medications (6153, 6177): 2 | Blood pressure medication |  |
|  | Self-reported medications (20003) | 1140860334, 1140860336, 1140860338, 1140860340, 1140860342, 1140860348, 1140860352, 1140860356, 1140860358, 1140860380, 1140860382, 1140860386, 1140860390, 1140860394, 1140860396, 1140860398, 1140860402, 1140860404, 1140860406, 1140860410, 1140860418, 1140860422, 1140860426, 1140860434, 1140860492, 1140860498, 1140860562, 1140860564, 1140860580, 1140860590, 1140860610, 1140860654, 1140860658, 1140860690, 1140860696, 1140860706, 1140860714, 1140860728, 1140860736, 1140860738, 1140860750, 1140860752, 1140860758, 1140860764, 1140860776, 1140860784, 1140860790, 1140860802, 1140860806, 1140860878, 1140860882, 1140860892, 1140860904, 1140860912, 1140860918, 1140861088, 1140861090, 1140861106, 1140861110, 1140861114, 1140861120, 1140861128, 1140861130, 1140861136, 1140861138, 1140861166, 1140861176, 1140861190, 1140861194, 1140861202, 1140861276, 1140861282, 1140864950, 1140864952, 1140866072, 1140866078, 1140866090, 1140866092, 1140866094, 1140866096, 1140866102, 1140866104, 1140866122, 1140866128, 1140866132, 1140866136, 1140866138, 1140866140, 1140866144, 1140866146, 1140866156, 1140866158, 1140866162, 1140866164, 1140866168, 1140866226, 1140866232, 1140866236, 1140866244, 1140866262, 1140866306, 1140866308, 1140866312, 1140866318, 1140866324, 1140866328, 1140866330, 1140866340, 1140866352, 1140866354, 1140866360, 1140866396, 1140866400, 1140866402, 1140866404, 1140866410, 1140866416, 1140866420, 1140866440, 1140866446, 1140866450, 1140866460, 1140866466, 1140866484, 1140866546, 1140866554, 1140866692, 1140866704, 1140866712, 1140866724, 1140866726, 1140866738, 1140866756, 1140866758, 1140866764, 1140866766, 1140866778, 1140866782, 1140866784, 1140866798, 1140866800, 1140866802, 1140866804, 1140879760, 1140879762, 1140879778, 1140879782, 1140879786, 1140879794, 1140879798, 1140879802, 1140879806, 1140879810, 1140879818, 1140879824, 1140879830, 1140879834, 1140879842, 1140879866, 1140888510, 1140888552, 1140888556, 1140888560, 1140888578, 1140888646, 1140909368, 1140911698, 1140916356,  1140916362, 1140917428, 1140923572, 1140923712, 1140923718, 1140926778, 1140926780, 1141145658, 1141145660, 1141145668, 1141151016, 1141151018, 1141151382, 1141152600, 1141152998, 1141153006, 1141153026, 1141153032, 1141153328, 1141156754, 1141156808, 1141156836, 1141156846, 1141164148, 1141164154, 1141164276, 1141164280, 1141165470, 1141165476, 1141166006, 1141167822, 1141167832, 1141171152, 1141171336, 1141171344, 1141172682, 1141172686, 1141180592, 1141180598, 1141187788, 1141187790, 1141190160, 1141193282, 1141193346, 1141194794, 1141194800, 1141194804, 1141194808, 1141194810, 1141201038, 1141201040 |  |
|  |  |  |  |

Note that three-digit codes encompass all corresponding four-digit subcodes, such as I11 (I110–I119). Abbreviations: ICD, International Classification of Diseases.

## **Table S4. UK Biobank field codes used to define outcomes of interest**.

| **Outcome** | **UKB Field ID or ICD code** | **Description** |
| --- | --- | --- |
| Atrial fibrillation | Self-report (20002) |  |
|  | 1471 | Atrial fibrillation |
|  | 1483 | Atrial flutter |
|  | Diagnoses - ICD 9 (41271) |  |
|  | 4273 | Atrial fibrillation and flutter |
|  | Diagnoses - ICD10 (41270) |  |
|  | I48 | Atrial fibrillation and flutter |
|  | First occurrences |  |
|  | 131350 | I48, Atrial fibrillation and flutter |
| Stroke | Self-report (20002) |  |
|  | 1081 | stroke |
|  | 1086 | subarachnoid haemorrhage |
|  | 1491 | brain haemorrhage |
|  | 1583 | ischaemic stroke |
|  | Diagnosed by doctor (6150) | Stroke |
|  | 3 |  |
|  | Diagnoses - ICD 9 (41271) |  |
|  | 430, 431, 434, 436 |  |
|  | Diagnoses - ICD10 (41270) |  |
|  | I60, I61, I63, I64 |  |
|  | First occurrences |  |
|  | 131360, 131362,   131366, 131368 | I60, I61, I63, I64 |
| Heart failure | Self-report (20002) |  |
|  | 1076 | heart failure/pulmonary odema |
|  | Diagnoses - ICD 9 (41271) |  |
|  | 428 | Heart failure |
|  | Diagnoses - ICD10 (41270) |  |
|  | I50, I110, I130, I132 |  |
|  | First occurrences |  |
|  | 131354 | I50, heart failure |
| Coronary heart disease | Self-report (20002) |  |
|  | 1074 | angina |
|  | 1075 | heart attack/myocardial infarction |
|  | Diagnosed by doctor (6150) |  |
|  | 1, 2 |  |
|  | Age angina diagnosed (3627) |  |
|  | Age heart attack diagnosed (2976) |  |
|  | Diagnoses - ICD 9 (41271) |  |
|  | 410-414 |  |
|  | Diagnoses - ICD10 (41270) |  |
|  | I20, I21, I22, I23, I24, I25,   Z951, Z955 |  |
|  | First occurrences |  |
|  | 131296, 131298, 131300,   131302, 131304, 131306 | I20, I21, I22, I23, I24, I25 |
| All-Cause mortality | Date of death register (40000) |  |
| CVD mortality | Primary cause of death (40001) | I00-I99 |
|  | Date of death register (40000) |  |

Note: Three-digit ICD codes encompass all corresponding four-digit subcodes (e.g., I48 includes I480–I489). Self-reported diagnoses (including those reported as "diagnosed by doctor") were utilized exclusively to identify and exclude prevalent cases at baseline. Abbreviations: ICD, International Classification of Diseases.

## Table S5. Sample sizes and detailed exclusion reasons for each multivariable model.

| **Model** | **Sample size  included** | **Number and reason for exclusion** |
| --- | --- | --- |
| **Incident AF** | | |
| Age + Sex + ASI/GLS | 32,468 | 4,758 missing ASI or GLS; 23 statistical outliers of ASI or GLS;  856 with prevalent AF; 39 statistical outliers of VAC |
| Clinical features + ASI/GLS | 32,468 | 4,758 missing ASI or GLS; 23 statistical outliers of ASI or GLS;  856 with prevalent AF; 39 statistical outliers of VAC |
| Clinical features + LAVmax-I  + LAEF + ASI/GLS | 31,857 | 4,758 missing ASI or GLS; 23 statistical outliers of ASI or GLS;  856 with prevalent AF; 613 missing LAVmax-I or LAEF;  37 statistical outliers of VAC |
| Age + Sex + ePWV/GLS | 35,844 | 1,238 missing ePWV or GLS; 13 statistical outliers of ePWV or GLS;  952 with prevalent AF; 97 statistical outliers of VAC |
| Clinical features + ePWV/GLS | 35,844 | 1,238 missing ePWV or GLS; 13 statistical outliers of ePWV or GLS;  952 with prevalent AF; 97 statistical outliers of VAC |
| Clinical features + LAVmax-I  + LAEF + ePWV/GLS | 35,168 | 1,238 missing ePWV or GLS; 13 statistical outliers of ePWV or GLS;  952 with prevalent AF; 679 missing LAVmax-I or LAEF;  94 statistical outliers of VAC |
| Age + Sex + LVESV/LVSV | 36,897 | 132 missing LVESV or LVSV; 44 statistical outliers of LVESV or LVSV;  974 with prevalent AF; 97 statistical outliers of VAC |
| Clinical features + LVESV/LVSV | 36,897 | 132 missing LVESV or LVSV; 44 statistical outliers of LVESV or LVSV;  974 with prevalent AF; 97 statistical outliers of VAC |
| Clinical features + LAVmax-I + LAEF + LVESV/LVSV | 35,589 | 132 missing LVESV or LVSV; 44 statistical outliers of LVESV or LVSV;  974 with prevalent AF; 1,313 missing LAVmax-I or LAEF; 92 statistical outliers of VAC |
|  |  |  |
| **Incident Stroke** | | |
| Age + Sex + ASI/GLS | 32,833 | 4,758 missing ASI or GLS; 23 statistical outliers of ASI or GLS;  483 with prevalent Stroke; 47 statistical outliers of VAC |
| Clinical features + ASI/GLS | 32,833 | 4,758 missing ASI or GLS; 23 statistical outliers of ASI or GLS;  483 with prevalent Stroke; 47 statistical outliers of VAC |
| Clinical features + LAVmax-I  + ASI/GLS | 32,213 | 4,758 missing ASI or GLS; 23 statistical outliers of ASI or GLS;  483 with prevalent Stroke; 622 missing LAVmax-I; 45 statistical outliers of VAC |
| Age + Sex + ePWV/GLS | 36,220 | 1,238 missing ePWV or GLS; 13 statistical outliers of ePWV or GLS;  549 with prevalent Stroke; 124 statistical outliers of VAC |
| Clinical features + ePWV/GLS | 36,220 | 1,238 missing ePWV or GLS; 13 statistical outliers of ePWV or GLS;  549 with prevalent Stroke; 124 statistical outliers of VAC |
| Clinical features + LAVmax-I  + ePWV/GLS | 35,534 | 1,238 missing ePWV or GLS; 13 statistical outliers of ePWV or GLS;  549 with prevalent Stroke; 690 missing LAVmax-I;  120 statistical outliers of VAC |
| Age + Sex + LVESV/LVSV | 37,270 | 132 missing LVESV or LVSV; 44 statistical outliers of LVESV or LVSV;  577 with prevalent Stroke; 121 statistical outliers of VAC |
| Clinical features + LVESV/LVSV | 37,270 | 132 missing LVESV or LVSV; 44 statistical outliers of LVESV or LVSV;  577 with prevalent Stroke; 121 statistical outliers of VAC |
| Clinical features + LAVmax-I  + LVESV/LVSV | 35,952 | 132 missing LVESV or LVSV; 44 statistical outliers of LVESV or LVSV;  577 with prevalent Stroke; 1324 missing LAVmax-I;  115 statistical outliers of VAC |
|  |  |  |
| **Incident HF** | | |
| Age + Sex + ASI/GLS | 33,167 | 4,758 missing ASI or GLS; 23 statistical outliers of ASI or GLS;  150 with prevalent HF; 46 statistical outliers of VAC |
| Clinical features + ASI/GLS | 33,167 | 4,758 missing ASI or GLS; 23 statistical outliers of ASI or GLS;  150 with prevalent HF; 46 statistical outliers of VAC |
| Clinical features + LVGFI  + ASI/GLS | 33,049 | 4,758 missing ASI or GLS; 23 statistical outliers of ASI or GLS;  150 with prevalent HF; 117 missing LVGFI;  47 statistical outliers of VAC |
| Clinical features + LVEDVI  + LVEF + ASI/GLS | 32,518 | 4,758 missing ASI or GLS; 23 statistical outliers of ASI or GLS;  150 with prevalent HF; 651 missing LVEDVI or LVEF;  44 statistical outliers of VAC |
| Age + Sex + ePWV/GLS | 36,603 | 1,238 missing ePWV or GLS; 13 statistical outliers of ePWV or GLS;  166 with prevalent HF; 124 statistical outliers of VAC |
| Clinical features + ePWV/GLS | 36,603 | 1,238 missing ePWV or GLS; 13 statistical outliers of ePWV or GLS;  166 with prevalent HF; 124 statistical outliers of VAC |
| Clinical features + LVGFI  + ePWV/GLS | 36,479 | 1,238 missing ePWV or GLS; 13 statistical outliers of ePWV or GLS;  166 with prevalent HF; 128 missing LVGFI;  120 statistical outliers of VAC |
| Clinical features + LVEDVI  + LVEF + ePWV/GLS | 35,897 | 1,238 missing ePWV or GLS; 13 statistical outliers of ePWV or GLS;  166 with prevalent HF; 711 missing LVEDVI or LVEF;  119 statistical outliers of VAC |
| Age + Sex + LVESV/LVSV | 37,681 | 132 missing LVESV or LVSV; 44 statistical outliers of LVESV or LVSV;  170 with prevalent HF; 117 statistical outliers of VAC |
| Clinical features + LVESV/LVSV | 37,681 | 132 missing LVESV or LVSV; 44 statistical outliers of LVESV or LVSV;  170 with prevalent HF; 117 statistical outliers of VAC |
|  |  |  |
| **Incident CHD** | | |
| Age + Sex + ASI/GLS | 31,593 | 4,758 missing ASI or GLS; 23 statistical outliers of ASI or GLS;  1722 with prevalent CHD; 48 statistical outliers of VAC |
| Clinical features + ASI/GLS | 31,593 | 4,758 missing ASI or GLS; 23 statistical outliers of ASI or GLS;  1722 with prevalent CHD; 48 statistical outliers of VAC |
| Clinical features + LVESVI  + ASI/GLS | 30,990 | 4,758 missing ASI or GLS; 23 statistical outliers of ASI or GLS;  1722 with prevalent CHD; 604 missing LVESVI;  47 statistical outliers of VAC |
| Clinical features + LVEDVI  + LVEF + ASI/GLS | 30,990 | 4,758 missing ASI or GLS; 23 statistical outliers of ASI or GLS;  1722 with prevalent CHD; 604 missing LVEDVI or LVEF;  47 statistical outliers of VAC |
| Age + Sex + ePWV/GLS | 34,838 | 1,238 missing ePWV or GLS; 13 statistical outliers of ePWV or GLS;  1934 with prevalent CHD; 121 statistical outliers of VAC |
| Clinical features + ePWV/GLS | 34,838 | 1,238 missing ePWV or GLS; 13 statistical outliers of ePWV or GLS;  1934 with prevalent CHD; 121 statistical outliers of VAC |
| Clinical features + LVESVI  + ePWV/GLS | 34,182 | 1,238 missing ePWV or GLS; 13 statistical outliers of ePWV or GLS;  1934 with prevalent CHD; 661 missing LVESVI;  116 statistical outliers of VAC |
| Clinical features + LVEDVI  + LVEF + ePWV/GLS | 34,182 | 1,238 missing ePWV or GLS; 13 statistical outliers of ePWV or GLS;  1934 with prevalent CHD; 661 missing LVESVI;  116 statistical outliers of VAC |
| Age + Sex + LVESV/LVSV | 35,868 | 132 missing LVESV or LVSV; 44 statistical outliers of LVESV or LVSV;  1989 with prevalent CHD; 111 statistical outliers of VAC |
| Clinical features + LVESV/LVSV | 35,868 | 132 missing LVESV or LVSV; 44 statistical outliers of LVESV or LVSV;  1989 with prevalent CHD; 111 statistical outliers of VAC |
|  |  |  |
| **All-Cause Mortality or CVD Mortality** | | |
| Age + Sex + ASI/GLS | 33,314 | 4,758 missing ASI or GLS; 23 statistical outliers of ASI or GLS;  49 statistical outliers of VAC |
| Clinical features + ASI/GLS | 33,314 | 4,758 missing ASI or GLS; 23 statistical outliers of ASI or GLS;  49 statistical outliers of VAC |
| Clinical features + LVMI  + ASI/GLS | 32,659 | 4,758 missing ASI or GLS; 23 statistical outliers of ASI or GLS;  657 missing LVMI; 47 statistical outliers of VAC |
| Clinical features + LVMI  + LVEF + ASI/GLS | 32,659 | 4,758 missing ASI or GLS; 23 statistical outliers of ASI or GLS;  657 missing LVMI or LVEF; 47 statistical outliers of VAC |
| Age + Sex + ePWV/GLS | 36,761 | 1,238 missing ePWV or GLS; 13 statistical outliers of ePWV or GLS;  132 statistical outliers of VAC |
| Clinical features + ePWV/GLS | 36,761 | 1,238 missing ePWV or GLS; 13 statistical outliers of ePWV or GLS;  132 statistical outliers of VAC |
| Clinical features + LVMI  + ePWV/GLS | 36,048 | 1,238 missing ePWV or GLS; 13 statistical outliers of ePWV or GLS;  718 missing LVMI; 127 statistical outliers of VAC |
| Clinical features + LVMI  + LVEF + ePWV/GLS | 36,048 | 1,238 missing ePWV or GLS; 13 statistical outliers of ePWV or GLS;  718 missing LVMI or LVEF; 127 statistical outliers of VAC |
| Age + Sex + LVESV/LVSV | 37,841 | 132 missing LVESV or LVSV; 44 statistical outliers of LVESV or LVSV;  127 statistical outliers of VAC |
| Clinical features + LVESV/LVSV | 37,841 | 132 missing LVESV or LVSV; 44 statistical outliers of LVESV or LVSV;  127 statistical outliers of VAC |
| Clinical features + LVMI  + LVESV/LVSV | 37,191 | 132 missing LVESV or LVSV; 44 statistical outliers of LVESV or LVSV;  655 missing LVMI; 122 statistical outliers of VAC |

Statistical outliers were defined as values deviating by more than 4 times the interquartile range (4×IQR) from the first or third quartiles. Abbreviations: AF, atrial fibrillation; ASI, arterial stiffness index; CHD, coronary heart disease; CVD, cardiovascular disease; ePWV, estimated pulse wave velocity; GLS, global longitudinal strain; HF, heart failure; LAEF, left atrial emptying fraction; LAVmax-I, maximal left atrial volume index; LVEDVI, left ventricular end-diastolic volume index; LVEF, left ventricular ejection fraction; LVESV, left ventricular end-systolic volume; LVESVI, left ventricular end-systolic volume index; LVGFI, left ventricular global function index; LVMI, left ventricular mass index; LVSV, left ventricular stroke volume; VAC, ventricular-arterial coupling.

## Table S6. Multivariable association of quartiles of VAC parameters with incident AF, Stroke, HF, and CHD.

|  | **Incident AF** | | |  | **Incident Stroke** | | |
| --- | --- | --- | --- | --- | --- | --- | --- |
|  | **Event, n (%)** | **Incidence event rate** | **Adj. HR (95% CI)** |  | **Event, n (%)** | **Incidence event rate** | **Adj. HR (95% CI)** |
| ASI/GLS |  |  |  |  |  |  |  |
| Total | 611 (1.88%) | 3.62 (3.33–3.91) | - |  | 282 (0.86%) | 1.64 (1.46–1.84) | - |
| Q1 | 149 (1.84%) | 3.61 (3.07–4.24) | **1.40 (1.10–1.78)** |  | 60 (0.73%) | 1.43 (1.11–1.84) | 1.26 (0.86–1.85) |
| Q2 | 123 (1.52%) | 2.89 (2.42–3.44) | 1.08 (0.85–1.39) |  | 48 (0.58%) | 1.11 (0.84–1.47) | ref |
| Q3 | 129 (1.59%) | 2.98 (2.50–3.54) | ref |  | 77 (0.94%) | 1.75 (1.40–2.19) | **1.49 (1.04–2.14)** |
| Q4 | 210 (2.59%) | 5.04 (4.40–5.77) | **1.47 (1.18–1.83)** |  | 97 (1.18%) | 2.29 (1.88–2.79) | **1.75 (1.23–2.48)** |
| ePWV/GLS |  |  |  |  |  |  |  |
| Total | 670 (1.87%) | 3.66 (3.38–3.94) | - |  | 312 (0.86%) | 1.68 (1.50–1.87) | - |
| Q1 | 46 (0.51%) | 0.96 (0.72–1.29) | ref |  | 33 (0.36%) | 0.68 (0.49–0.96) | ref |
| Q2 | 139 (1.55%) | 3.00 (2.54–3.54) | **1.94 (1.38–2.73)** |  | 68 (0.75%) | 1.45 (1.14–1.83) | 1.44 (0.94–2.22) |
| Q3 | 172 (1.92%) | 3.78 (3.25–4.39) | **1.80 (1.27–2.54)** |  | 81 (0.89%) | 1.75 (1.41–2.18) | 1.35 (0.87–2.09) |
| Q4 | 313 (3.49%) | 7.15 (6.40–7.99) | **2.51 (1.77–3.57)** |  | 130 (1.44%) | 2.91 (2.45–3.46) | **1.72 (1.09–2.71)** |
| LVESV/LVSV |  |  |  |  |  |  |  |
| Total | 695 (1.88%) | 3.69 (3.42–3.97) | - |  | 320 (0.86%) | 1.67 (1.49–1.86) | - |
| Q1 | 154 (1.67%) | 3.29 (2.81–3.85) | 1.05 (0.84–1.32) |  | 66 (0.71%) | 1.39 (1.09–1.77) | ref |
| Q2 | 143 (1.55%) | 3.02 (2.56–3.56) | ref |  | 73 (0.78%) | 1.52 (1.21–1.91) | 1.15 (0.82–1.61) |
| Q3 | 161 (1.75%) | 3.40 (2.91–3.97) | 1.13 (0.90–1.42) |  | 83 (0.89%) | 1.73 (1.40–2.15) | 1.33 (0.96–1.85) |
| Q4 | 237 (2.57%) | 5.04 (4.44–5.72) | **1.52 (1.23–1.87)** |  | 98 (1.05%) | 2.05 (1.68–2.50) | **1.49 (1.08–2.06)** |

|  | **Incident HF** | | |  | **Incident CHD** | | |
| --- | --- | --- | --- | --- | --- | --- | --- |
|  | **Event, n (%)** | **Incidence event rate** | **Adj. HR (95% CI)** |  | **Event, n (%)** | **Incidence event rate** | **Adj. HR (95% CI)** |
| ASI/GLS |  |  |  |  |  |  |  |
| Total | 220 (0.66%) | 1.27 (1.11–1.44) | - |  | 537 (1.70%) | 3.26 (2.99–3.55) | - |
| Q1 | 43 (0.52%) | 1.01 (0.75–1.36) | 1.11 (0.72–1.71) |  | 115 (1.46%) | 2.85 (2.38–3.42) | ref |
| Q2 | 40 (0.48%) | 0.91 (0.67–1.25) | ref |  | 128 (1.62%) | 3.09 (2.60–3.68) | 1.05 (0.82–1.36) |
| Q3 | 55 (0.66%) | 1.24 (0.95–1.61) | 1.26 (0.84–1.90) |  | 151 (1.91%) | 3.59 (3.06–4.21) | 1.09 (0.85–1.40) |
| Q4 | 82 (0.99%) | 1.91 (1.54–2.37) | **1.66 (1.13–2.44)** |  | 143 (1.81%) | 3.51 (2.98–4.13) | 0.96 (0.74–1.23) |
| ePWV/GLS |  |  |  |  |  |  |  |
| Total | 230 (0.63%) | 1.22 (1.07–1.38) | - |  | 566 (1.62%) | 3.17 (2.92–3.44) | - |
| Q1 | 12 (0.13%) | 0.25 (0.14–0.43) | ref |  | 70 (0.80%) | 1.51 (1.20–1.91) | ref |
| Q2 | 26 (0.28%) | 0.55 (0.37–0.80) | 1.41 (0.70–2.83) |  | 109 (1.25%) | 2.42 (2.01–2.92) | 1.13 (0.83–1.54) |
| Q3 | 55 (0.60%) | 1.18 (0.90–1.53) | **2.32 (1.20–4.48)** |  | 168 (1.93%) | 3.79 (3.26–4.41) | **1.38 (1.01–1.87)** |
| Q4 | 137 (1.50%) | 3.04 (2.57–3.59) | **4.61 (2.39–8.87)** |  | 219 (2.51%) | 5.12 (4.48–5.84) | **1.48 (1.07–2.05)** |
| LVESV/LVSV |  |  |  |  |  |  |  |
| Total | 224 (0.59%) | 1.16 (1.01–1.31) | - |  | 593 (1.65%) | 3.23 (2.98–3.50) | - |
| Q1 | 36 (0.38%) | 0.75 (0.54–1.04) | ref |  | 143 (1.59%) | 3.14 (2.67–3.70) | 1.05 (0.83–1.33) |
| Q2 | 37 (0.39%) | 0.76 (0.55–1.05) | 1.15 (0.72–1.82) |  | 132 (1.47%) | 2.86 (2.42–3.40) | ref |
| Q3 | 32 (0.34%) | 0.66 (0.47–0.93) | 1.06 (0.66–1.72) |  | 130 (1.45%) | 2.82 (2.38–3.35) | 1.00 (0.78–1.27) |
| Q4 | 119 (1.26%) | 2.46 (2.06–2.95) | **3.75 (2.55–5.53)** |  | 188 (2.10%) | 4.10 (3.55–4.73) | **1.37 (1.09–1.72)** |

Incident event rates are unadjusted and expressed per 1,000 person-years. Adj. HR refers to the adjusted hazard ratio with 95% confidence intervals (CIs). Bold values indicate statistical significance (*P*-value < 0.05). Models were adjusted for age, sex, race, education, body mass index, smoking status, alcohol intake frequency, healthy physical activity, family history of heart disease, prevalent hypertension, dyslipidemia, diabetes, and CHD (prevalent CHD was excluded in the incident CHD model). Abbreviations: Adj., adjusted; AF, atrial fibrillation; ASI, arterial stiffness index; CHD, coronary heart disease; CI, confidence interval; ePWV, estimated pulse wave velocity; GLS, left ventricular global longitudinal strain; HF, heart failure; HR, hazard ratio; LVESV, left ventricular end-systolic volume; LVSV, left ventricular stroke volume; ref, reference; VAC, ventricular-arterial coupling.

## Table S7. Multivariable association of quartiles of VAC parameters with all-cause and CVD mortality.

|  | **All-Cause Mortality** | | |  | **CVD Mortality** | | |
| --- | --- | --- | --- | --- | --- | --- | --- |
|  | **Event, n (%)** | **Incidence event rate** | **Adj. HR (95% CI)** |  | **Event, n (%)** | **Incidence event rate** | **Adj. sHR (95% CI)** |
| ASI/GLS |  |  |  |  |  |  |  |
| Total | 618 (1.86%) | 3.54 (3.26–3.82) | - |  | 93 (0.28%) | 0.53 (0.43–0.65) | - |
| Q1 | 125 (1.50%) | 2.92 (2.45–3.48) | 1.06 (0.83–1.36) |  | 21 (0.25%) | 0.49 (0.32–0.75) | 1.74 (0.88–3.46) |
| Q2 | 123 (1.48%) | 2.79 (2.34–3.33) | ref |  | 13 (0.16%) | 0.30 (0.17–0.51) | ref |
| Q3 | 167 (2.01%) | 3.73 (3.21–4.34) | 1.18 (0.94–1.50) |  | 18 (0.22%) | 0.40 (0.25–0.64) | 1.22 (0.59–2.53) |
| Q4 | 203 (2.44%) | 4.70 (4.10–5.39) | **1.28 (1.02–1.61)** |  | 41 (0.49%) | 0.95 (0.70–1.29) | **2.44 (1.29–4.61)** |
| ePWV/GLS |  |  |  |  |  |  |  |
| Total | 659 (1.79%) | 3.48 (3.22–3.75) | - |  | 103 (0.28%) | 0.54 (0.44–0.65) | - |
| Q1 | 61 (0.66%) | 1.25 (0.97–1.60) | ref |  | 5 (0.05%) | 0.10 (0.04–0.25) | ref |
| Q2 | 112 (1.22%) | 2.34 (1.94–2.81) | 1.09 (0.79–1.50) |  | 17 (0.18%) | 0.35 (0.22–0.57) | 1.99 (0.72–5.55) |
| Q3 | 196 (2.13%) | 4.17 (3.62–4.79) | **1.37 (1.0007–1.87)** |  | 28 (0.30%) | 0.60 (0.41–0.86) | 2.35 (0.83–6.68) |
| Q4 | 290 (3.16%) | 6.36 (5.67–7.14) | **1.44 (1.04–1.99)** |  | 53 (0.58%) | 1.16 (0.89–1.52) | **3.16 (1.08–9.26)** |
| LVESV/LVSV |  |  |  |  |  |  |  |
| Total | 686 (1.81%) | 3.52 (3.26–3.79) | - |  | 106 (0.28%) | 0.54 (0.45–0.65) | - |
| Q1 | 135 (1.43%) | 2.79 (2.36–3.31) | ref |  | 12 (0.13%) | 0.25 (0.14–0.44) | ref |
| Q2 | 164 (1.73%) | 3.35 (2.88–3.90) | 1.22 (0.97–1.54) |  | 25 (0.26%) | 0.51 (0.35–0.76) | **2.13 (1.08–4.23)** |
| Q3 | 174 (1.84%) | 3.56 (3.07–4.13) | **1.29 (1.03–1.62)** |  | 20 (0.21%) | 0.41 (0.26–0.63) | 1.70 (0.82–3.53) |
| Q4 | 213 (2.25%) | 4.37 (3.82–5.00) | **1.40 (1.12–1.75)** |  | 49 (0.52%) | 1.01 (0.76–1.33) | **3.62 (1.89–6.90)** |

Incident event rates are unadjusted and expressed per 1,000 person-years. Adj. HR refers to the adjusted hazard ratio with 95% confidence intervals (CIs). Adj. sHR refers to the adjusted subdistribution hazard ratio estimated using Fine-Gray subdistribution hazard models to account for non-CVD death as a competing risk. Bold values indicate statistical significance (*P*-value < 0.05). Models were adjusted for age, sex, race, education, body mass index, smoking status, alcohol intake frequency, healthy physical activity, family history of heart disease, prevalent hypertension, dyslipidemia, diabetes, and CHD.

Abbreviations: Adj., adjusted; ASI, arterial stiffness index; CHD, coronary heart disease; CI, confidence interval; CVD, cardiovascular disease; ePWV, estimated pulse wave velocity; GLS, left ventricular global longitudinal strain; HR, hazard ratio; LVESV, left ventricular end-systolic volume; LVSV, left ventricular stroke volume; ref, reference; sHR, subdistribution hazard ratio; VAC, ventricular-arterial coupling.

## Table S8. Cause-specific associations of VAC parameters with CVD and non-CVD mortality.

|  | **CVD mortality** | | |  | **non-CVD mortality** | | |
| --- | --- | --- | --- | --- | --- | --- | --- |
|  | **Case / Total** | **Adj. HR (95% CI)** | ***P*-value** |  | **Case / Total** | **Adj. HR (95% CI)** | ***P*-value** |
| Age + Sex + ASI/GLS | 93 / 33,314 | 1.35 (1.14–1.60) | 0.001 |  | 525 / 33,314 | 1.10 (1.02–1.19) | 0.020 |
| Clinical features + ASI/GLS | 93 / 33,314 | 1.32 (1.11–1.57) | 0.001 |  | 525 / 33,314 | 1.08 (0.99–1.17) | 0.074 |
| Clinical features + LVMI + ASI/GLS | 88 / 32,659 | 1.31 (1.10–1.56) | 0.002 |  | 503 / 32,659 | 1.08 (0.99–1.17) | 0.070 |
| Clinical features + LVMI + LVEF + ASI/GLS | 88 / 32,659 | 1.15 (0.95–1.38) | 0.148 |  | 503 / 32,659 | 1.07 (0.98–1.16) | 0.147 |
|  |  |  |  |  |  |  |  |
| Age + Sex + ePWV/GLS | 103 / 36,761 | 1.47 (1.24–1.75) | <0.001 |  | 556 / 36,761 | 1.13 (1.03–1.23) | 0.010 |
| Clinical features + ePWV/GLS | 103 / 36,761 | 1.41 (1.19–1.68) | <0.001 |  | 556 / 36,761 | 1.11 (1.02–1.22) | 0.021 |
| Clinical features + LVMI + ePWV/GLS | 97 / 36,048 | 1.31 (1.08–1.58) | 0.005 |  | 533 / 36,048 | 1.13 (1.03–1.24) | 0.010 |
| Clinical features + LVMI + LVEF + ePWV/GLS | 97 / 36,048 | 1.08 (0.87–1.34) | 0.496 |  | 533 / 36,048 | 1.12 (1.01–1.23) | 0.033 |
|  |  |  |  |  |  |  |  |
| Age + Sex + LVESV/LVSV | 106 / 37,841 | 1.53 (1.33–1.76) | <0.001 |  | 580 / 37,841 | 1.07 (0.99–1.15) | 0.092 |
| Clinical features + LVESV/LVSV | 106 / 37,841 | 1.51 (1.31–1.74) | <0.001 |  | 580 / 37,841 | 1.06 (0.98–1.14) | 0.145 |
| Clinical features + LVMI + LVESV/LVSV | 101 / 37,191 | 1.48 (1.28–1.71) | <0.001 |  | 563 / 37,191 | 1.07 (0.99–1.16) | 0.077 |

Clinical features included age, sex, race, education, body mass index, smoking status, alcohol intake frequency, healthy physical activity, family history of heart disease, prevalent hypertension, dyslipidemia, diabetes, and CHD. Multivariable cause-specific Cox proportional hazards models were used for both CVD and non-CVD mortality, with competing events censored. Abbreviations: ASI/GLS, ratio of arterial stiffness index to global longitudinal strain; CHD, coronary heart disease; CI, confidence interval; CVD, cardiovascular disease; ePWV/GLS, ratio of estimated pulse wave velocity to global longitudinal strain; HR, hazard ratio; LVEF, left ventricular ejection fraction; LVESV/LVSV, ratio of left ventricular end-systolic volume to stroke volume; LVMI, left ventricular mass index; VAC, ventricular-arterial coupling.

## Table S9. E-values for unmeasured confounding assessment across multivariable models.

| **Incident AF** | **HR (95% CI)** | **E-values (Lower, Upper)** |
| --- | --- | --- |
| Age + Sex + ASI/GLS | 1.16 (1.08–1.25) | 1.59 (1.37, NA) |
| Clinical features + ASI/GLS | 1.15 (1.07–1.24) | 1.57 (1.34, NA) |
| Clinical features + LAVmax-I + LAEF + ASI/GLS | 1.05 (0.98–1.13) | 1.28 (1.00, NA) |
| Age + Sex + ePWV/GLS | 1.44 (1.34–1.55) | 2.24 (2.01, NA) |
| Clinical features + ePWV/GLS | 1.41 (1.31–1.52) | 2.17 (1.95, NA) |
| Clinical features + LAVmax-I + LAEF + ePWV/GLS | 1.19 (1.10–1.29) | 1.67 (1.43, NA) |
| Age + Sex + LVESV/LVSV | 1.24 (1.16–1.32) | 1.79 (1.59, NA) |
| Clinical features + LVESV/LVSV | 1.24 (1.16–1.32) | 1.79 (1.59, NA) |
| Clinical features + LAVmax-I + LAEF + LVESV/LVSV | 1.06 (0.99–1.14) | 1.31 (1.00, NA) |

| **Incident Stroke** | **HR (95% CI)** | **E-values (Lower, Upper)** |
| --- | --- | --- |
| Age + Sex + ASI/GLS | 1.19 (1.07–1.32) | 1.67 (1.34, NA) |
| Clinical features + ASI/GLS | 1.18 (1.06–1.32) | 1.64 (1.31, NA) |
| Clinical features + LAVmax-I + ASI/GLS | 1.18 (1.06–1.31) | 1.64 (1.31, NA) |
| Age + Sex + ePWV/GLS | 1.28 (1.14–1.43) | 1.88 (1.54, NA) |
| Clinical features + ePWV/GLS | 1.24 (1.11–1.39) | 1.79 (1.46, NA) |
| Clinical features + LAVmax-I + ePWV/GLS | 1.22 (1.09–1.37) | 1.74 (1.40, NA) |
| Age + Sex + LVESV/LVSV | 1.17 (1.05–1.29) | 1.62 (1.28, NA) |
| Clinical features + LVESV/LVSV | 1.16 (1.05–1.28) | 1.59 (1.28, NA) |
| Clinical features + LAVmax-I + LVESV/LVSV | 1.13 (1.02–1.26) | 1.51 (1.16, NA) |

| **Incident HF** | **HR (95% CI)** | **E-values (Lower, Upper)** |
| --- | --- | --- |
| Age + Sex + ASI/GLS | 1.31 (1.17–1.47) | 1.95 (1.62, NA) |
| Clinical features + ASI/GLS | 1.27 (1.14–1.43) | 1.86 (1.54, NA) |
| Clinical features + LVGFI + ASI/GLS | 0.96 (0.85–1.08) | 1.25 (NA, 1.00) |
| Clinical features + LVEDVI + LVEF + ASI/GLS | 1.00 (0.88–1.14) | 1.00 (1.00, NA) |
| Age + Sex + ePWV/GLS | 1.75 (1.57–1.94) | 2.90 (2.52, NA) |
| Clinical features + ePWV/GLS | 1.67 (1.51–1.85) | 2.73 (2.39, NA) |
| Clinical features + LVGFI + ePWV/GLS | 1.26 (1.11–1.43) | 1.83 (1.46, NA) |
| Clinical features + LVEDVI + LVEF + ePWV/GLS | 1.14 (0.99–1.31) | 1.54 (1.00, NA) |
| Age + Sex + LVESV/LVSV | 1.82 (1.67–1.99) | 3.04 (2.73, NA) |
| Clinical features + LVESV/LVSV | 1.76 (1.61–1.91) | 2.92 (2.60, NA) |

| **Incident CHD** | **HR (95% CI)** | **E-values (Lower, Upper)** |
| --- | --- | --- |
| Age + Sex + ASI/GLS | 1.03 (0.95–1.12) | 1.21 (1.00, NA) |
| Clinical features + ASI/GLS | 1.00 (0.92–1.09) | 1.00 (1.00, NA) |
| Clinical features + LVESVI + ASI/GLS | 1.00 (0.92–1.09) | 1.00 (1.00, NA) |
| Clinical features + LVEDVI + LVEF + ASI/GLS | 0.99 (0.91–1.08) | 1.11 (NA, 1.00) |
| Age + Sex + ePWV/GLS | 1.17 (1.07–1.28) | 1.62 (1.34, NA) |
| Clinical features + ePWV/GLS | 1.13 (1.03–1.24) | 1.51 (1.21, NA) |
| Clinical features + LVESVI + ePWV/GLS | 1.12 (1.01–1.23) | 1.49 (1.11, NA) |
| Clinical features + LVEDVI + LVEF + ePWV/GLS | 1.11 (1.00–1.23) | 1.46 (1.00, NA) |
| Age + Sex + LVESV/LVSV | 1.13 (1.05–1.22) | 1.51 (1.28, NA) |
| Clinical features + LVESV/LVSV | 1.14 (1.06–1.23) | 1.54 (1.31, NA) |

| **All-Cause Mortality** | **HR (95% CI)** | **E-values (Lower, Upper)** |
| --- | --- | --- |
| Age + Sex + ASI/GLS | 1.14 (1.06–1.22) | 1.54 (1.31, NA) |
| Clinical features + ASI/GLS | 1.11 (1.04–1.20) | 1.46 (1.24, NA) |
| Clinical features + LVMI + ASI/GLS | 1.12 (1.04–1.20) | 1.49 (1.24, NA) |
| Clinical features + LVMI + LVEF + ASI/GLS | 1.08 (1.00–1.17) | 1.37 (1.00, NA) |
| Age + Sex + ePWV/GLS | 1.18 (1.09–1.28) | 1.64 (1.40, NA) |
| Clinical features + ePWV/GLS | 1.16 (1.07–1.26) | 1.59 (1.34, NA) |
| Clinical features + LVMI + ePWV/GLS | 1.16 (1.07–1.26) | 1.59 (1.34, NA) |
| Clinical features + LVMI + LVEF + ePWV/GLS | 1.12 (1.02–1.22) | 1.49 (1.16, NA) |
| Age + Sex + LVESV/LVSV | 1.15 (1.07–1.23) | 1.57 (1.34, NA) |
| Clinical features + LVESV/LVSV | 1.13 (1.06–1.21) | 1.51 (1.31, NA) |
| Clinical features + LVMI + LVESV/LVSV | 1.14 (1.06–1.22) | 1.54 (1.31, NA) |

| **CVD Mortality** | **HR (95% CI)** | **E-values (Lower, Upper)** |
| --- | --- | --- |
| Age + Sex + ASI/GLS | 1.35 (1.11–1.63) | 2.04 (1.46, NA) |
| Clinical features + ASI/GLS | 1.32 (1.09–1.60) | 1.97 (1.40, NA) |
| Clinical features + LVMI + ASI/GLS | 1.30 (1.08–1.58) | 1.92 (1.37, NA) |
| Clinical features + LVMI + LVEF + ASI/GLS | 1.14 (0.94–1.39) | 1.54 (1.00, NA) |
| Age + Sex + ePWV/GLS | 1.47 (1.22–1.76) | 2.30 (1.74, NA) |
| Clinical features + ePWV/GLS | 1.41 (1.18–1.69) | 2.17 (1.64, NA) |
| Clinical features + LVMI + ePWV/GLS | 1.31 (1.08–1.59) | 1.95 (1.37, NA) |
| Clinical features + LVMI + LVEF + ePWV/GLS | 1.08 (0.86–1.35) | 1.37 (1.00, NA) |
| Age + Sex + LVESV/LVSV | 1.53 (1.33–1.76) | 2.43 (1.99, NA) |
| Clinical features + LVESV/LVSV | 1.51 (1.32–1.73) | 2.39 (1.97, NA) |
| Clinical features + LVMI + LVESV/LVSV | 1.47 (1.28–1.70) | 2.30 (1.88, NA) |

E-values quantify the minimum strength of association that an unmeasured confounder (or selection bias) would need to have with both the exposure and the outcome to explain away the observed association. Values are presented for the point estimate, followed by the E-values for the 95% confidence interval limits (Lower, Upper).

Abbreviations: AF, atrial fibrillation; ASI, arterial stiffness index; CHD, coronary heart disease; CI, confidence interval; CVD, cardiovascular disease; ePWV, estimated pulse wave velocity; GLS, left ventricular global longitudinal strain; HF, heart failure; HR, hazard ratio; LAEF, left atrial emptying fraction; LAVmax-I, maximal left atrial volume index; LVEDVI, left ventricular end-diastolic volume index; LVEF, left ventricular ejection fraction; LVESV, left ventricular end-systolic volume; LVESVI, left ventricular end-systolic volume index; LVGFI, left ventricular global function index; LVMI, left ventricular mass index; LVSV, left ventricular stroke volume; NA, not applicable; VAC, ventricular-arterial coupling.

## Figure S1. RCS analysis of the association between VAC parameters and incident AF.


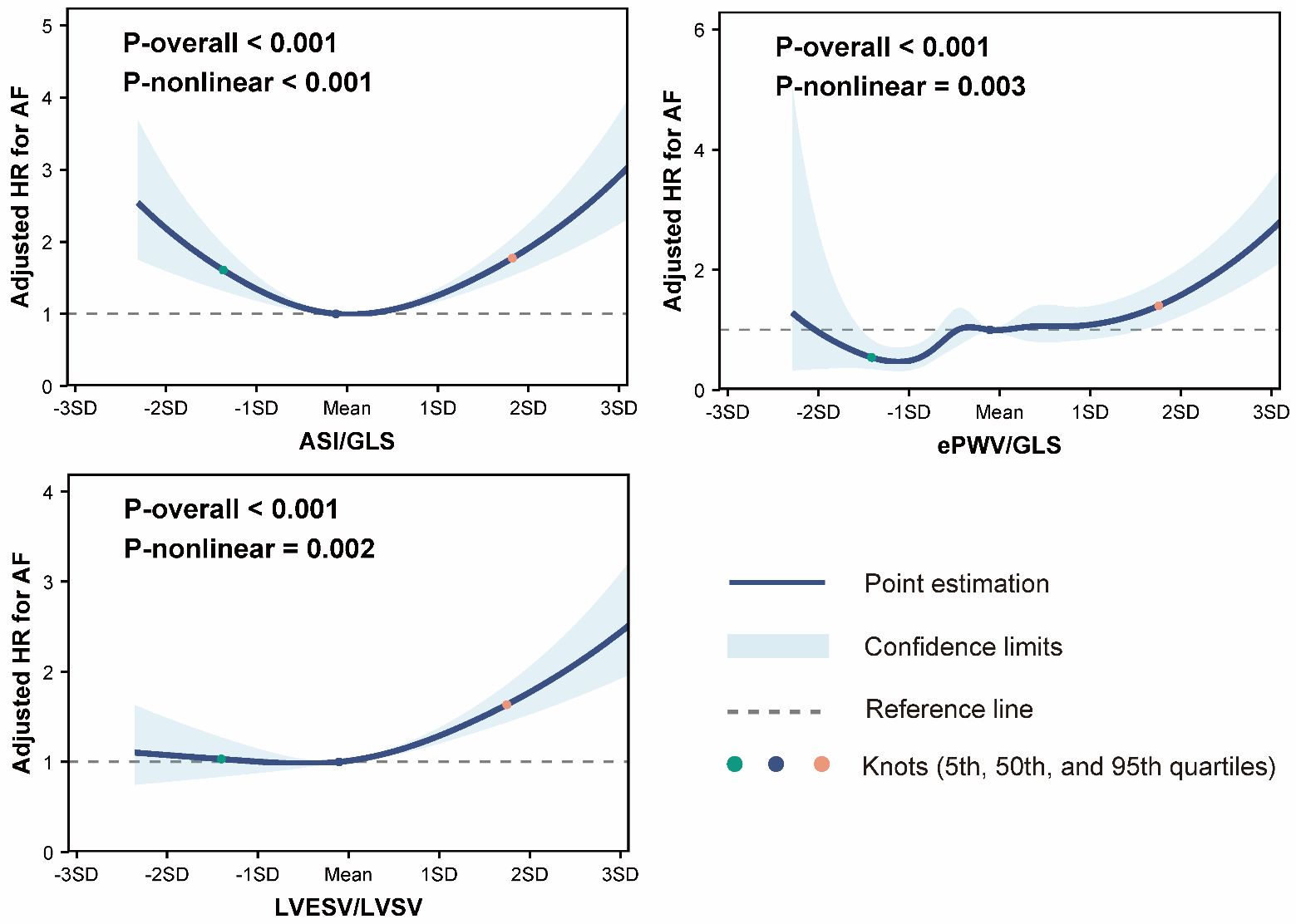


The solid blue lines represent the multivariable-adjusted hazard ratios, and the shaded areas represent the 95% confidence intervals. The dashed line indicates a hazard ratio of 1.00, with the median value used as the reference. Knots were placed at the 5th, 50th, and 95th percentiles to visualize the distribution across the data range.

The models were adjusted for age, sex, race, education, body mass index, smoking status, alcohol intake frequency, healthy physical activity, family history of heart disease, prevalent hypertension, dyslipidemia, diabetes, and coronary heart disease.

Abbreviations: AF, atrial fibrillation; ASI, arterial stiffness index; ePWV, estimated pulse wave velocity; GLS, left ventricular global longitudinal strain; HR, hazard ratio; LVESV, left ventricular end-systolic volume; LVSV, left ventricular stroke volume; RCS, restricted cubic spline; VAC, ventricular-arterial coupling.

## Figure S2. Cumulative incidence of AF according to quartiles of VAC parameters.


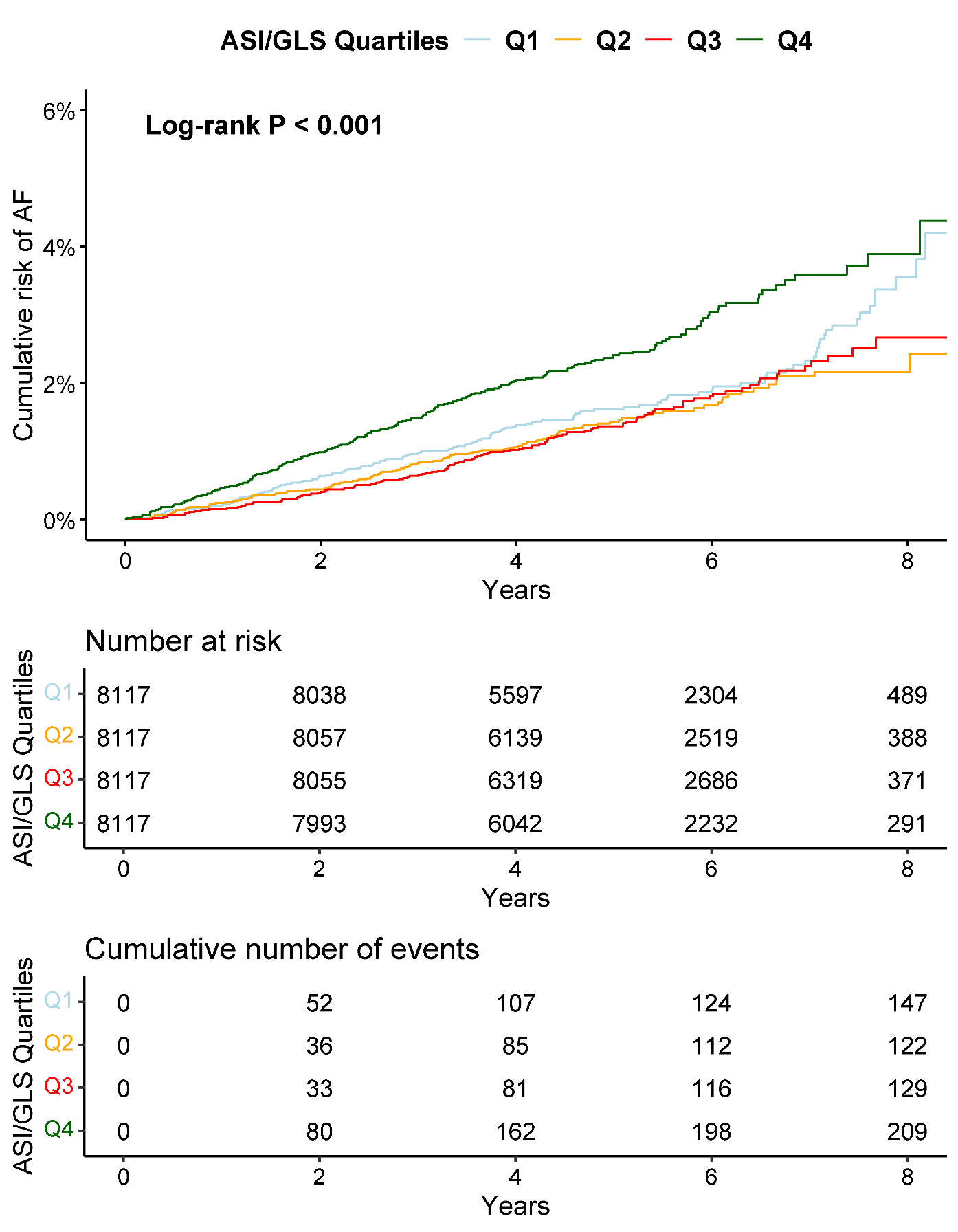
Univariable survival analysis showed the association between quartiles of ASI/GLS and the risk of AF, evaluated using the Kaplan-Meier method and compared using the Log-rank test. Abbreviations: AF, atrial fibrillation; ASI, arterial stiffness index; GLS, left ventricular global longitudinal strain; VAC, ventricular-arterial coupling.


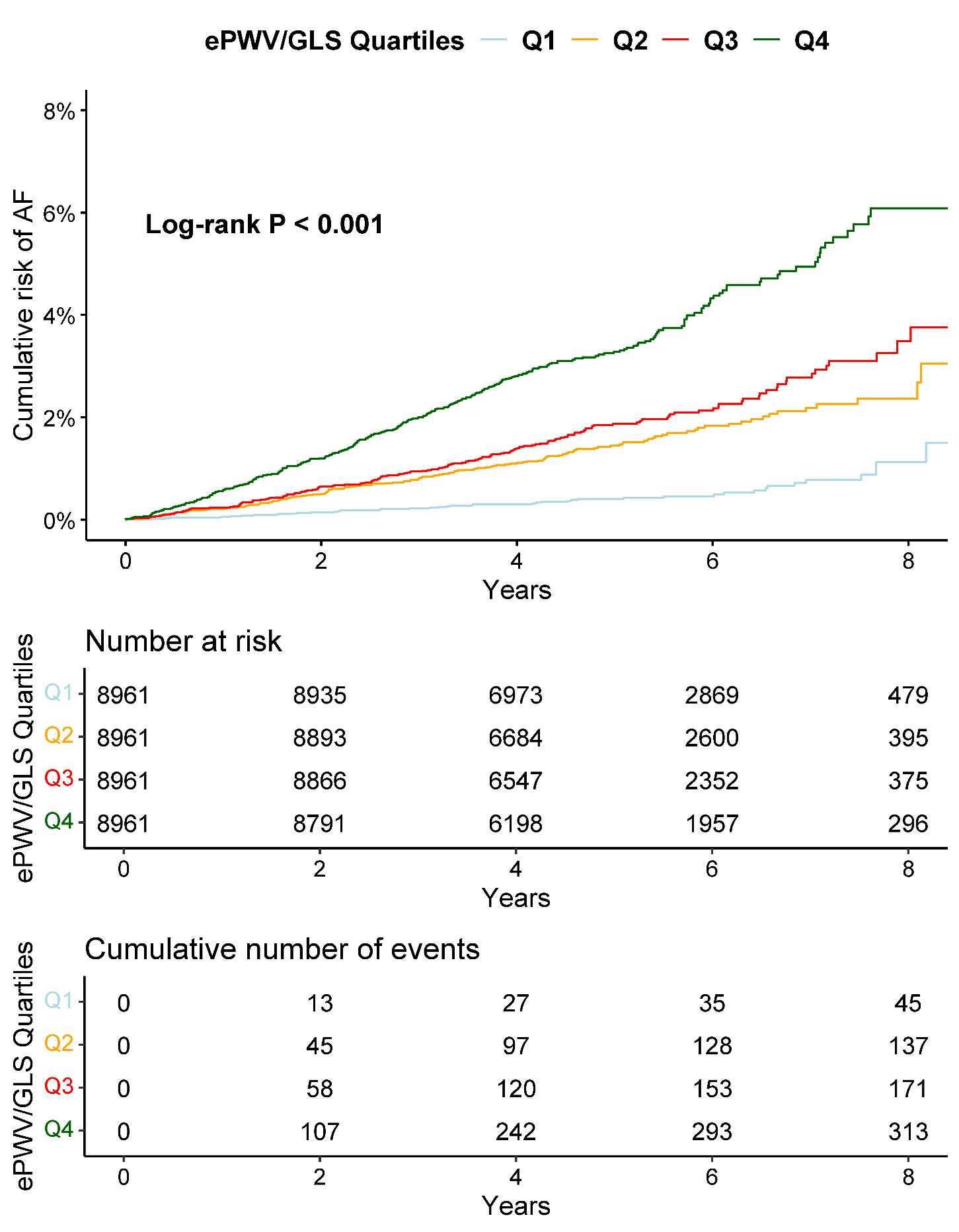


Univariable survival analysis showed the association between quartiles of ePWV/GLS and the risk of AF, evaluated using the Kaplan-Meier method and compared using the Log-rank test. Abbreviations: AF, atrial fibrillation; ePWV, estimated pulse wave velocity; GLS, left ventricular global longitudinal strain; VAC, ventricular-arterial coupling.


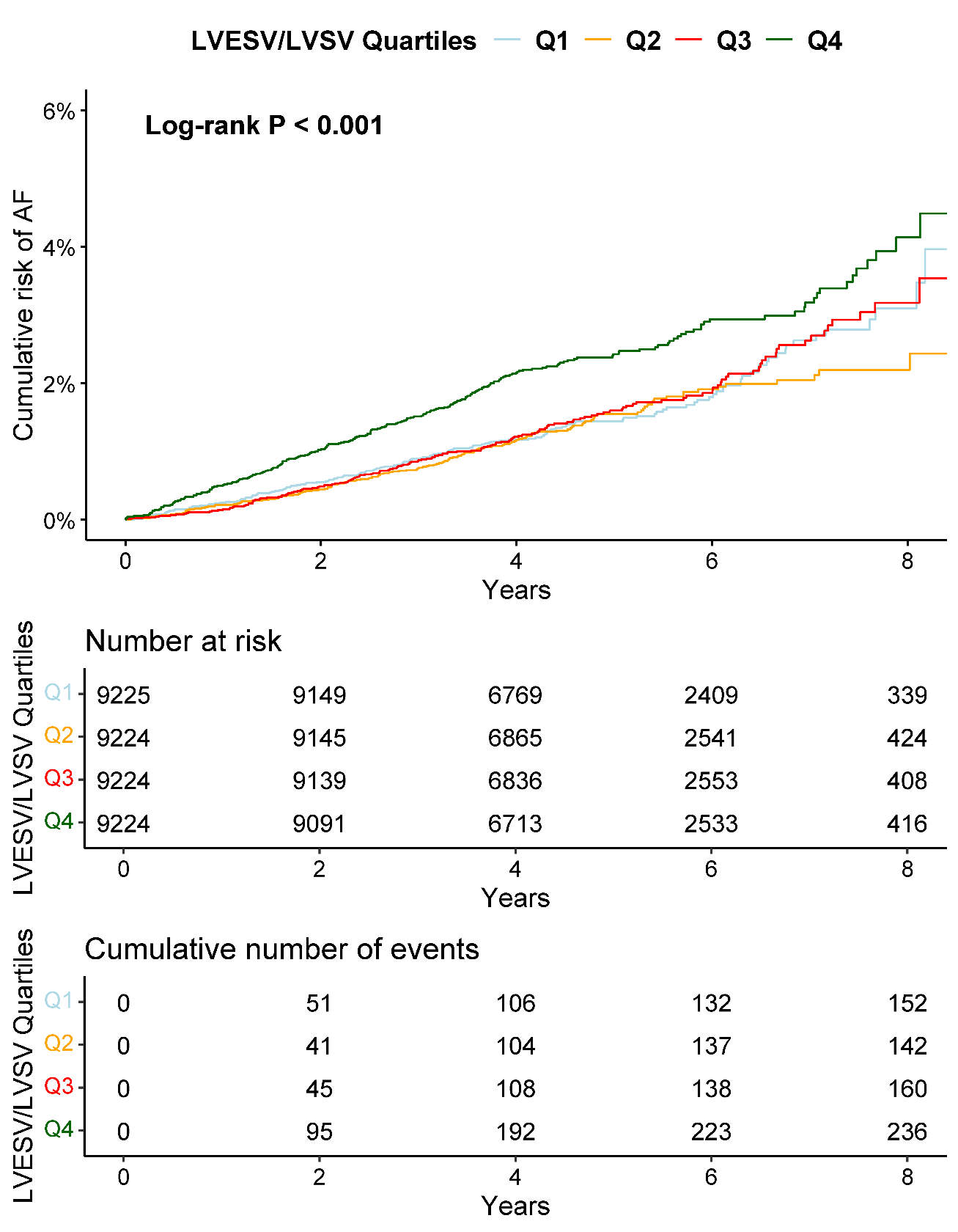


Univariable survival analysis showed the association between quartiles of LVESV/LVSV and the risk of AF, evaluated using the Kaplan-Meier method and compared using the Log-rank test. Abbreviations: AF, atrial fibrillation; LVESV, left ventricular end-systolic volume; LVSV, left ventricular stroke volume; VAC, ventricular-arterial coupling.

## Figure S3. Incremental value of VAC parameters over its constituent components for incident AF.


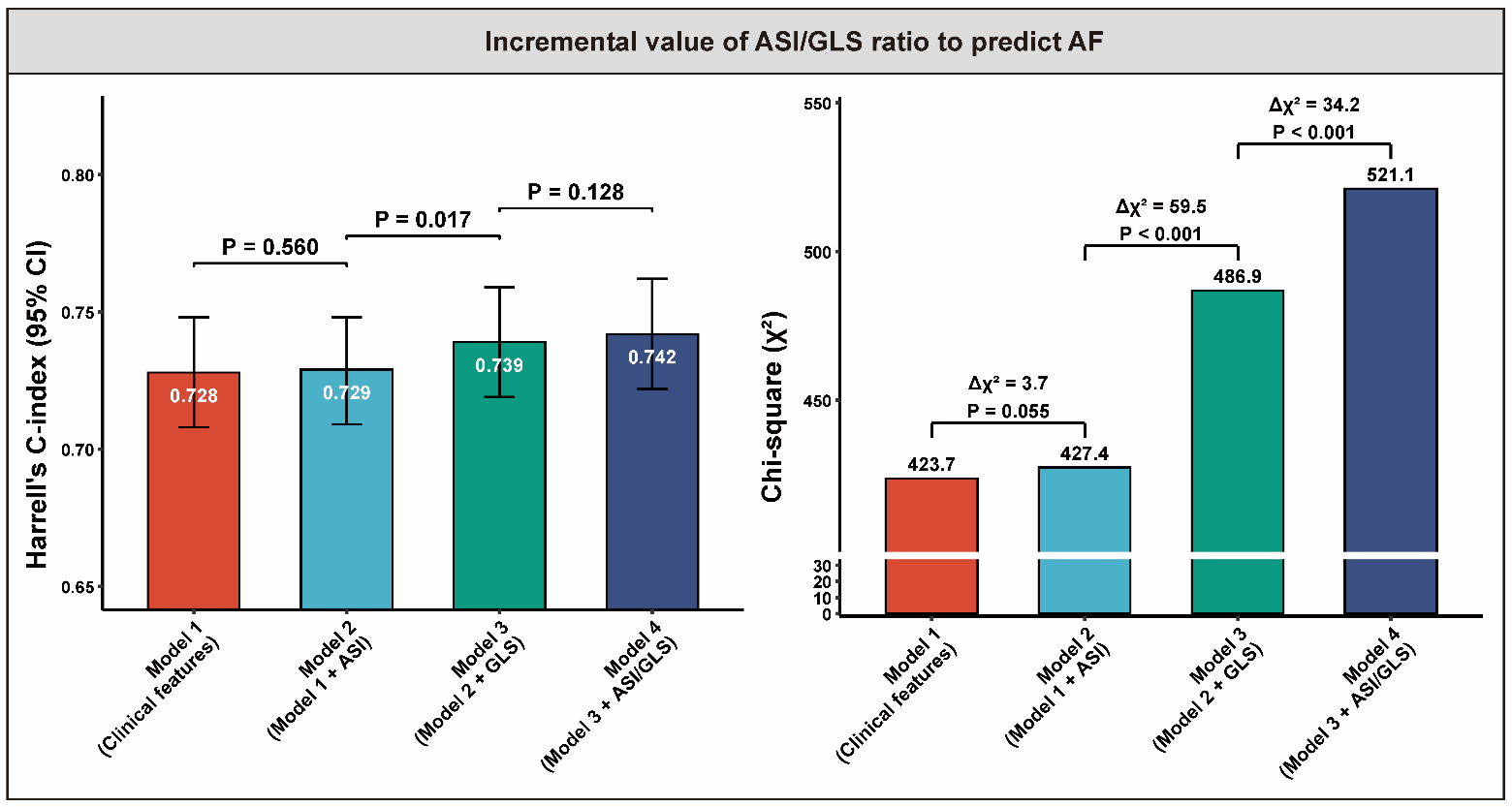


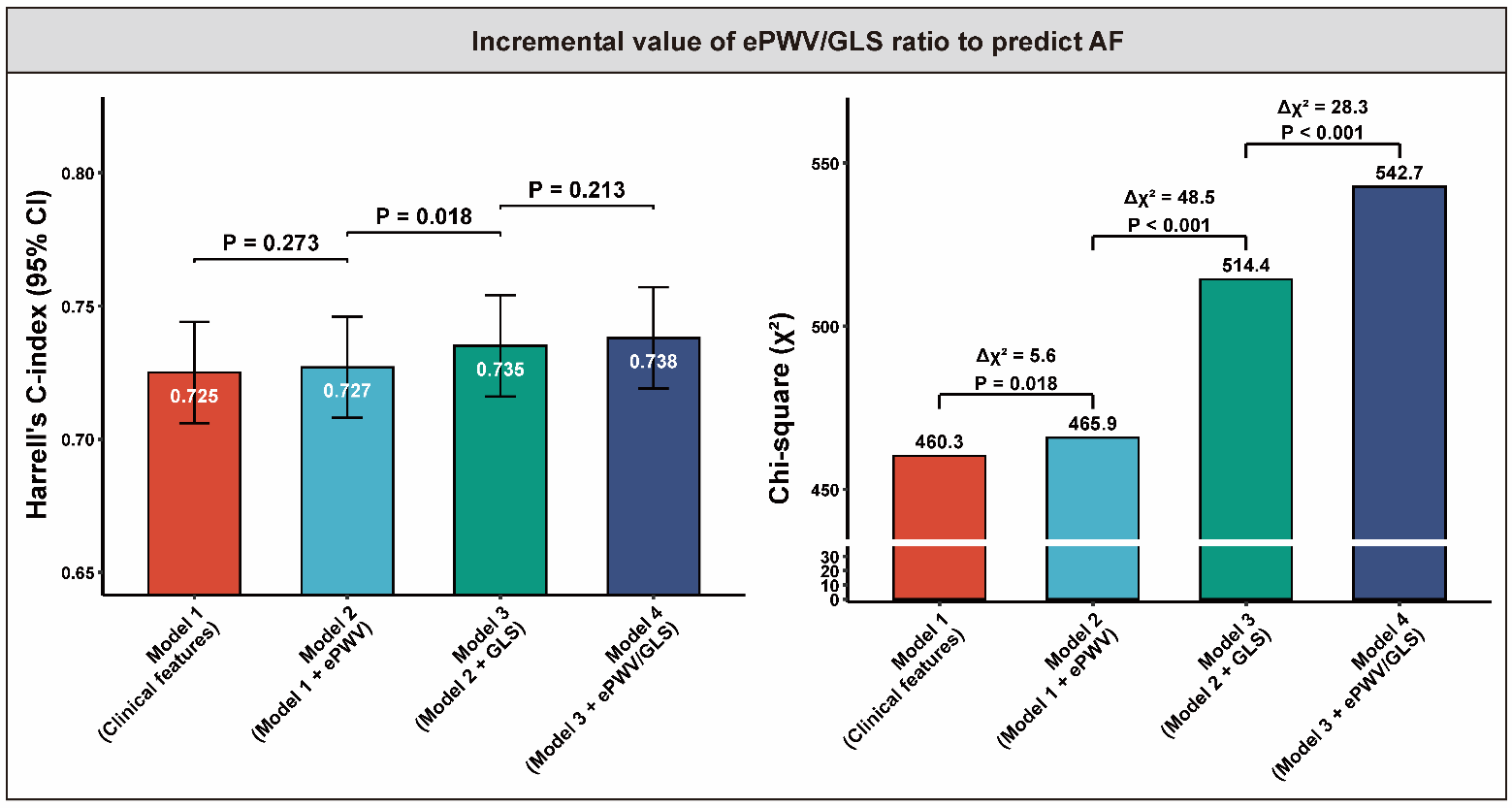


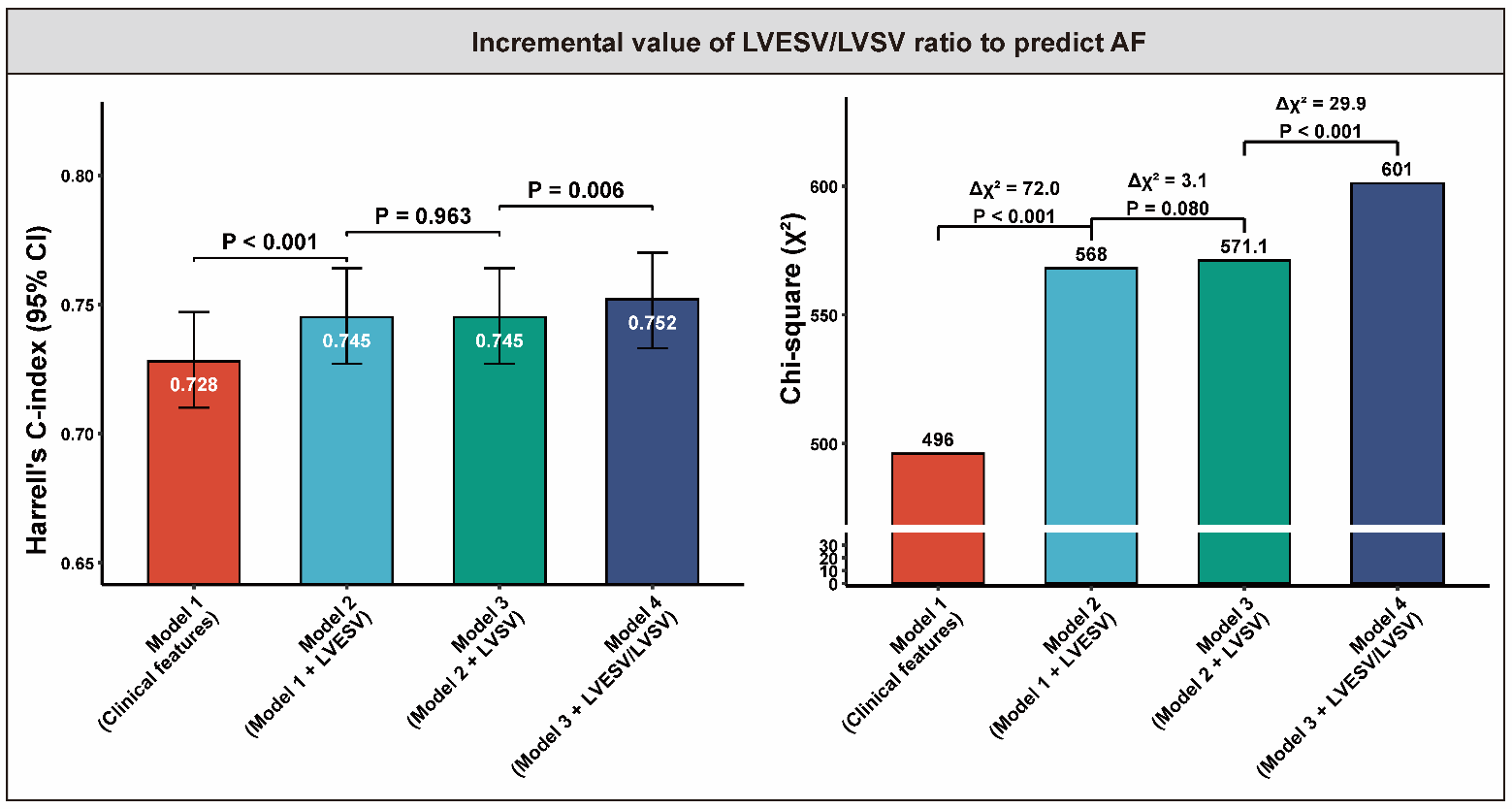


Incremental predictive value of ASI/GLS, ePWV/GLS, and LVESV/LVSV for predicting incident AF. The left panels display Harrell's C-indices, and the right panels display the likelihood ratio test chi-square (χ²) statistics. Clinical features included age, sex, race, education, body mass index, smoking status, alcohol intake frequency, healthy physical activity, family history of heart disease, prevalent hypertension, dyslipidemia, diabetes, and coronary heart disease.

Model 1 included clinical features only. Model 2 added the numerator component (ASI, ePWV, or LVESV) to Model 1. Model 3 added the denominator component (GLS or LVSV) to Model 2 (representing the model with both constituent components). Model 4 added the VAC parameter (ratio) to Model 3. *P*-values above brackets indicate the statistical significance of the comparison between the indicated models. Δχ² indicates the change in chi-square statistic. Abbreviations: AF, atrial fibrillation; ASI/GLS, ratio of arterial stiffness index to global longitudinal strain; ePWV/GLS, ratio of estimated pulse wave velocity to global longitudinal strain; LVESV/LVSV, ratio of left ventricular end-systolic volume to stroke volume; VAC, ventricular-arterial coupling.

## Figure S4. RCS analysis of the association between VAC parameters and incident stroke.


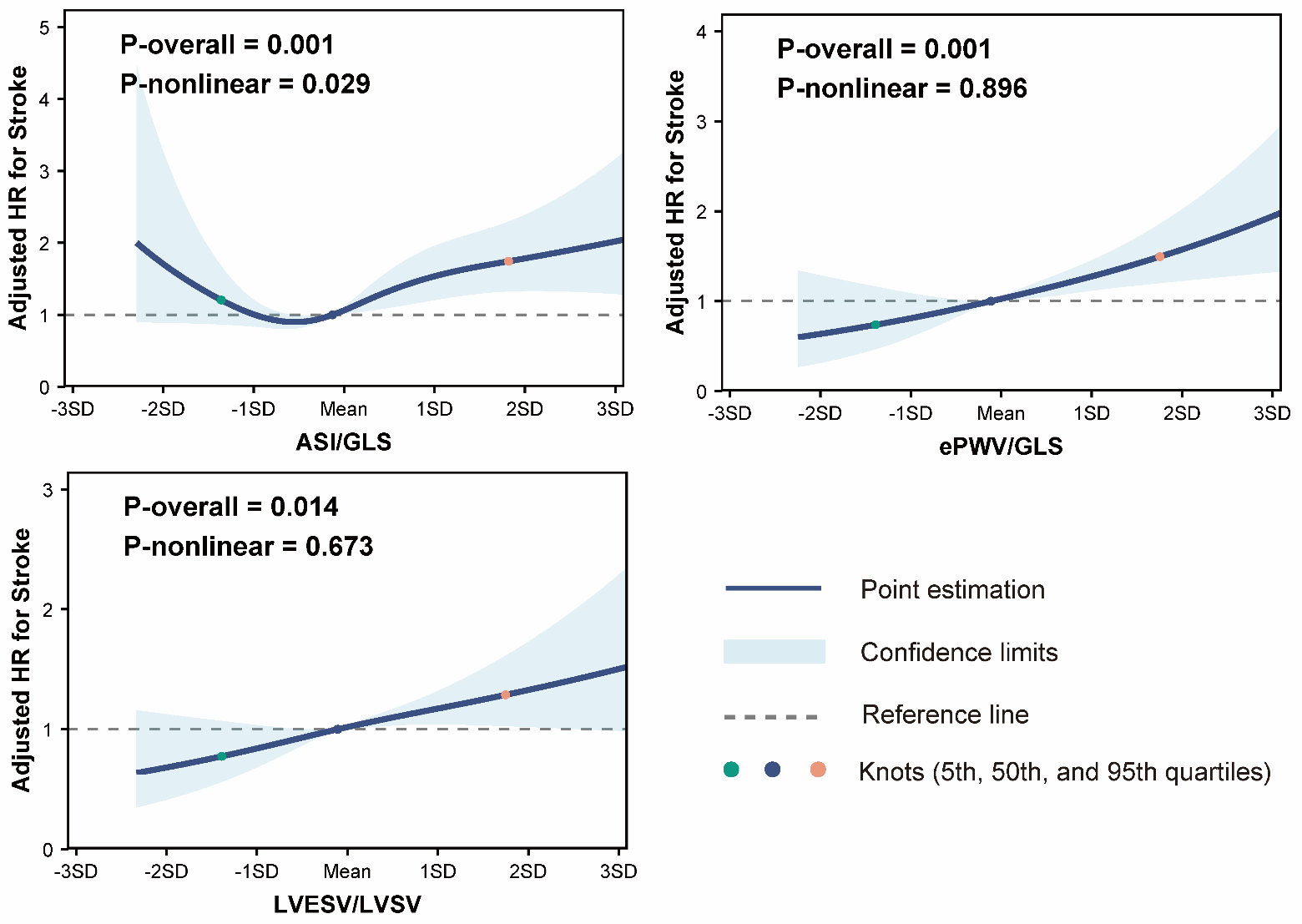


The solid blue lines represent the multivariable-adjusted hazard ratios, and the shaded areas represent the 95% confidence intervals. The dashed line indicates a hazard ratio of 1.00, with the median value used as the reference. Knots were placed at the 5th, 50th, and 95th percentiles to visualize the distribution across the data range.

The models were adjusted for age, sex, race, education, body mass index, smoking status, alcohol intake frequency, healthy physical activity, family history of heart disease, prevalent hypertension, dyslipidemia, diabetes, and coronary heart disease.

Abbreviations: ASI, arterial stiffness index; ePWV, estimated pulse wave velocity; GLS, left ventricular global longitudinal strain; HR, hazard ratio; LVESV, left ventricular end-systolic volume; LVSV, left ventricular stroke volume; RCS, restricted cubic spline; VAC, ventricular-arterial coupling.

## Figure S5. Cumulative incidence of stroke according to quartiles of VAC parameters.


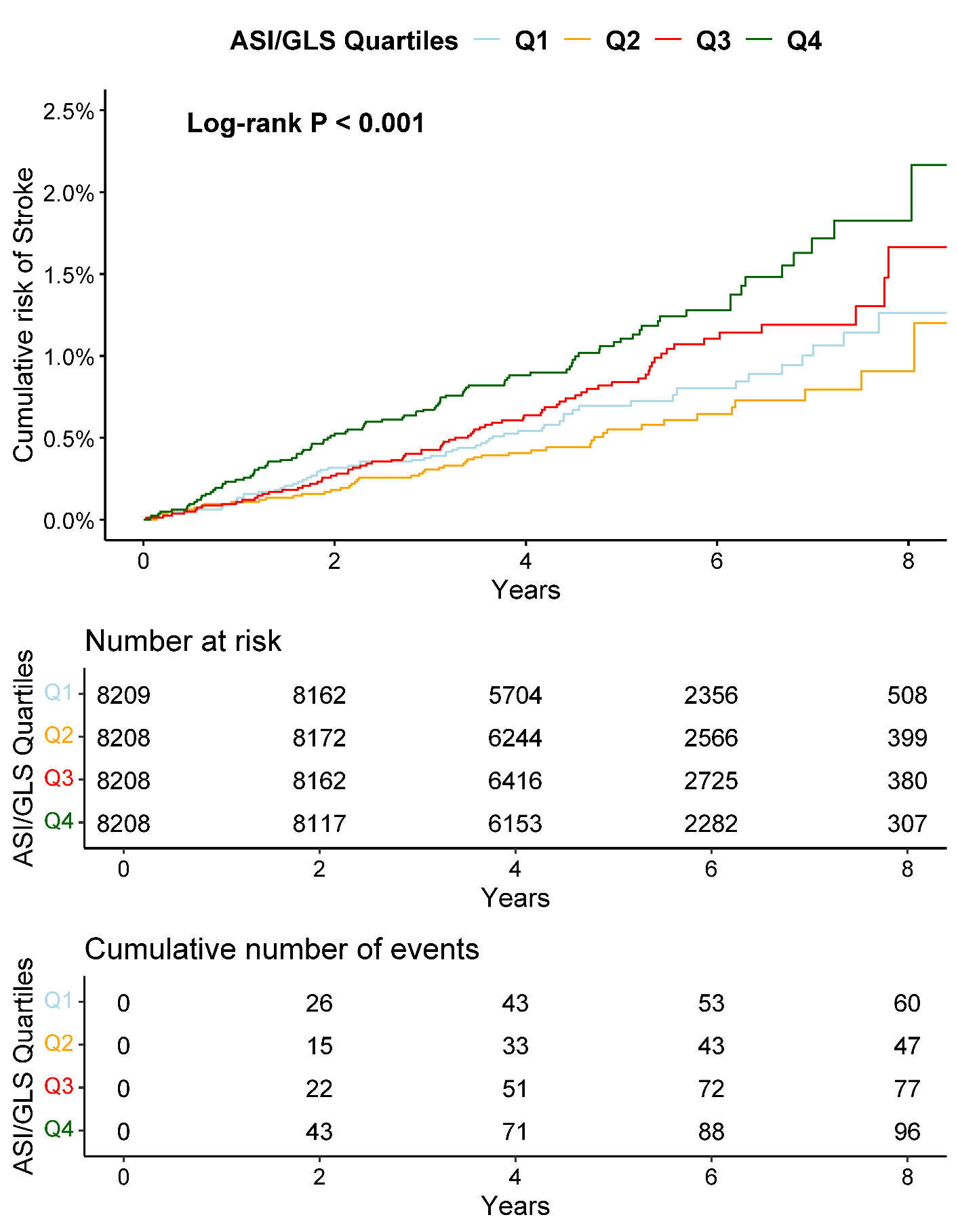
 Univariable survival analysis showed the association between quartiles of ASI/GLS and the risk of stroke, evaluated using the Kaplan-Meier method and compared using the Log-rank test. Abbreviations: ASI, arterial stiffness index; GLS, left ventricular global longitudinal strain; VAC, ventricular-arterial coupling.


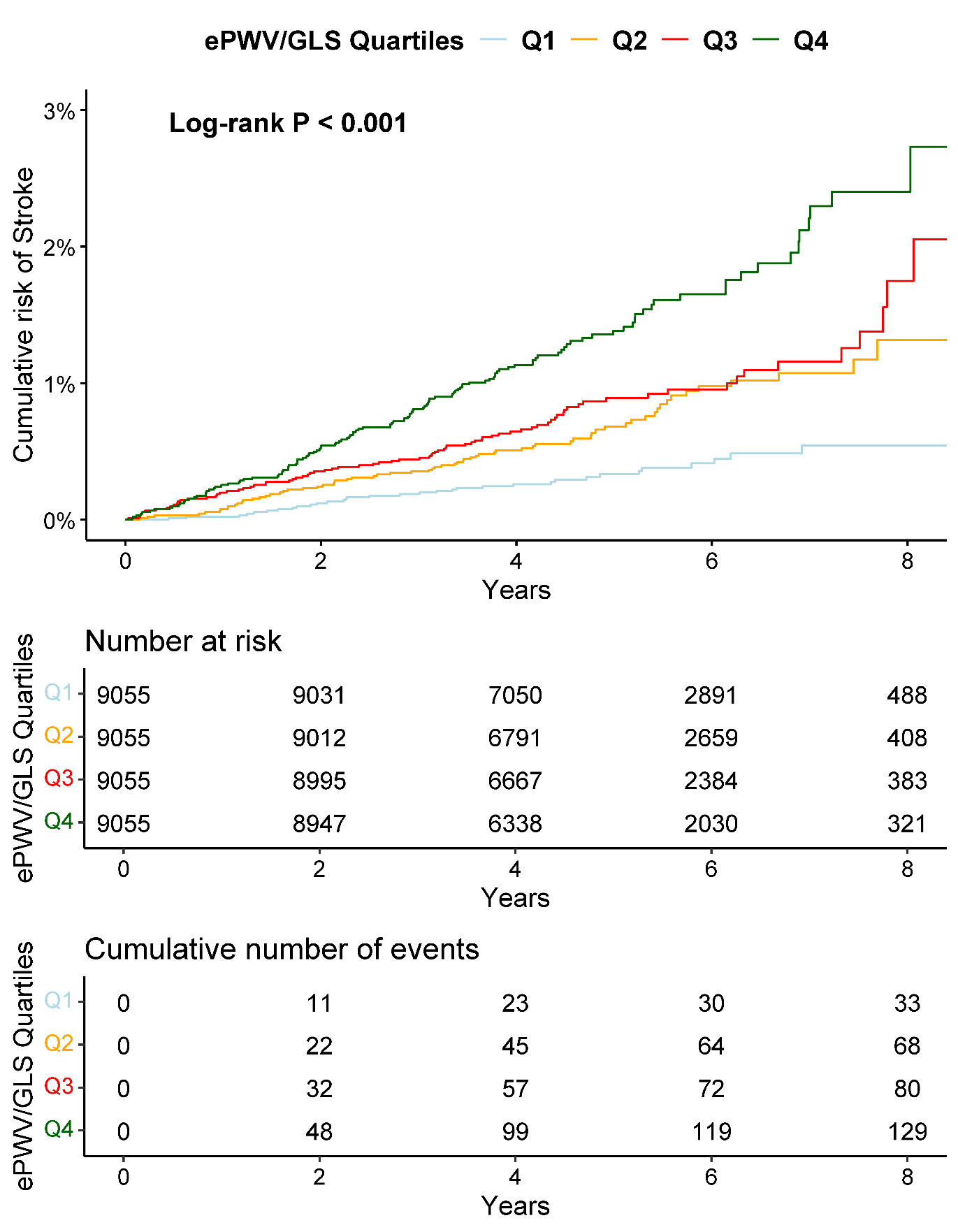


Univariable survival analysis showed the association between quartiles of ePWV/GLS and the risk of stroke, evaluated using the Kaplan-Meier method and compared using the Log-rank test. Abbreviations: ePWV, estimated pulse wave velocity; GLS, left ventricular global longitudinal strain; VAC, ventricular-arterial coupling.


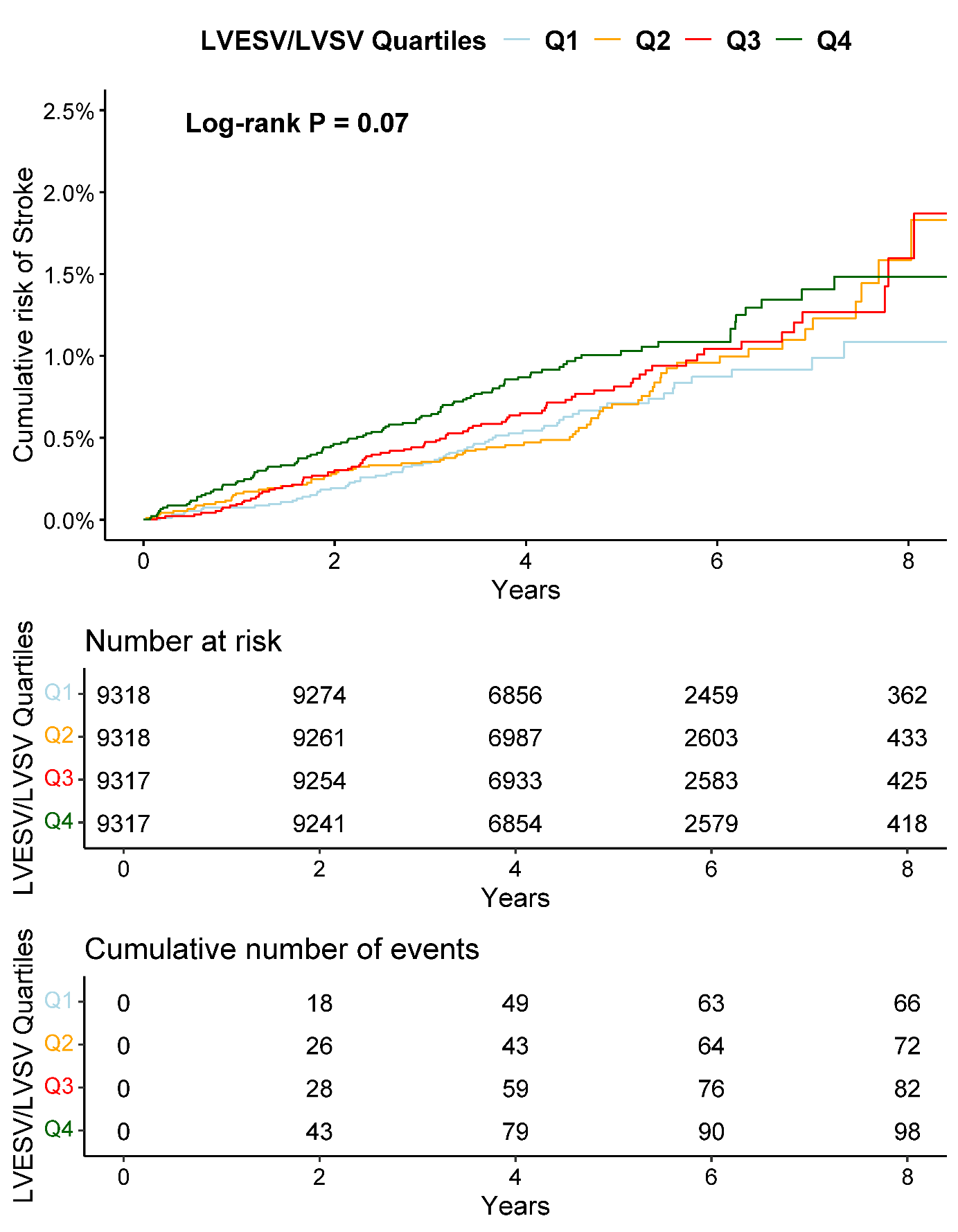


Univariable survival analysis showed the association between quartiles of LVESV/LVSV and the risk of stroke, evaluated using the Kaplan-Meier method and compared using the Log-rank test. Abbreviations: LVESV, left ventricular end-systolic volume; LVSV, left ventricular stroke volume; VAC, ventricular-arterial coupling.

## Figure S6. Incremental value of VAC parameters over its constituent components for incident stroke.


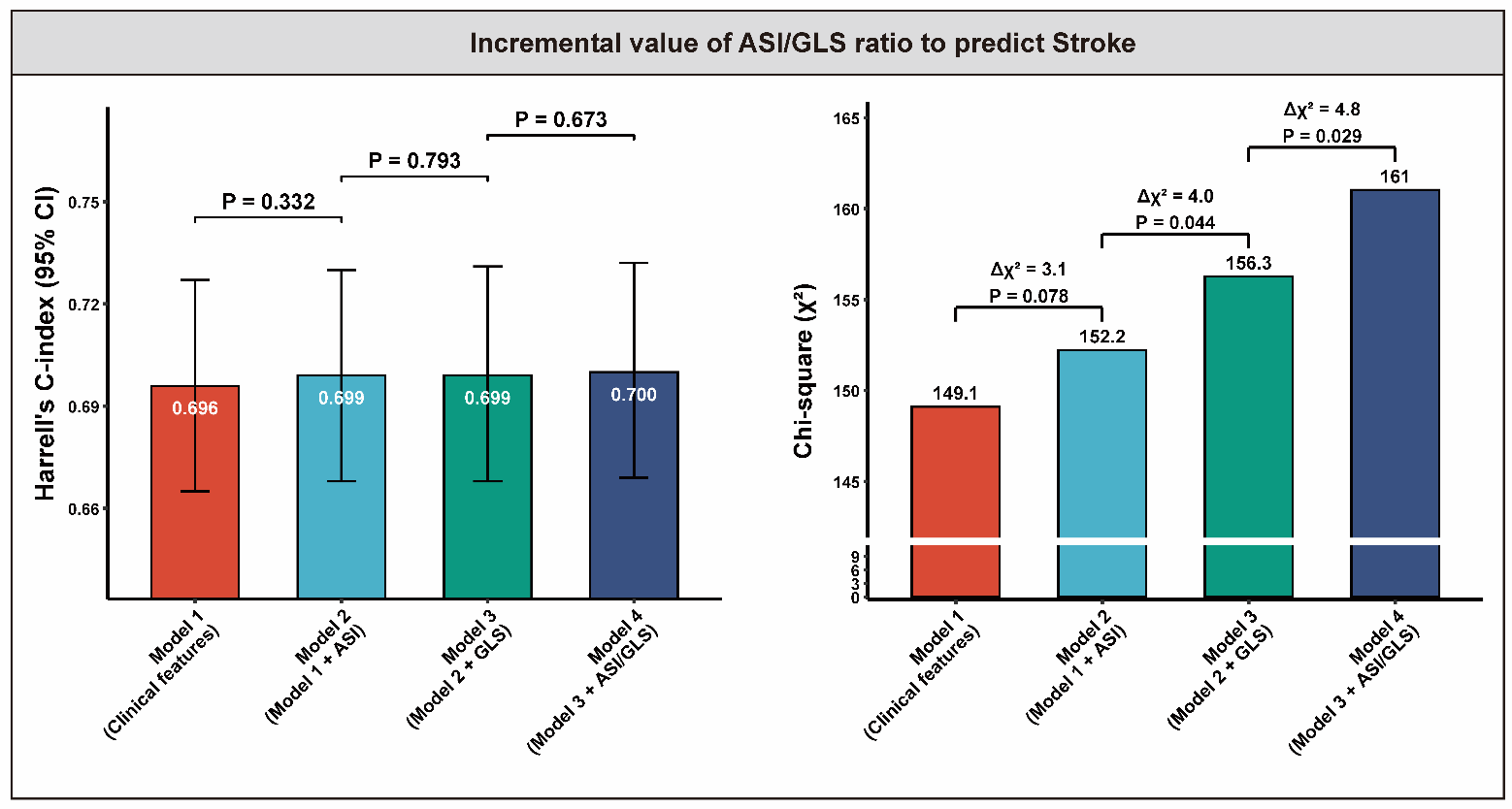


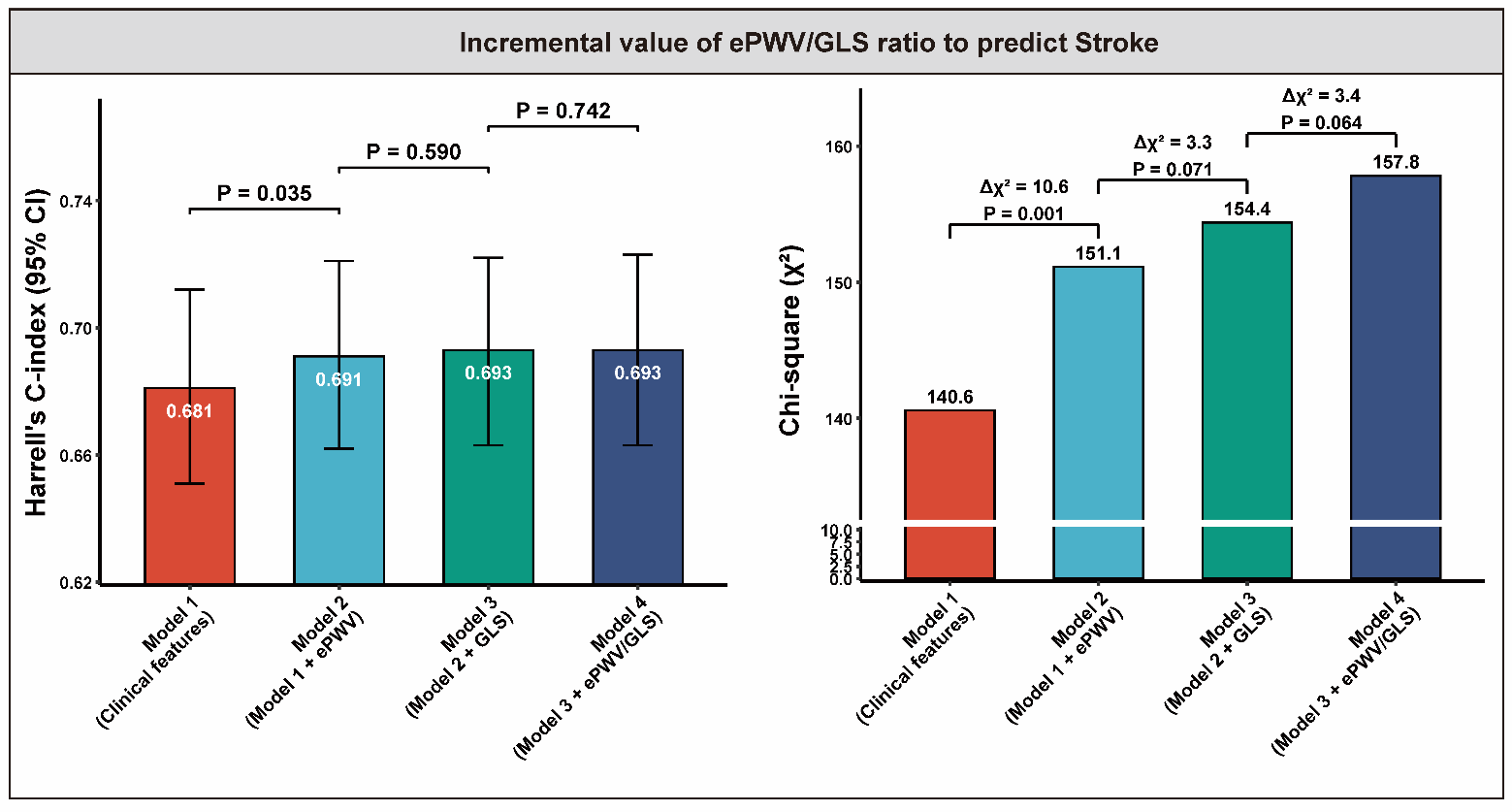


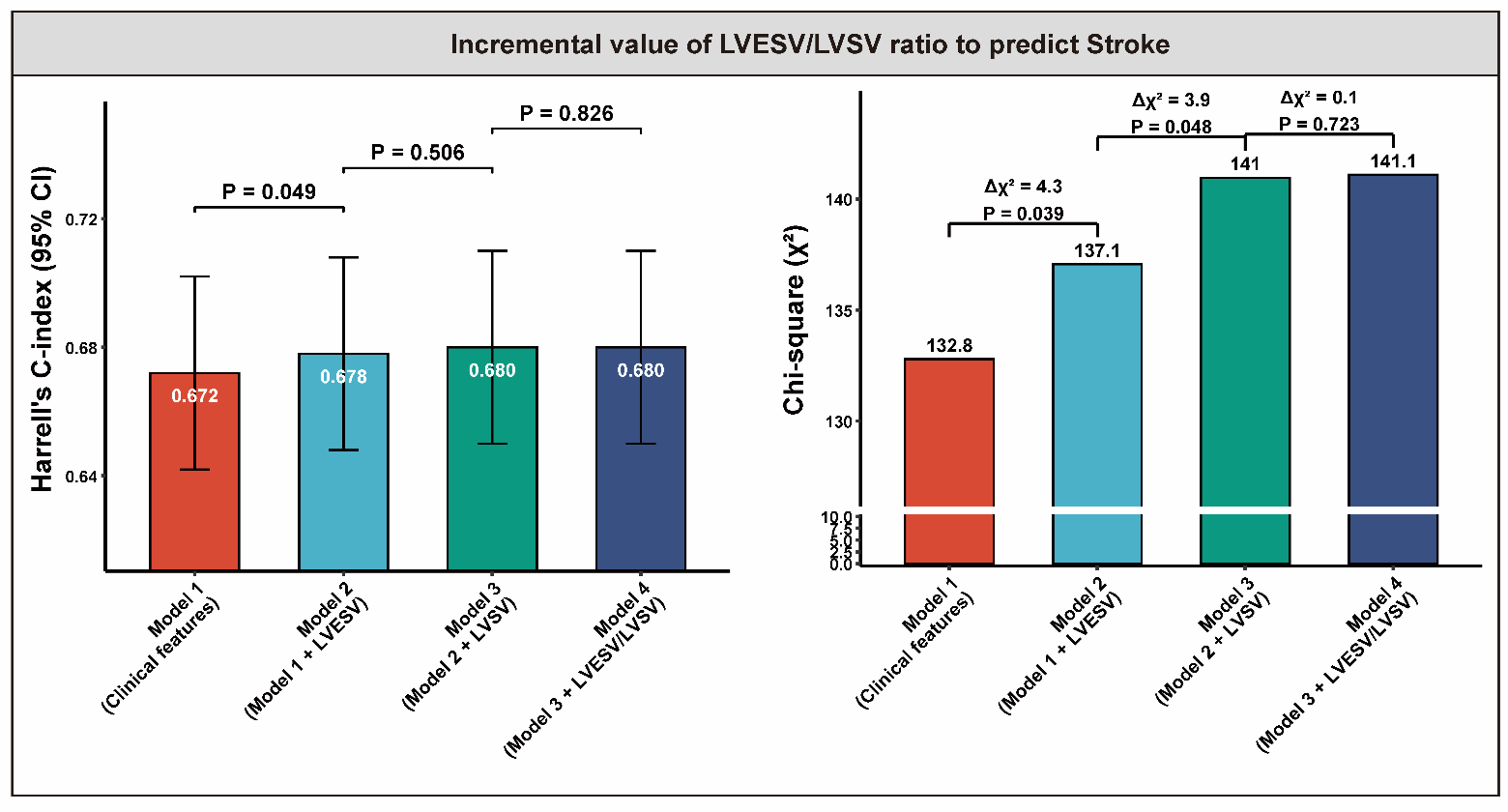


Incremental predictive value of ASI/GLS, ePWV/GLS, and LVESV/LVSV for predicting incident stroke. The left panels display Harrell's C-indices, and the right panels display the likelihood ratio test chi-square (χ²) statistics. Clinical features included age, sex, race, education, body mass index, smoking status, alcohol intake frequency, healthy physical activity, family history of heart disease, prevalent hypertension, dyslipidemia, diabetes, and coronary heart disease.

Model 1 included clinical features only. Model 2 added the numerator component (ASI, ePWV, or LVESV) to Model 1. Model 3 added the denominator component (GLS or LVSV) to Model 2 (representing the model with both constituent components). Model 4 added the VAC parameter (ratio) to Model 3. *P*-values above brackets indicate the statistical significance of the comparison between the indicated models. Δχ² indicates the change in chi-square statistic. Abbreviations: ASI/GLS, ratio of arterial stiffness index to global longitudinal strain; ePWV/GLS, ratio of estimated pulse wave velocity to global longitudinal strain; LVESV/LVSV, ratio of left ventricular end-systolic volume to stroke volume; VAC, ventricular-arterial coupling.

## Figure S7. RCS analysis of the association between VAC parameters and incident HF.


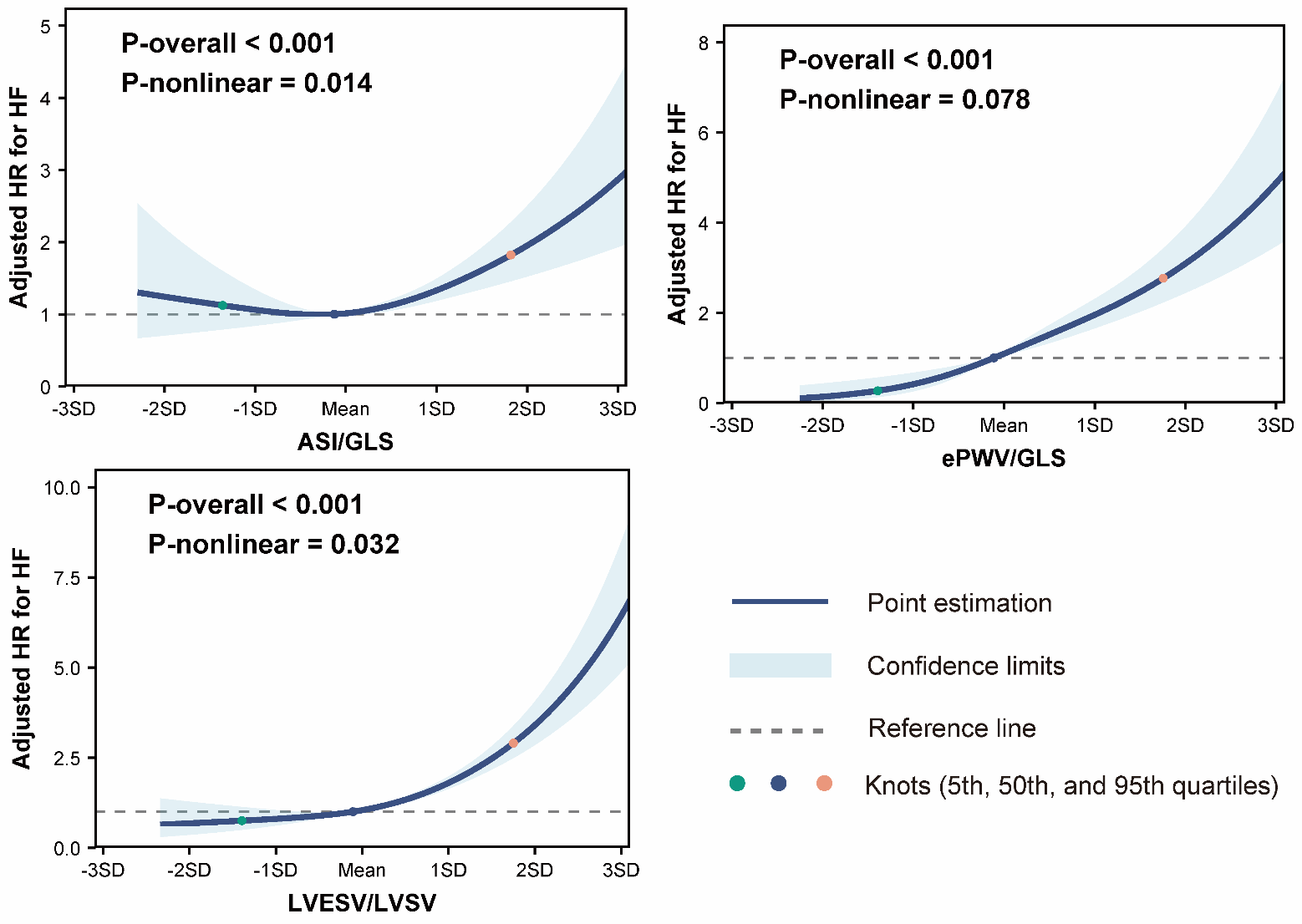


The solid blue lines represent the multivariable-adjusted hazard ratios, and the shaded areas represent the 95% confidence intervals. The dashed line indicates a hazard ratio of 1.00 (reference). Knots were placed at the 5th, 50th, and 95th percentiles to visualize the distribution across the data range.

The models were adjusted for age, sex, race, education, body mass index, smoking status, alcohol intake frequency, healthy physical activity, family history of heart disease, prevalent hypertension, dyslipidemia, diabetes, and coronary heart disease.

Abbreviations: ASI, arterial stiffness index; ePWV, estimated pulse wave velocity; GLS, left ventricular global longitudinal strain; HR, hazard ratio; LVESV, left ventricular end-systolic volume; LVSV, left ventricular stroke volume; RCS, restricted cubic spline; VAC, ventricular-arterial coupling.

## Figure S8. Cumulative incidence of HF according to quartiles of VAC parameters.


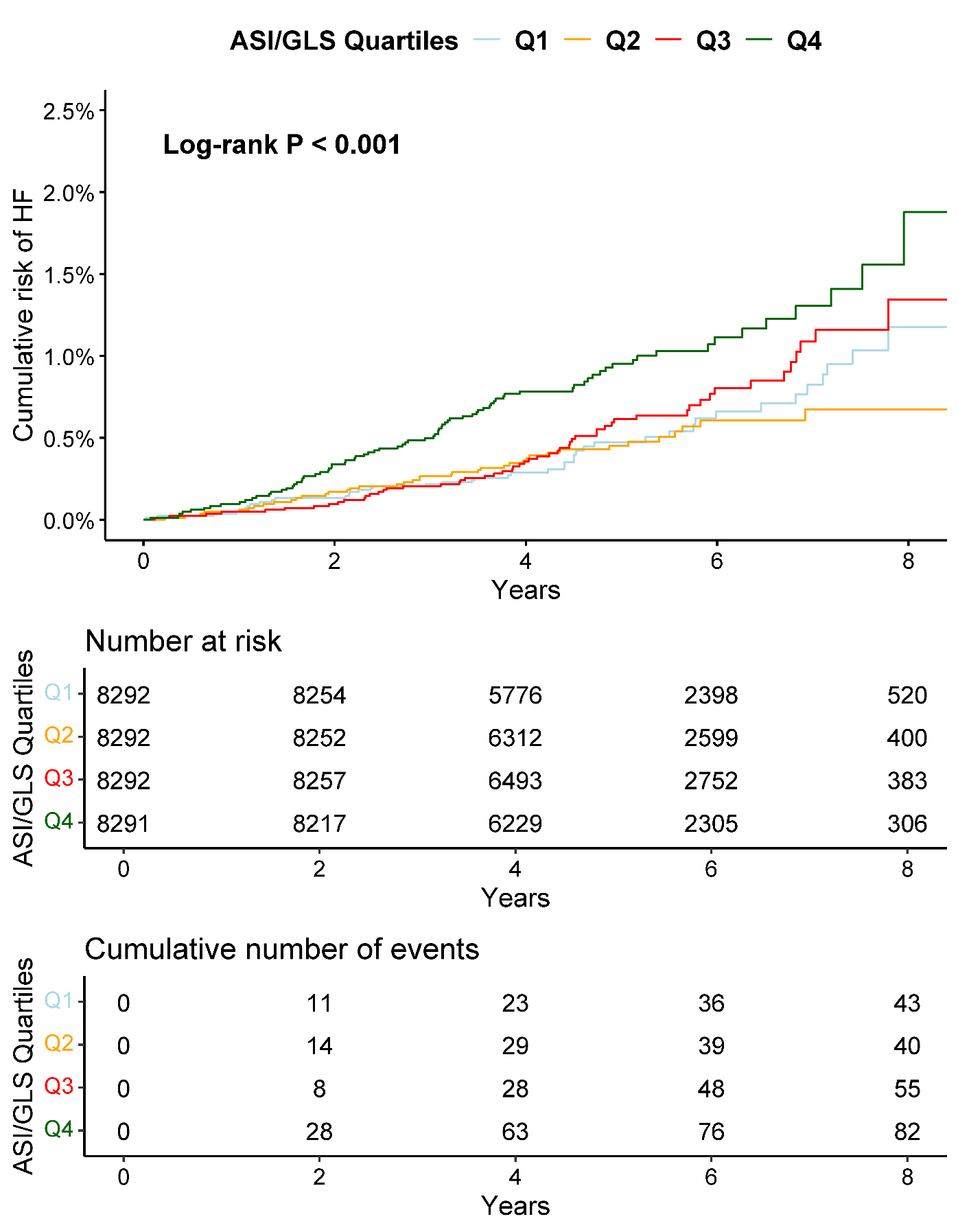


Univariable survival analysis showed the association between increasing quartiles of ASI/GLS and the risk of HF, evaluated using the Kaplan-Meier method and compared using the Log-rank test. Abbreviations: ASI, arterial stiffness index; GLS, left ventricular global longitudinal strain; HF, heart failure; VAC, ventricular-arterial coupling.


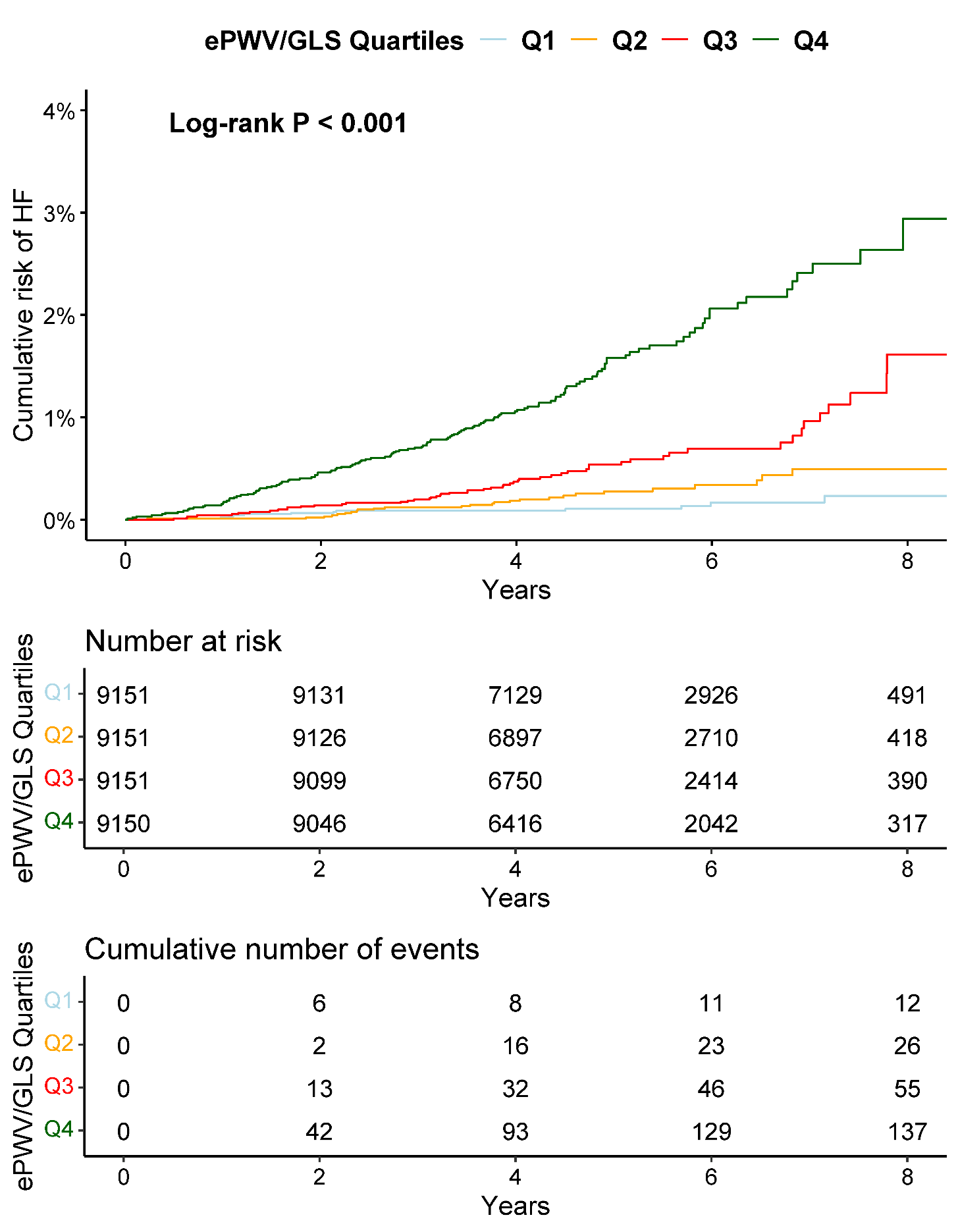


Univariable survival analysis showed the association between increasing quartiles of ePWV/GLS and the risk of HF, evaluated using the Kaplan-Meier method and compared using the Log-rank test. Abbreviations: ePWV, estimated pulse wave velocity; GLS, left ventricular global longitudinal strain; HF, heart failure; VAC, ventricular-arterial coupling.


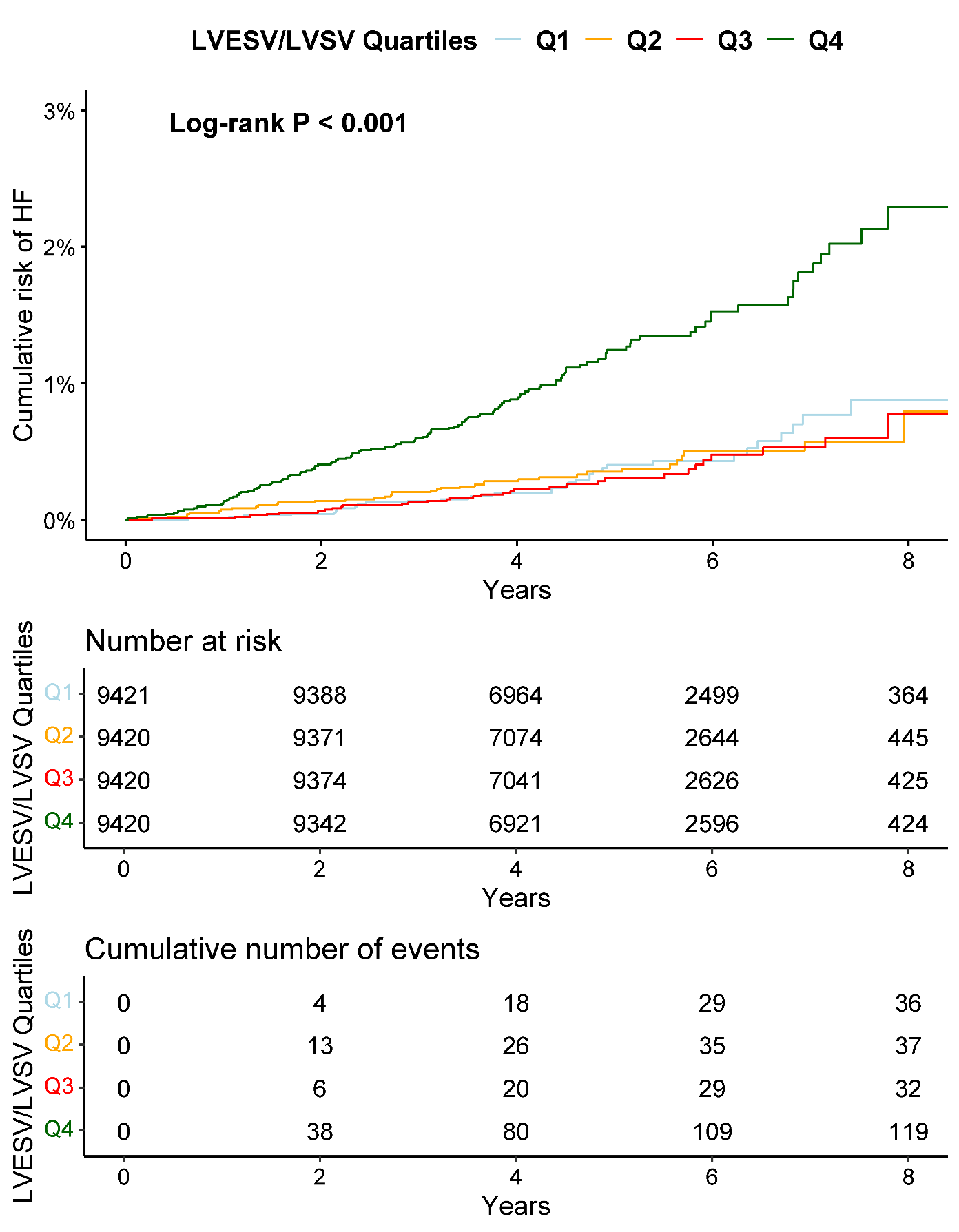


Univariable survival analysis showed the association between increasing quartiles of LVESV/LVSV and the risk of HF, evaluated using the Kaplan-Meier method and compared using the Log-rank test. Abbreviations: HF, heart failure; LVESV, left ventricular end-systolic volume; LVSV, left ventricular stroke volume; VAC, ventricular-arterial coupling.

## Figure S9. Incremental value of VAC parameters over its constituent components for incident HF.


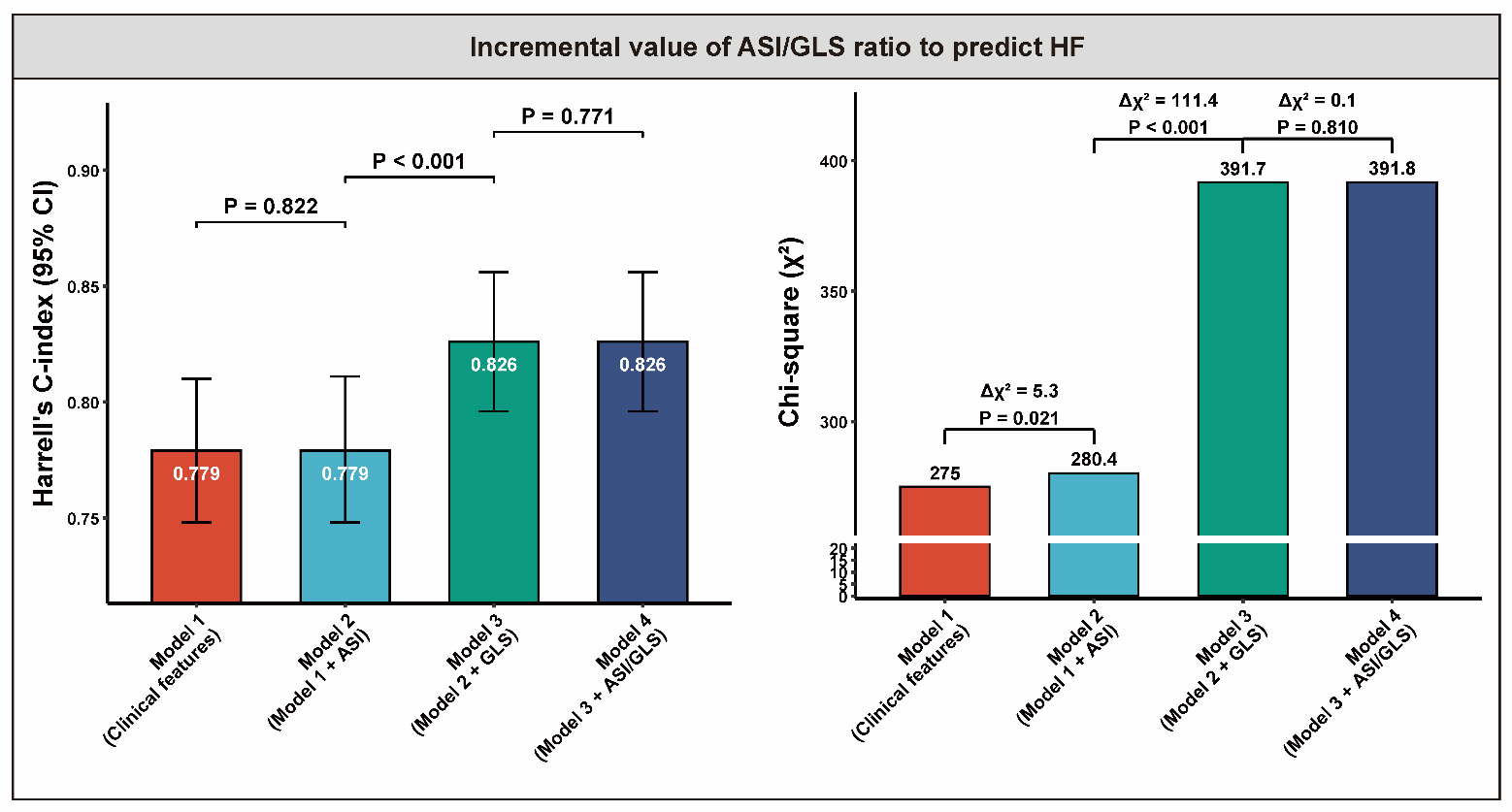


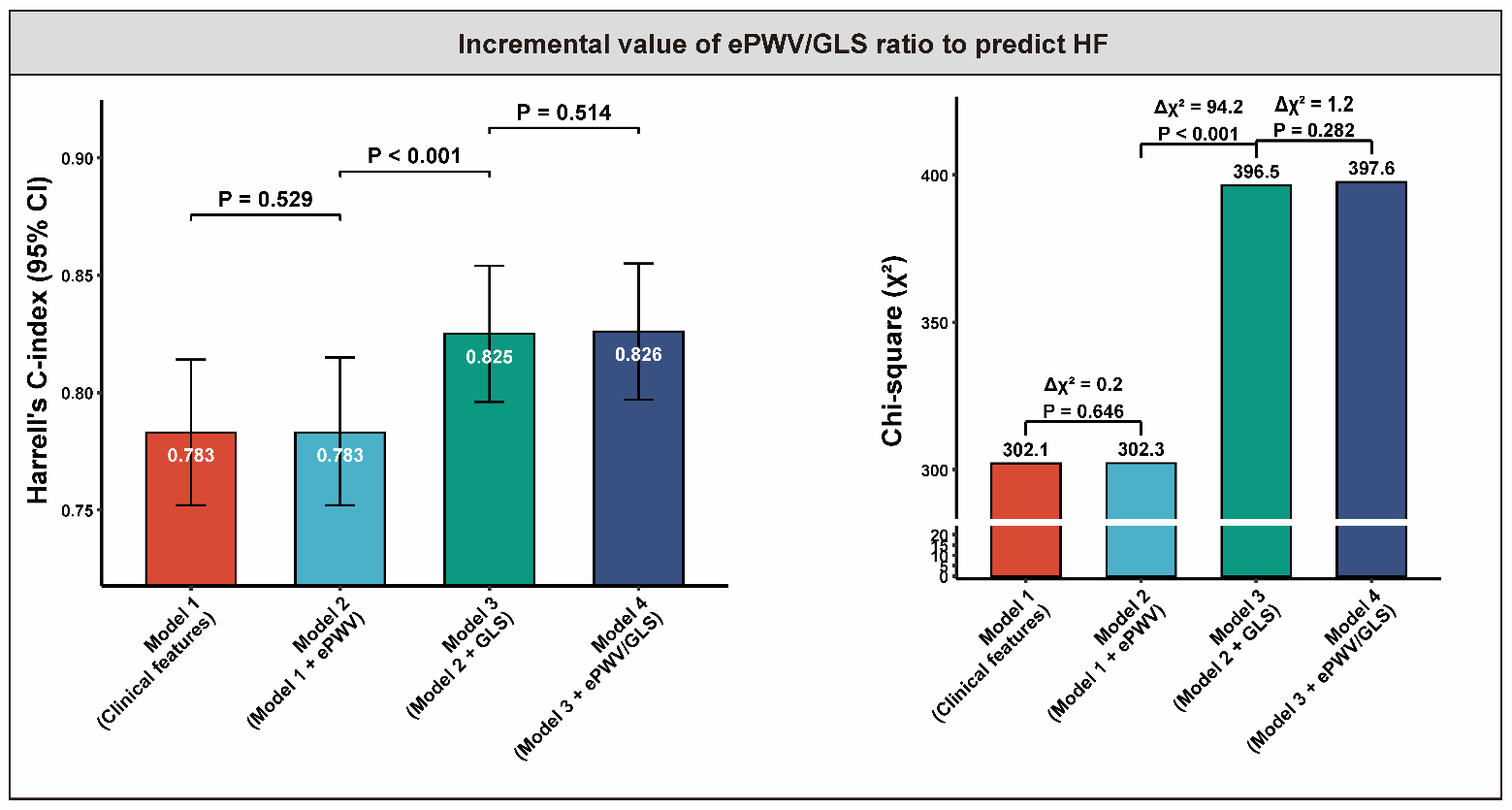


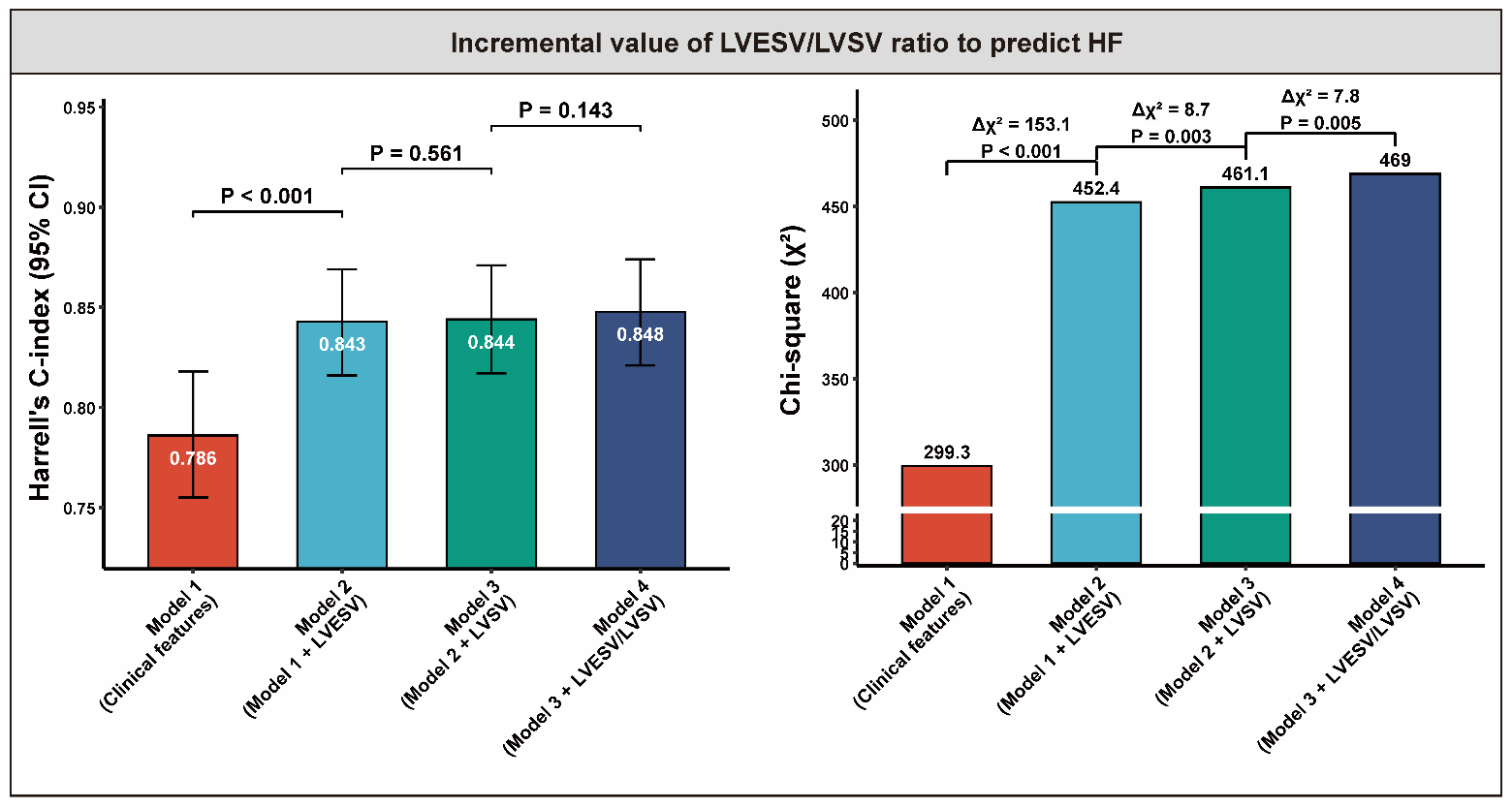


Incremental predictive value of ASI/GLS, ePWV/GLS, and LVESV/LVSV for predicting incident HF. The left panels display Harrell's C-indices, and the right panels display the likelihood ratio test chi-square (χ²) statistics. Clinical features included age, sex, race, education, body mass index, smoking status, alcohol intake frequency, healthy physical activity, family history of heart disease, prevalent hypertension, dyslipidemia, diabetes, and coronary heart disease.

Model 1 included clinical features only. Model 2 added the numerator component (ASI, ePWV, or LVESV) to Model 1. Model 3 added the denominator component (GLS or LVSV) to Model 2 (representing the model with both constituent components). Model 4 added the VAC parameter (ratio) to Model 3. *P*-values above brackets indicate the statistical significance of the comparison between the indicated models. Δχ² indicates the change in chi-square statistic.

Abbreviations: ASI/GLS, ratio of arterial stiffness index to global longitudinal strain; ePWV/GLS, ratio of estimated pulse wave velocity to global longitudinal strain; HF, heart failure; LVESV/LVSV, ratio of left ventricular end-systolic volume to stroke volume; VAC, ventricular-arterial coupling.

## Figure S10. RCS analysis of the association between VAC parameters and incident CHD.


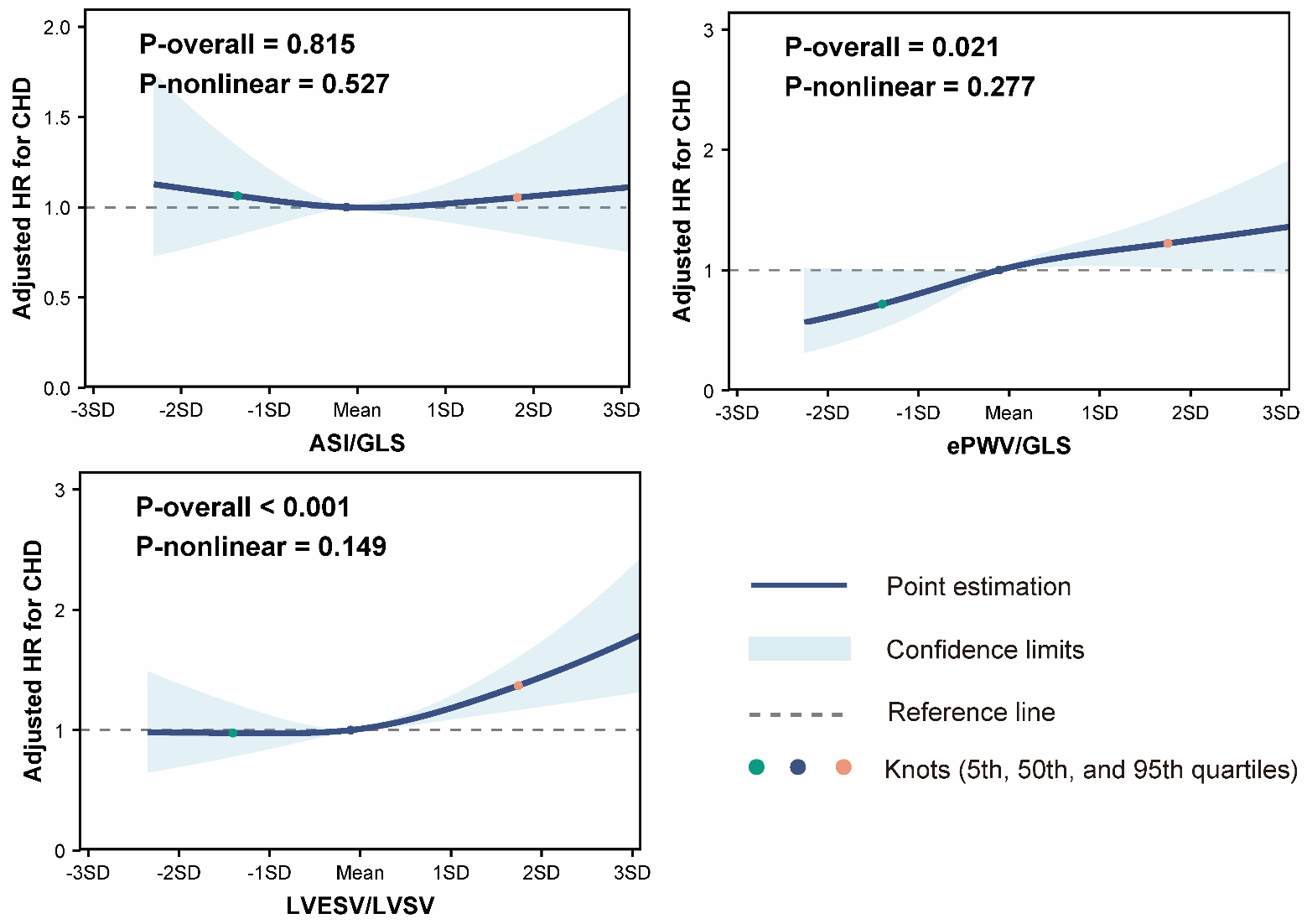


The solid blue lines represent the multivariable-adjusted hazard ratios, and the shaded areas represent the 95% confidence intervals. The dashed line indicates a hazard ratio of 1.00, with the median value used as the reference. Knots were placed at the 5th, 50th, and 95th percentiles to visualize the distribution across the data range.

The models were adjusted for age, sex, race, education, body mass index, smoking status, alcohol intake frequency, healthy physical activity, family history of heart disease, prevalent hypertension, dyslipidemia, and diabetes.

Abbreviations: ASI, arterial stiffness index; ePWV, estimated pulse wave velocity; GLS, left ventricular global longitudinal strain; HR, hazard ratio; LVESV, left ventricular end-systolic volume; LVSV, left ventricular stroke volume; RCS, restricted cubic spline; VAC, ventricular-arterial coupling.

## Figure S11. Cumulative incidence of CHD according to quartiles of VAC parameters.
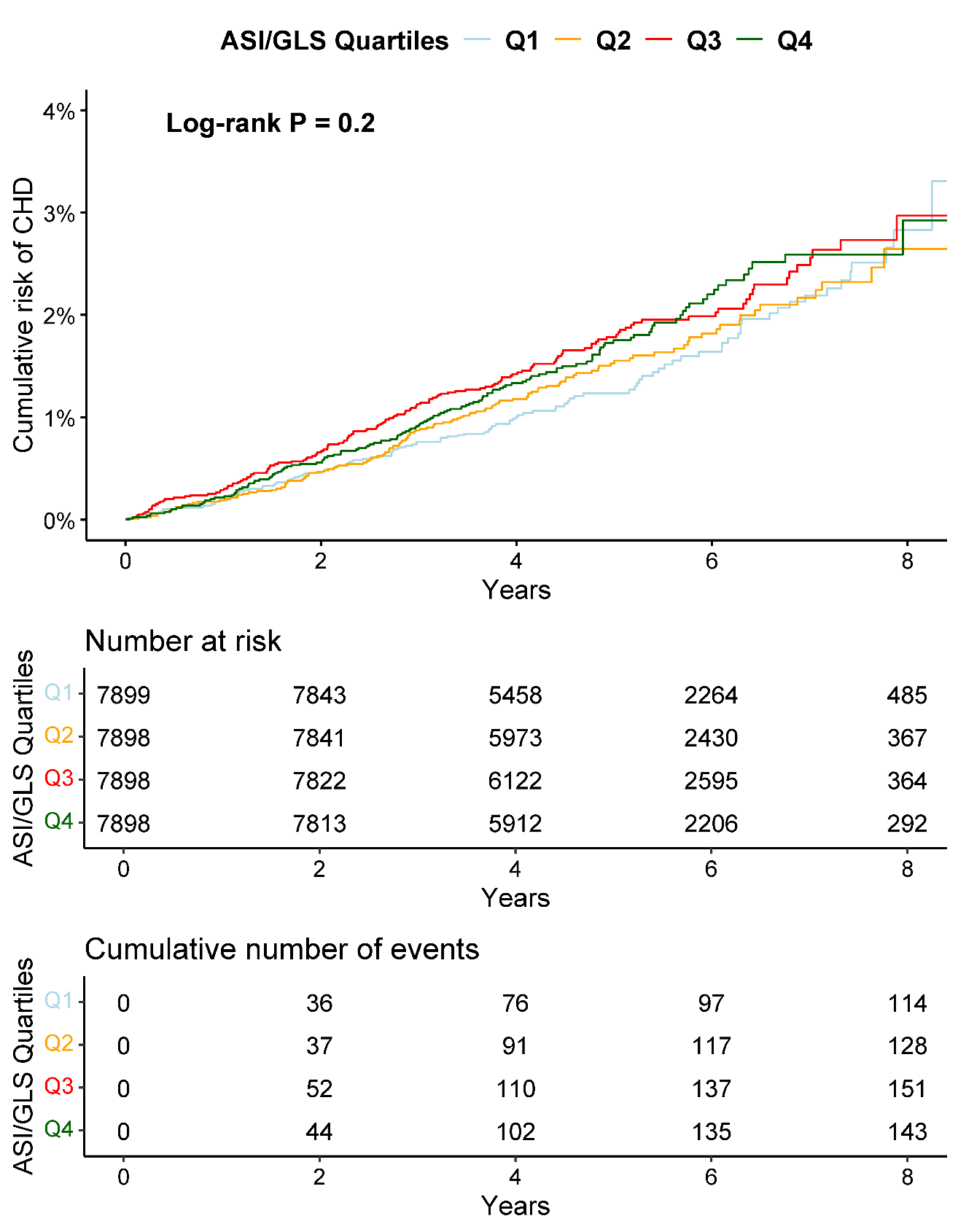


Univariable survival analysis showed the association between increasing quartiles of ASI/GLS and the risk of CHD, evaluated using the Kaplan-Meier method and compared using the Log-rank test. Abbreviations: ASI, arterial stiffness index; CHD, coronary heart disease; GLS, left ventricular global longitudinal strain; VAC, ventricular-arterial coupling.


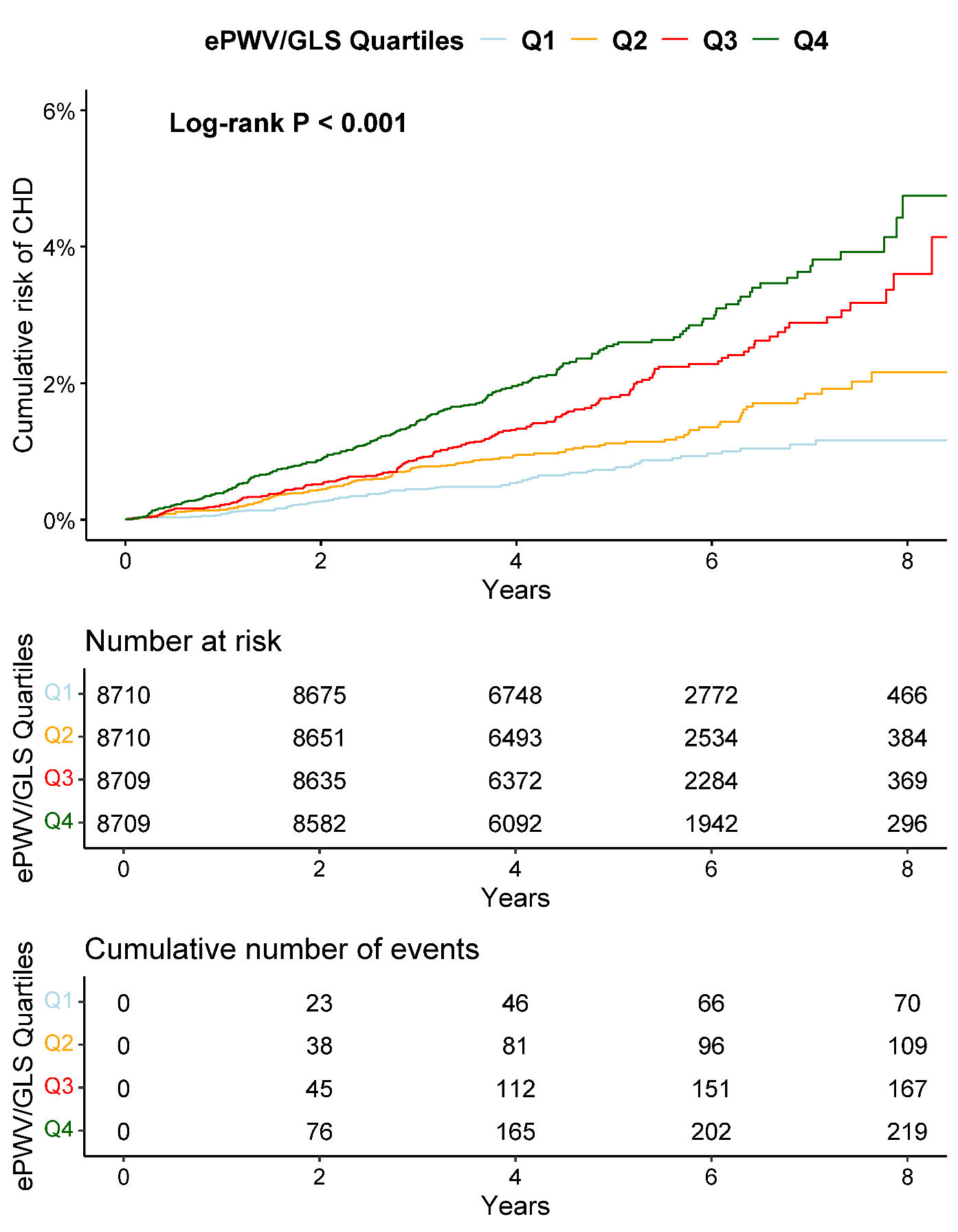


Univariable survival analysis showed the association between increasing quartiles of ePWV/GLS and the risk of CHD, evaluated using the Kaplan-Meier method and compared using the Log-rank test. Abbreviations: CHD, coronary heart disease; ePWV, estimated pulse wave velocity; GLS, left ventricular global longitudinal strain; VAC, ventricular-arterial coupling.


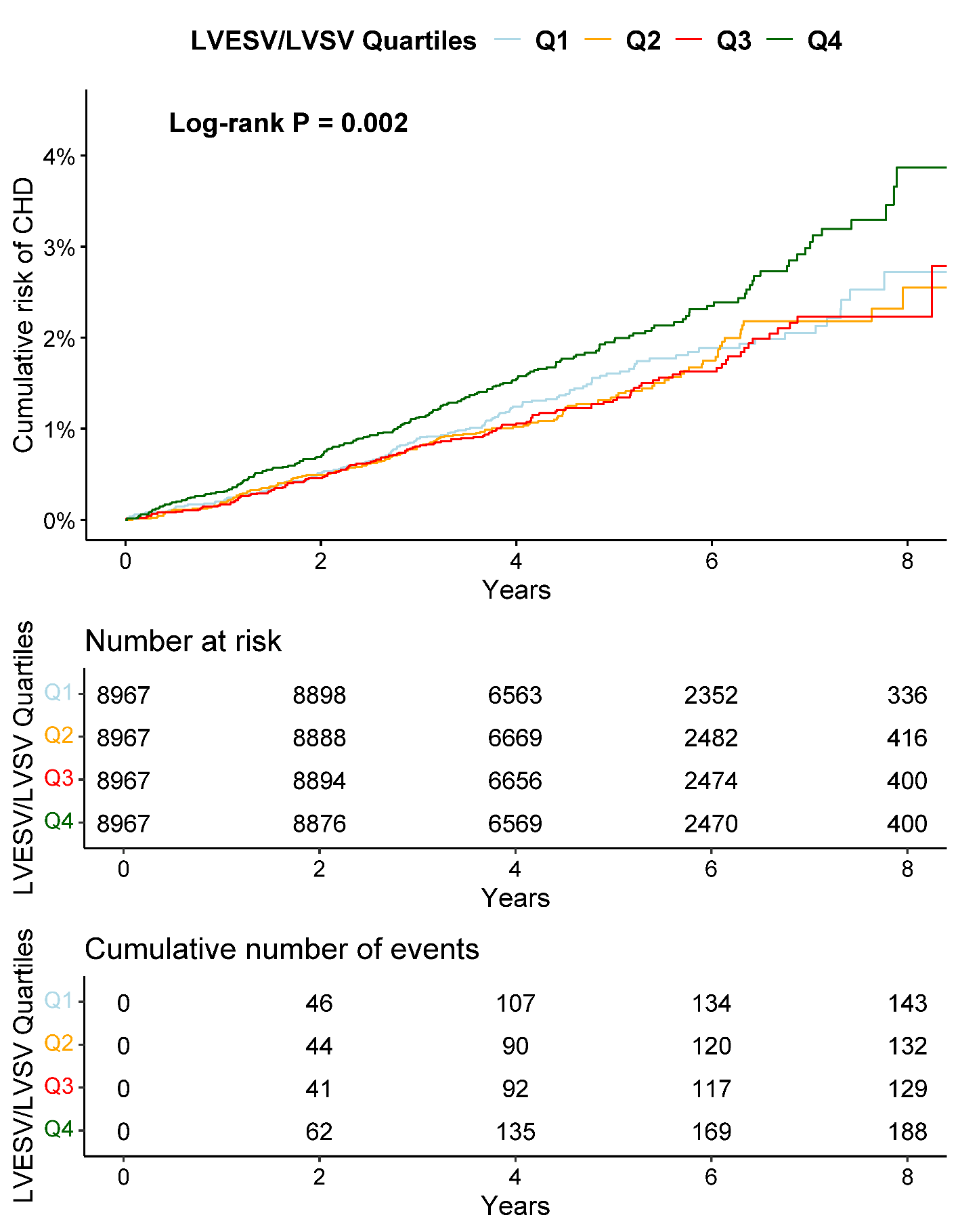


Univariable survival analysis showed the association between increasing quartiles of LVESV/LVSV and the risk of CHD, evaluated using the Kaplan-Meier method and compared using the Log-rank test. Abbreviations: CHD, coronary heart disease; LVESV/LVSV, ratio of left ventricular end-systolic volume to stroke volume; VAC, ventricular-arterial coupling.

## Figure S12. Incremental value of VAC parameters over its constituent components for incident CHD.


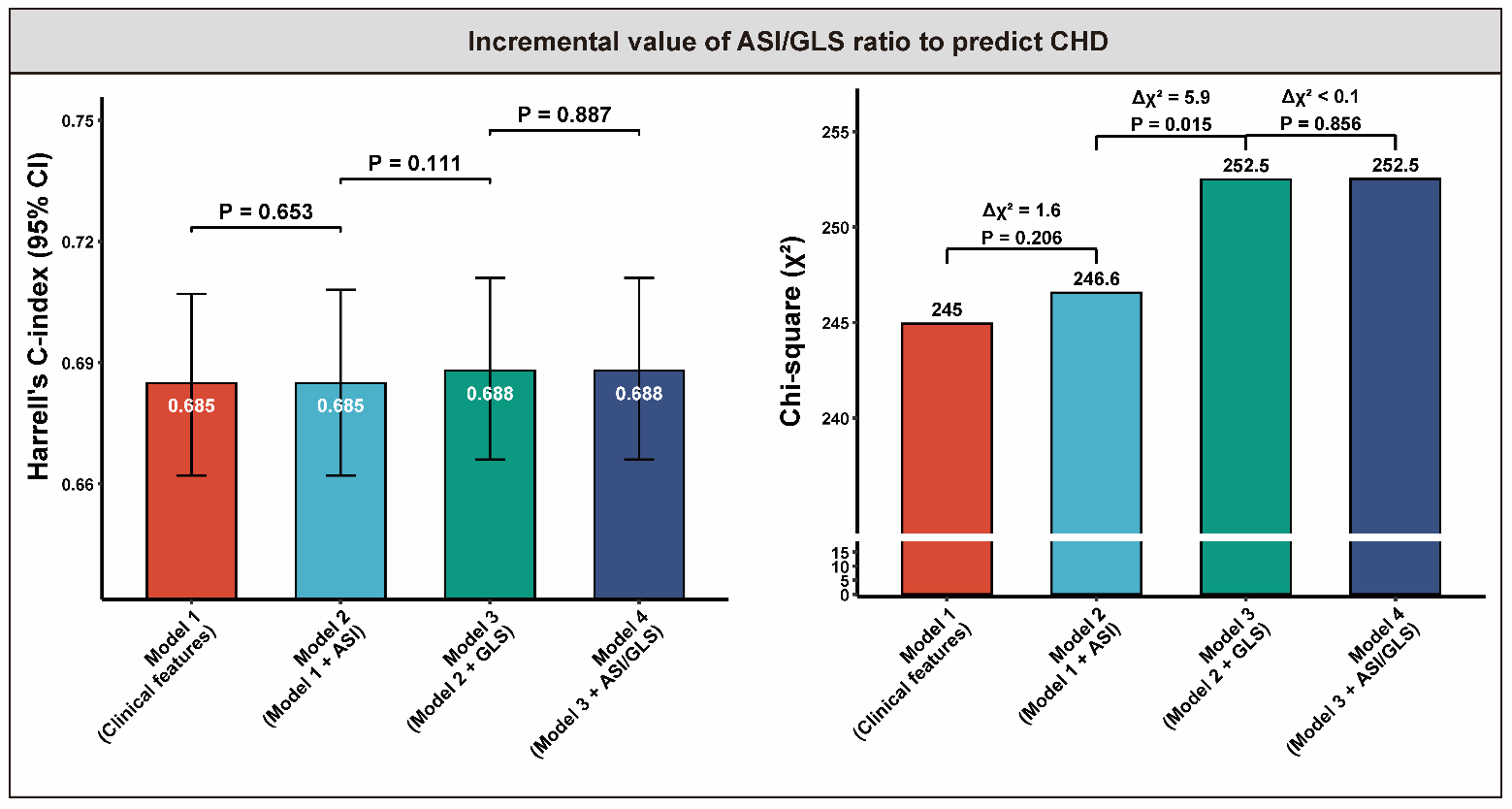


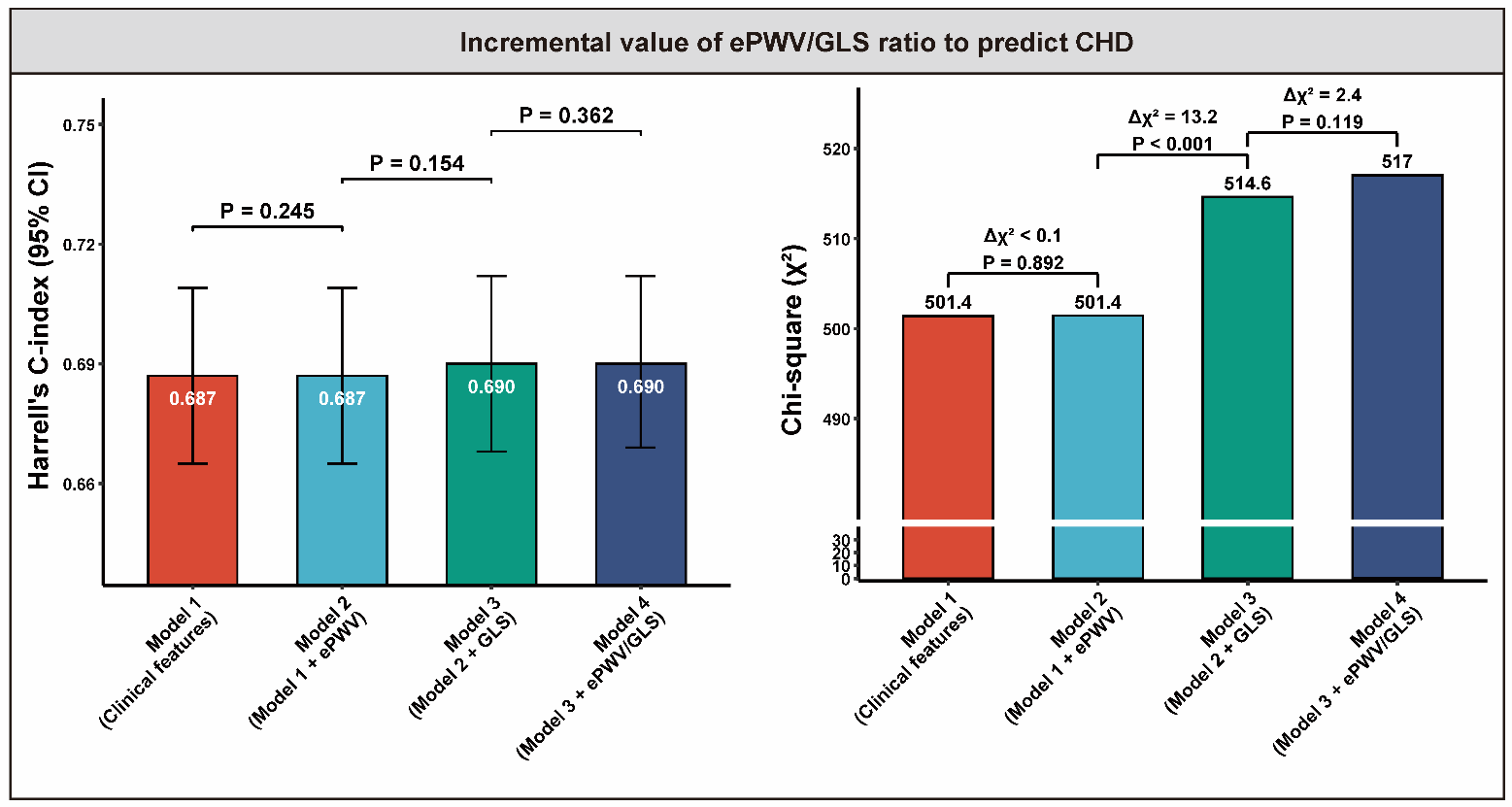


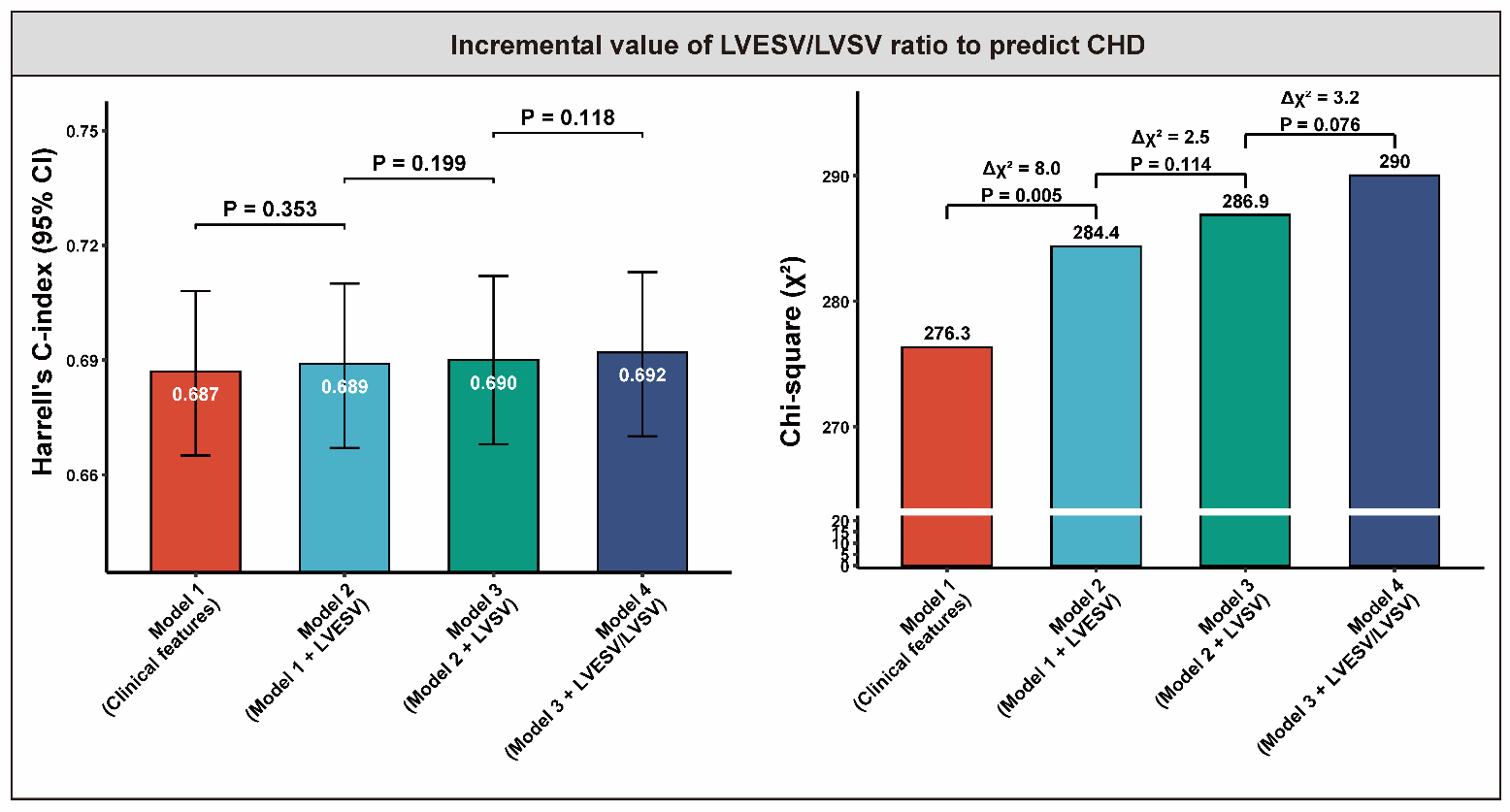


Incremental predictive value of ASI/GLS, ePWV/GLS, and LVESV/LVSV for predicting incident CHD. The left panels display Harrell's C-indices, and the right panels display the likelihood ratio test chi-square (χ²) statistics. Clinical features included age, sex, race, education, body mass index, smoking status, alcohol intake frequency, healthy physical activity, family history of heart disease, prevalent hypertension, dyslipidemia, and diabetes.

Model 1 included clinical features only. Model 2 added the numerator component (ASI, ePWV, or LVESV) to Model 1. Model 3 added the denominator component (GLS or LVSV) to Model 2 (representing the model with both constituent components). Model 4 added the VAC parameter (ratio) to Model 3. *P*-values above brackets indicate the statistical significance of the comparison between the indicated models. Δχ² indicates the change in chi-square statistic.

Abbreviations: ASI/GLS, ratio of arterial stiffness index to global longitudinal strain; CHD, coronary heart disease; ePWV/GLS, ratio of estimated pulse wave velocity to global longitudinal strain; LVESV/LVSV, ratio of left ventricular end-systolic volume to stroke volume; VAC, ventricular-arterial coupling.

## Figure S13. RCS analysis of the association between VAC parameters and all-cause mortality.


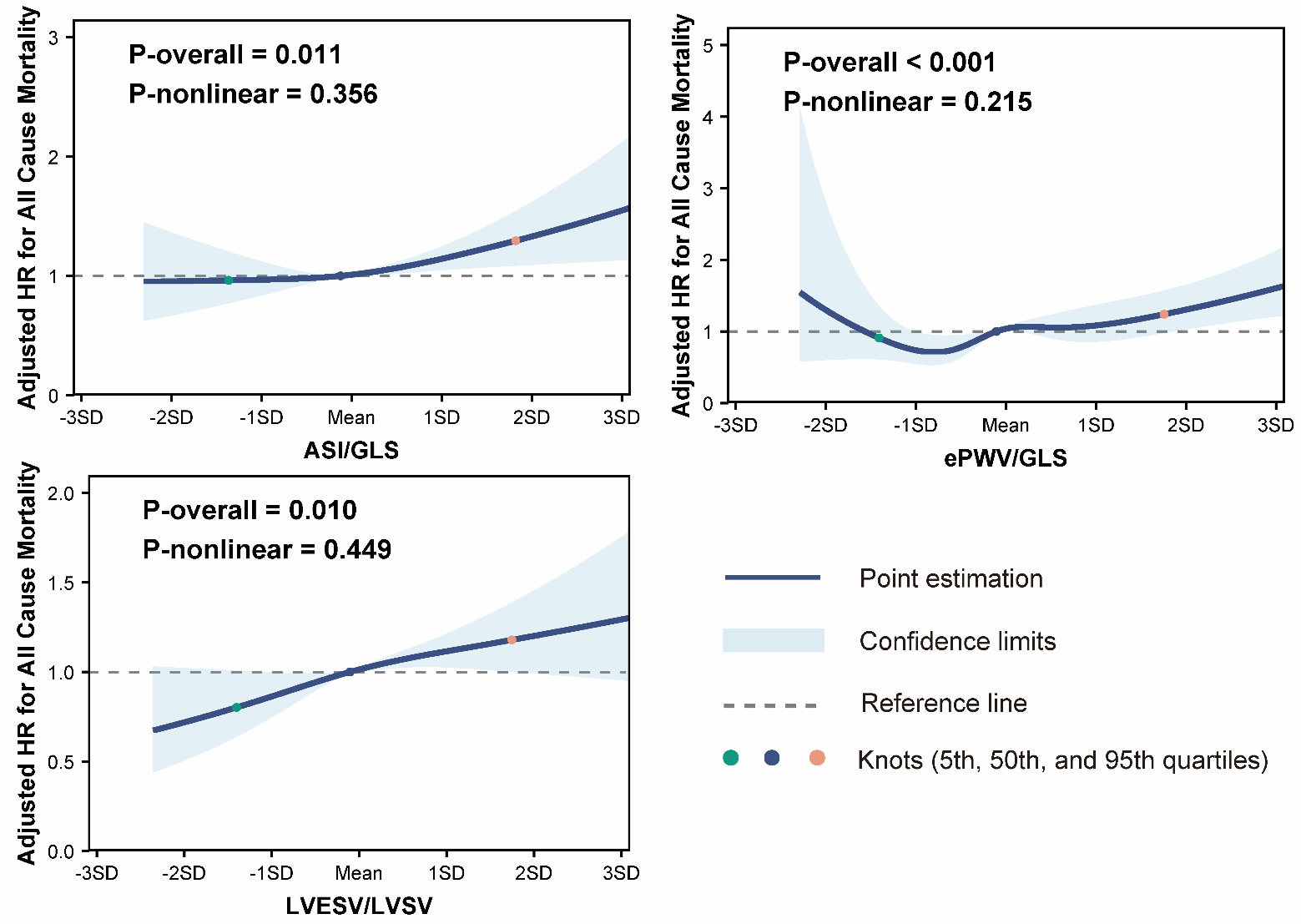


The solid blue lines represent the multivariable-adjusted hazard ratios, and the shaded areas represent the 95% confidence intervals. The dashed line indicates a hazard ratio of 1.00, with the median value used as the reference. Knots were placed at the 5th, 50th, and 95th percentiles to visualize the distribution across the data range.

The models were adjusted for age, sex, race, education, body mass index, smoking status, alcohol intake frequency, healthy physical activity, family history of heart disease, prevalent hypertension, dyslipidemia, diabetes, and coronary heart disease.

Abbreviations: ASI, arterial stiffness index; ePWV, estimated pulse wave velocity; GLS, left ventricular global longitudinal strain; HR, hazard ratio; LVESV, left ventricular end-systolic volume; LVSV, left ventricular stroke volume; RCS, restricted cubic spline; VAC, ventricular-arterial coupling.

## Figure S14. Cumulative incidence of all-cause mortality according to quartiles of VAC parameters.


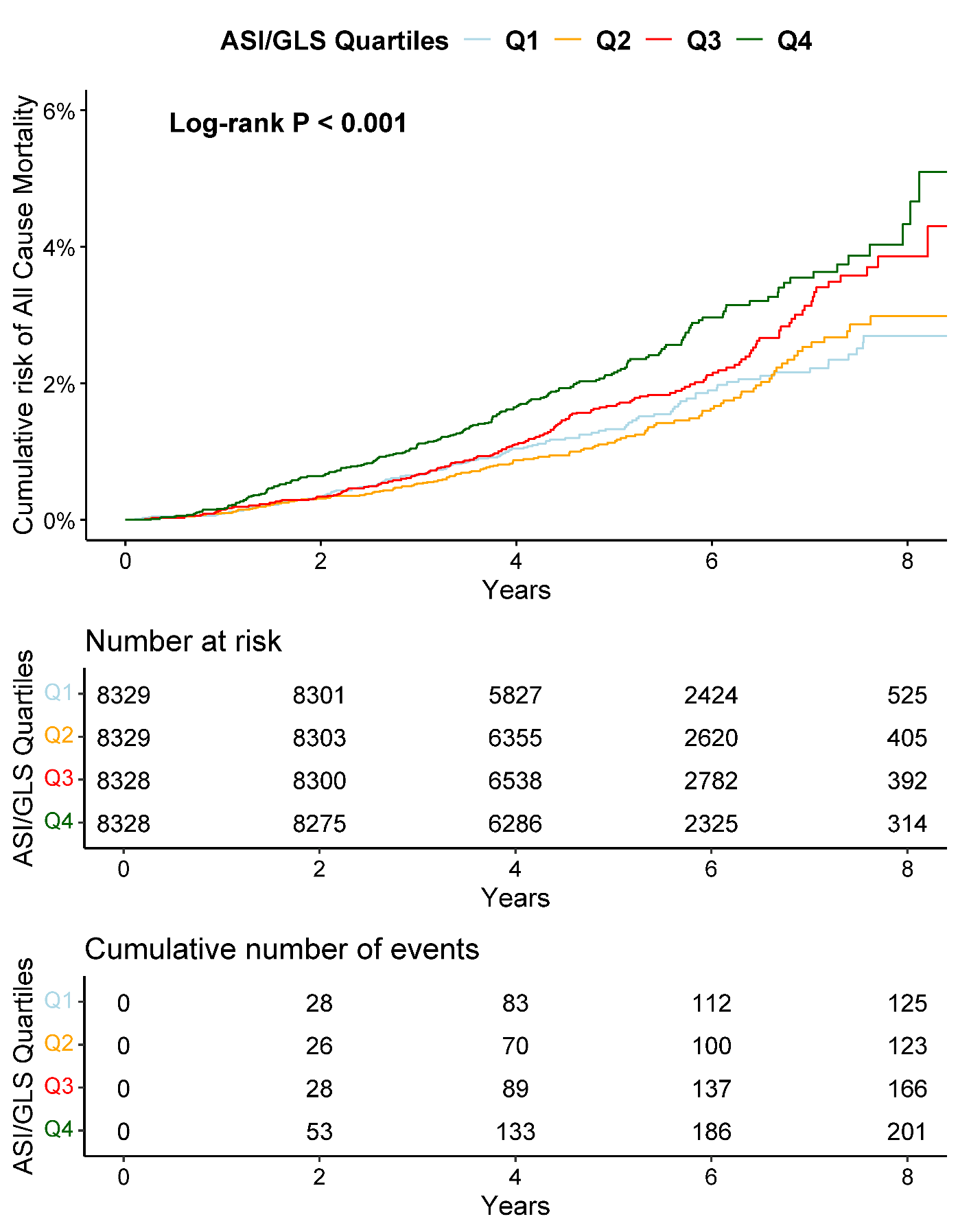


Univariable survival analysis showed the association between increasing quartiles of ASI/GLS and the risk of all-cause mortality, evaluated using the Kaplan-Meier method and compared using the Log-rank test. Abbreviations: ASI/GLS, ratio of arterial stiffness index to global longitudinal strain; VAC, ventricular-arterial coupling.


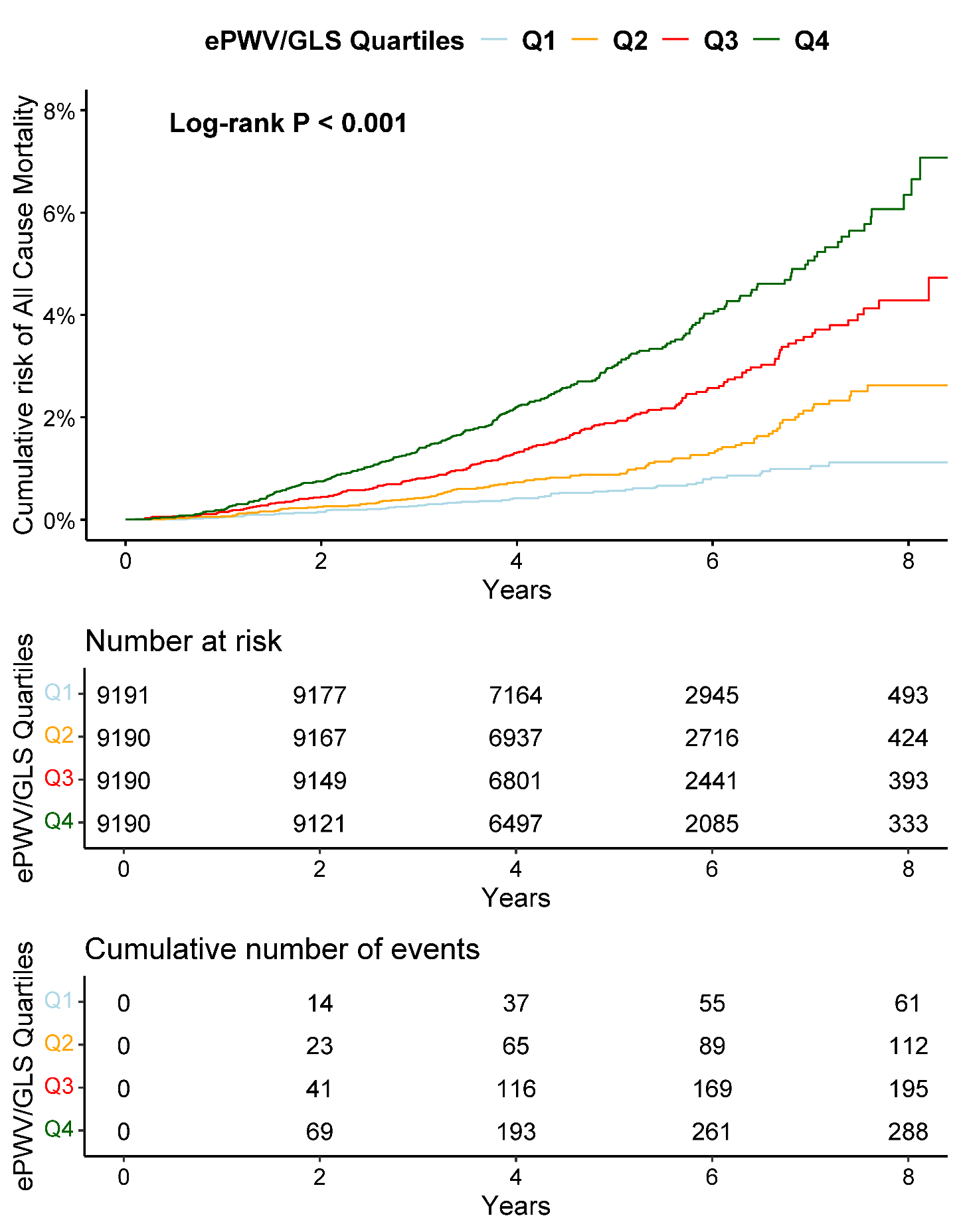


Univariable survival analysis showed the association between increasing quartiles of ePWV/GLS and the risk of all-cause mortality, evaluated using the Kaplan-Meier method and compared using the Log-rank test. Abbreviations: ePWV/GLS, ratio of estimated pulse wave velocity to global longitudinal strain; VAC, ventricular-arterial coupling.


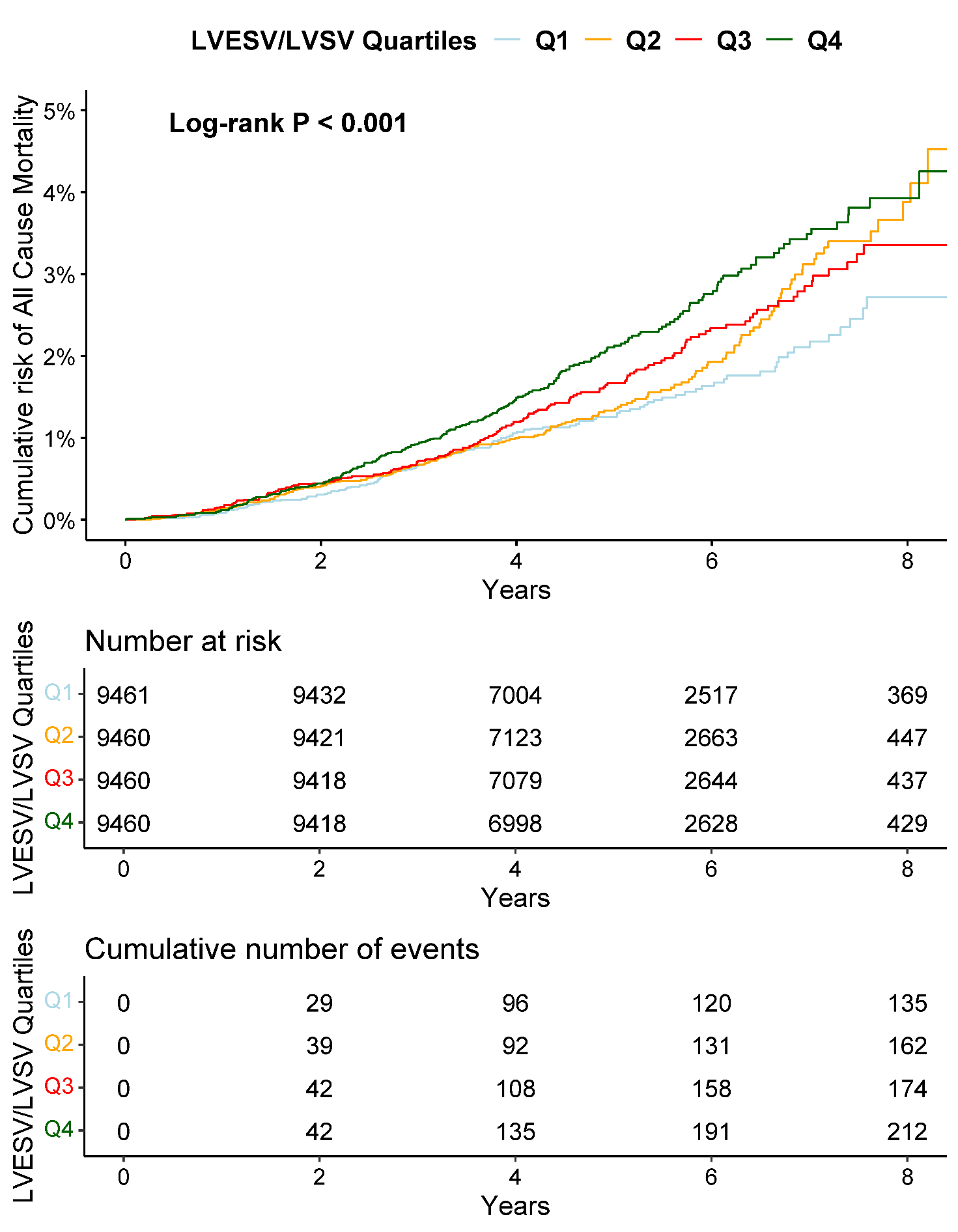


Univariable survival analysis showed the association between increasing quartiles of LVESV/LVSV and the risk of all-cause mortality, evaluated using the Kaplan-Meier method and compared using the Log-rank test. Abbreviations: LVESV/LVSV, ratio of left ventricular end-systolic volume to stroke volume; VAC, ventricular-arterial coupling.

## Figure S15. Incremental value of VAC parameters over its constituent components for all-cause mortality.


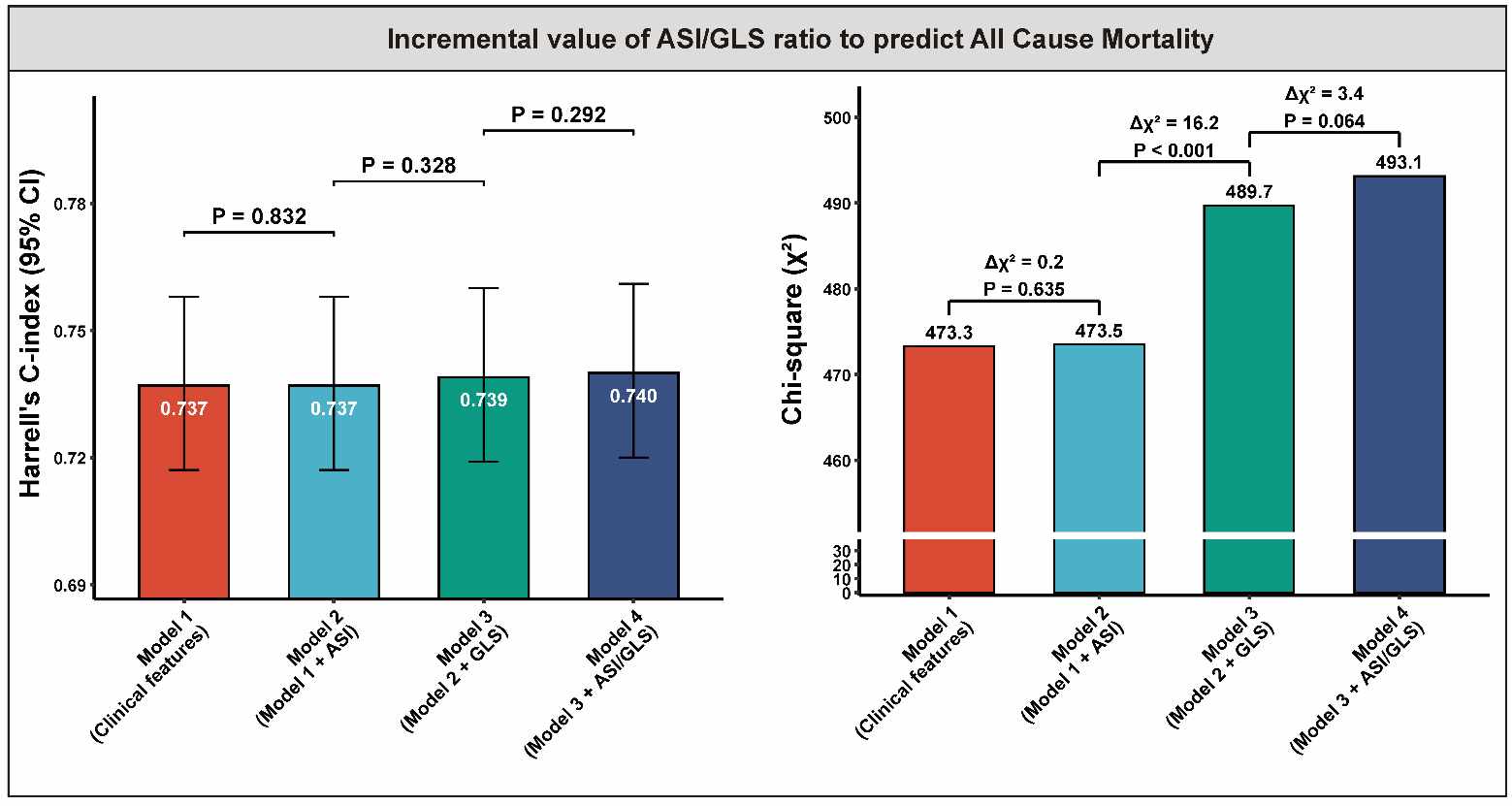


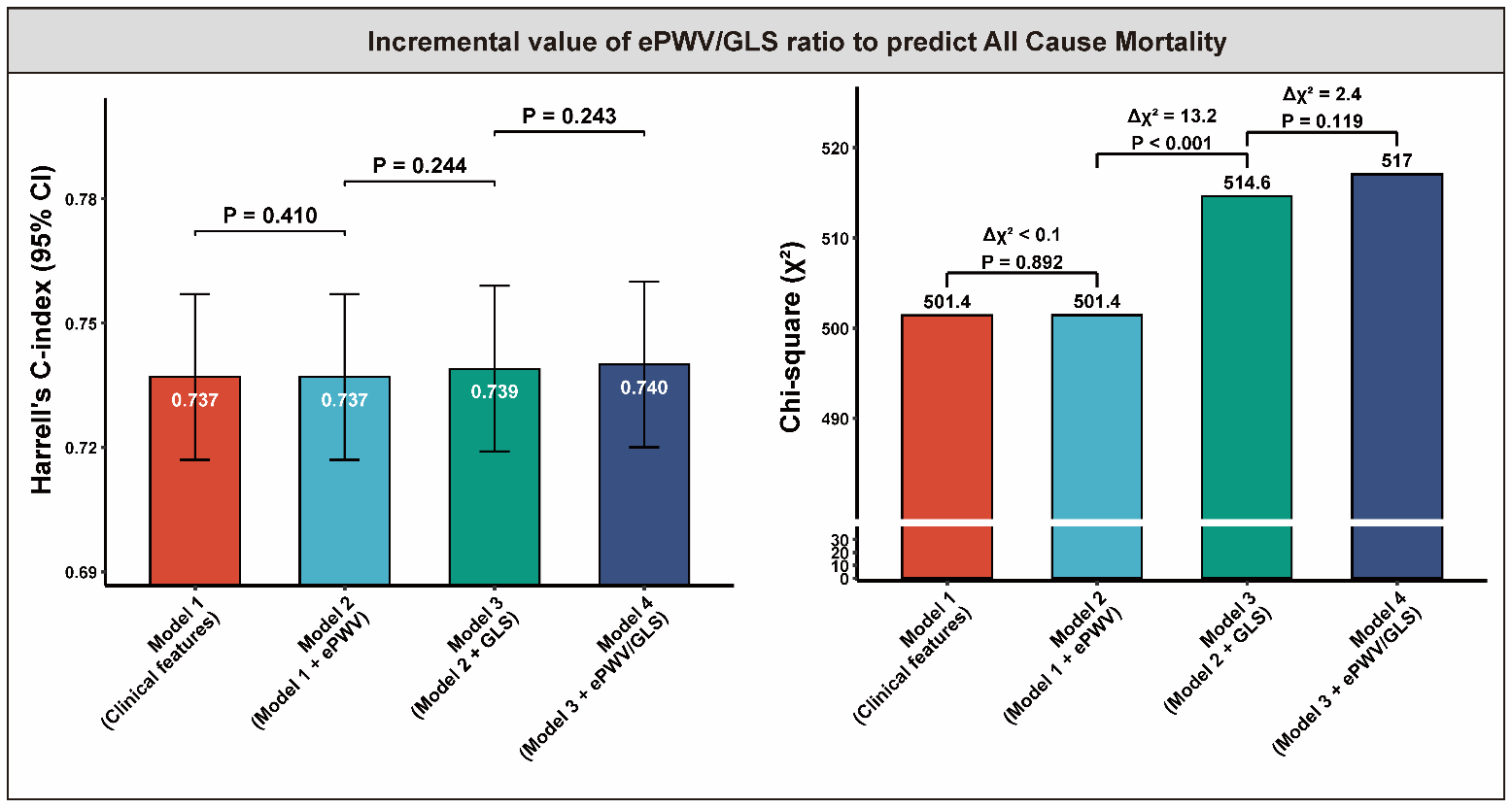


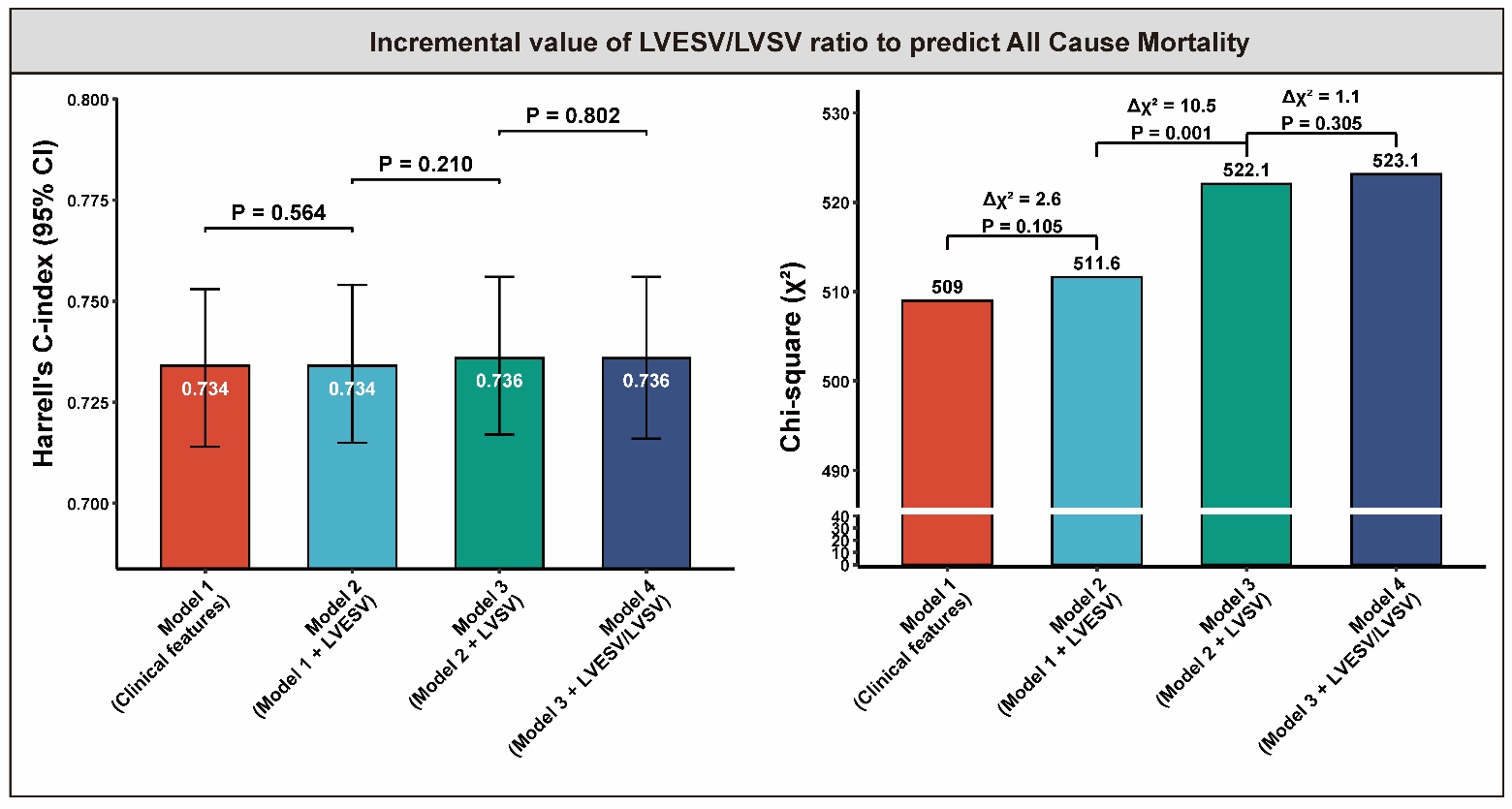


Incremental predictive value of ASI/GLS, ePWV/GLS, and LVESV/LVSV for predicting all-cause mortality. The left panels display Harrell's C-indices, and the right panels display the likelihood ratio test chi-square (χ²) statistics. Clinical features included age, sex, race, education, body mass index, smoking status, alcohol intake frequency, healthy physical activity, family history of heart disease, prevalent hypertension, dyslipidemia, diabetes, and coronary heart disease.

Model 1 included clinical features only. Model 2 added the numerator component (ASI, ePWV, or LVESV) to Model 1. Model 3 added the denominator component (GLS or LVSV) to Model 2 (representing the model with both constituent components). Model 4 added the VAC parameter (ratio) to Model 3. *P*-values above brackets indicate the statistical significance of the comparison between the indicated models. Δχ² indicates the change in chi-square statistic.

Abbreviations: ASI/GLS, ratio of arterial stiffness index to global longitudinal strain; ePWV/GLS, ratio of estimated pulse wave velocity to global longitudinal strain; LVESV/LVSV, ratio of left ventricular end-systolic volume to stroke volume; VAC, ventricular-arterial coupling.

## Figure S16. RCS analysis of the association between VAC parameters and CVD mortality.


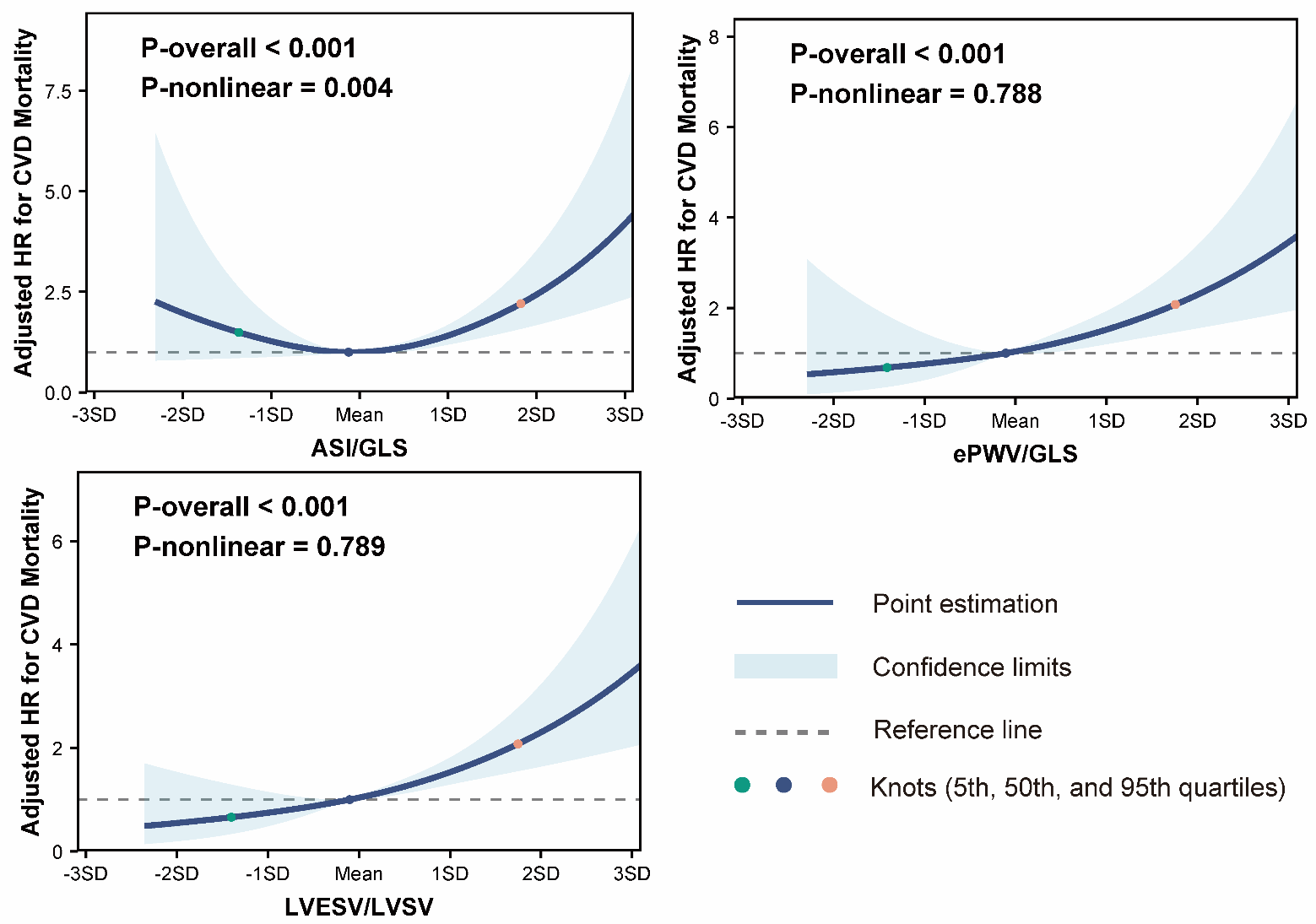


The solid blue lines represent the multivariable-adjusted hazard ratios, and the shaded areas represent the 95% confidence intervals. The dashed line indicates a hazard ratio of 1.00, with the median value used as the reference. Knots were placed at the 5th, 50th, and 95th percentiles to visualize the distribution across the data range.

The models were adjusted for age, sex, race, education, body mass index, smoking status, alcohol intake frequency, healthy physical activity, family history of heart disease, prevalent hypertension, dyslipidemia, diabetes, and coronary heart disease.

Abbreviations: ASI, arterial stiffness index; CVD, cardiovascular disease; ePWV, estimated pulse wave velocity; GLS, left ventricular global longitudinal strain; HR, hazard ratio; LVESV, left ventricular end-systolic volume; LVSV, left ventricular stroke volume; RCS, restricted cubic spline; VAC, ventricular-arterial coupling.

## Figure S17. Cumulative incidence of CVD mortality according to quartiles of VAC parameters.


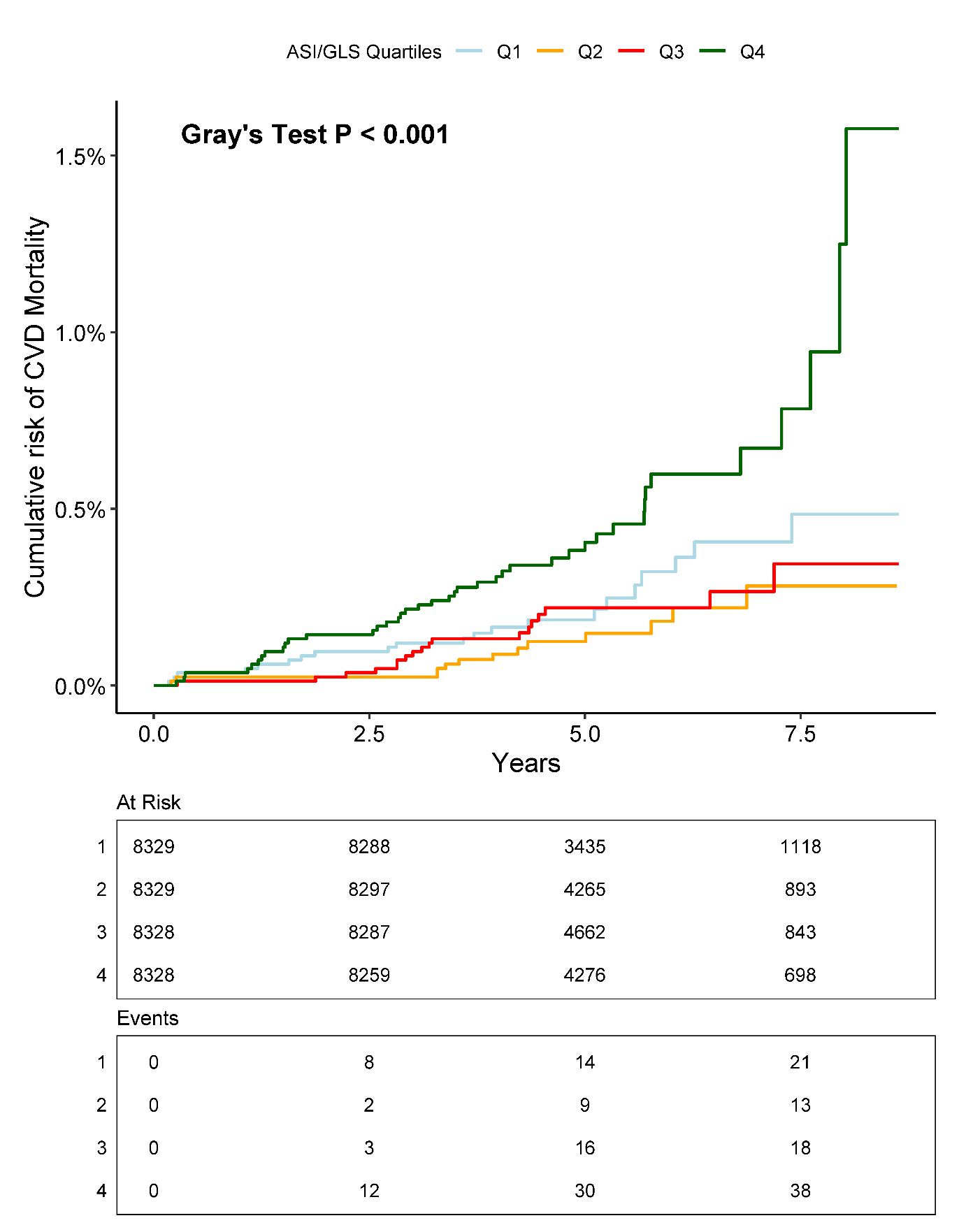


Univariable analysis showed the association between increasing quartiles of ASI/GLS and the risk of CVD mortality, estimated using the cumulative incidence function method accounting for competing risks and compared using Gray's test. Abbreviations: ASI/GLS, ratio of arterial stiffness index to global longitudinal strain; CVD, cardiovascular disease; VAC, ventricular-arterial coupling.


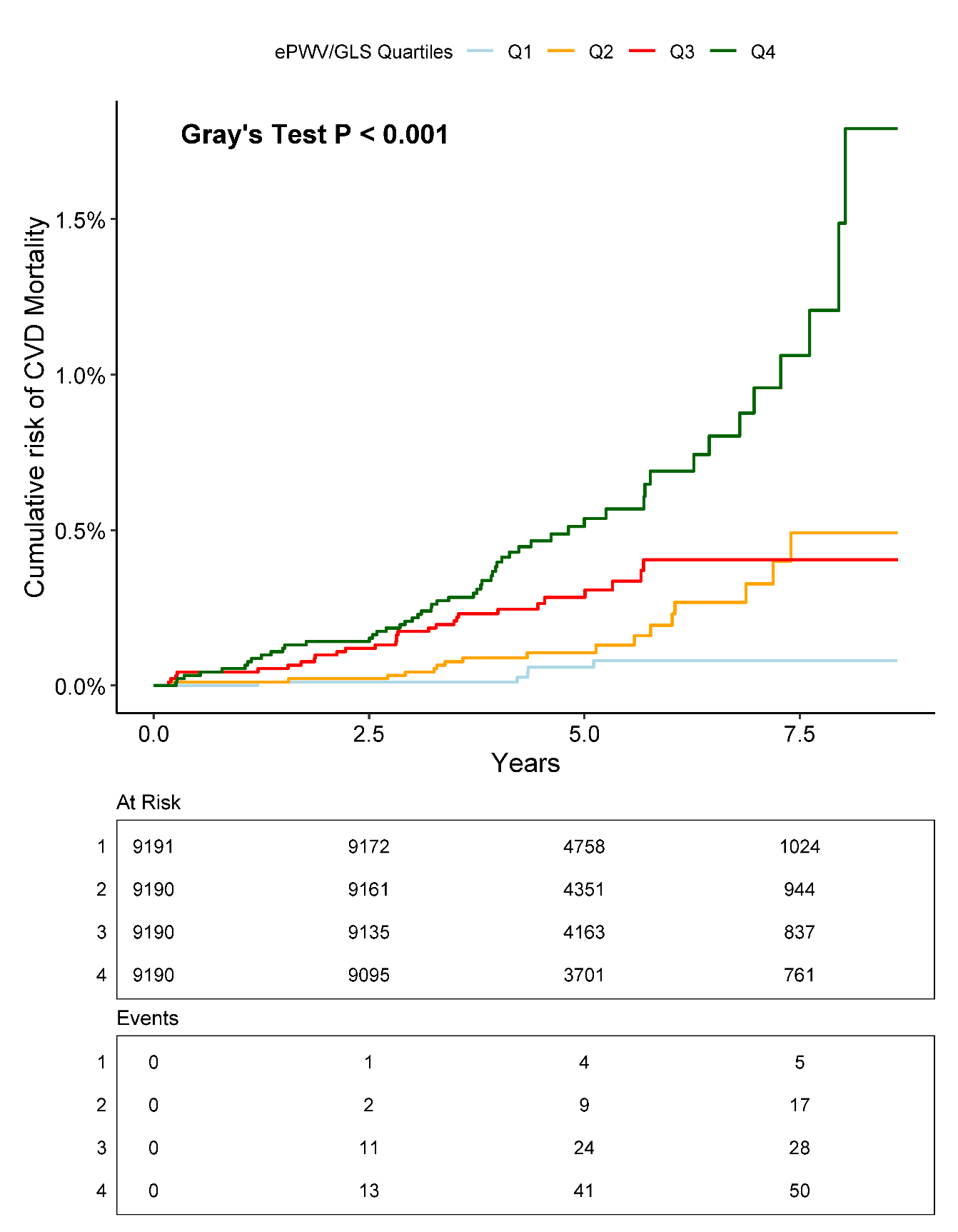


Univariable analysis showed the association between increasing quartiles of ePWV/GLS and the risk of CVD mortality, estimated using the cumulative incidence function method accounting for competing risks and compared using Gray's test. Abbreviations: CVD, cardiovascular disease; ePWV/GLS, ratio of estimated pulse wave velocity to global longitudinal strain; VAC, ventricular-arterial coupling.


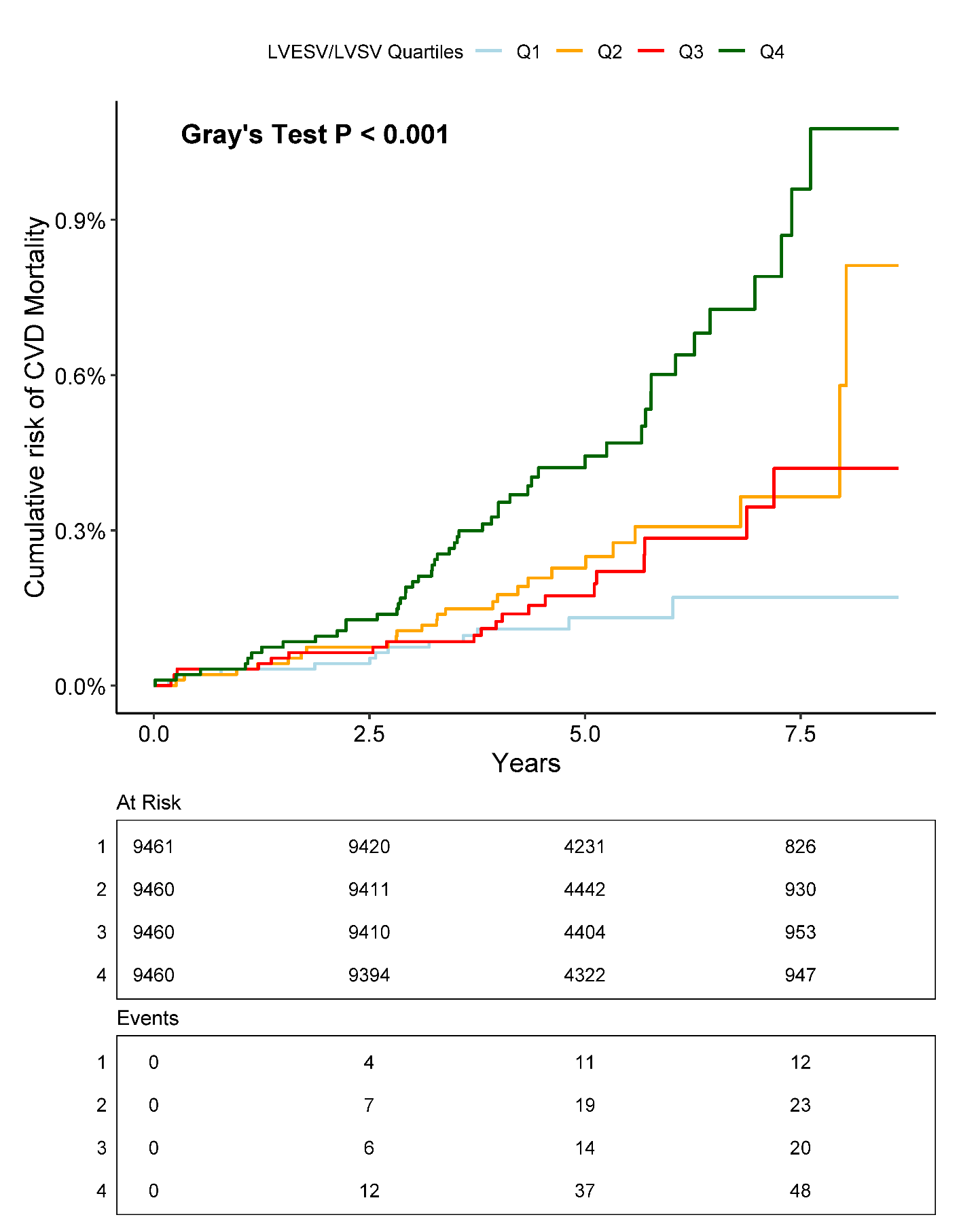


Univariable analysis showed the association between increasing quartiles of LVESV/LVSV and the risk of CVD mortality, estimated using the cumulative incidence function method accounting for competing risks and compared using Gray's test. Abbreviations: CVD, cardiovascular disease; LVESV/LVSV, ratio of left ventricular end-systolic volume to stroke volume; VAC, ventricular-arterial coupling.

## Figure S18. Incremental value of VAC parameters over its constituent components for CVD mortality.


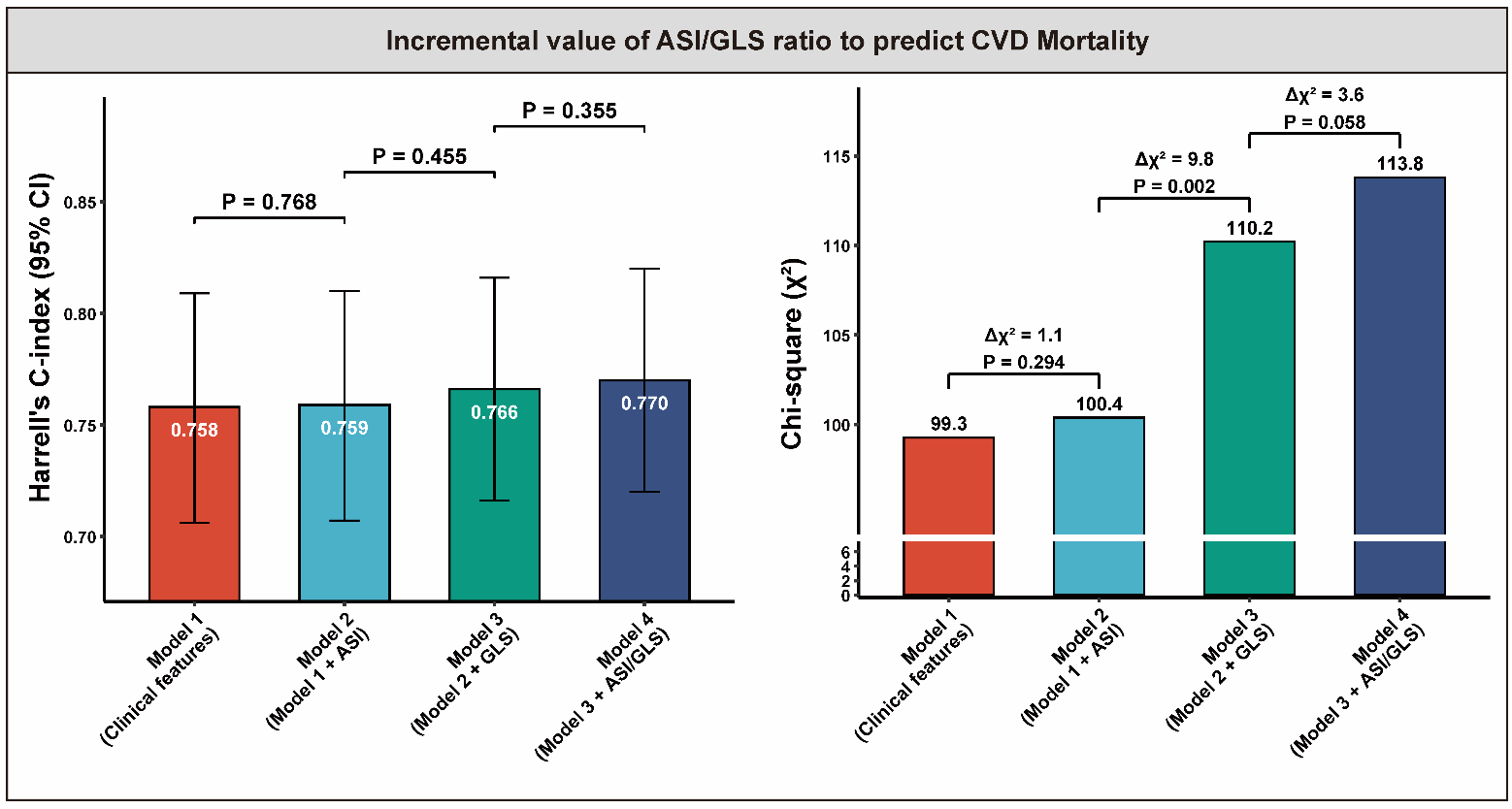


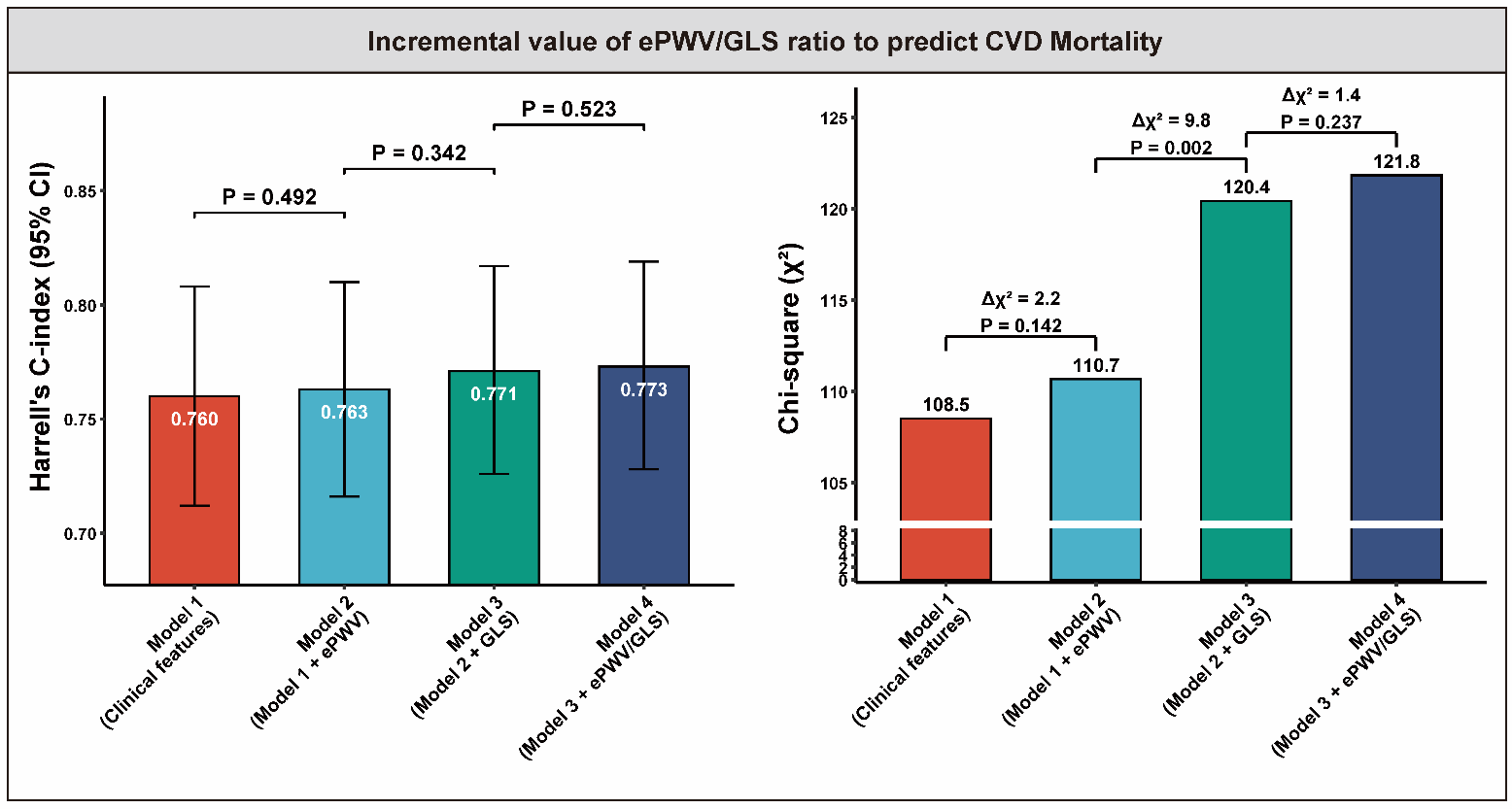


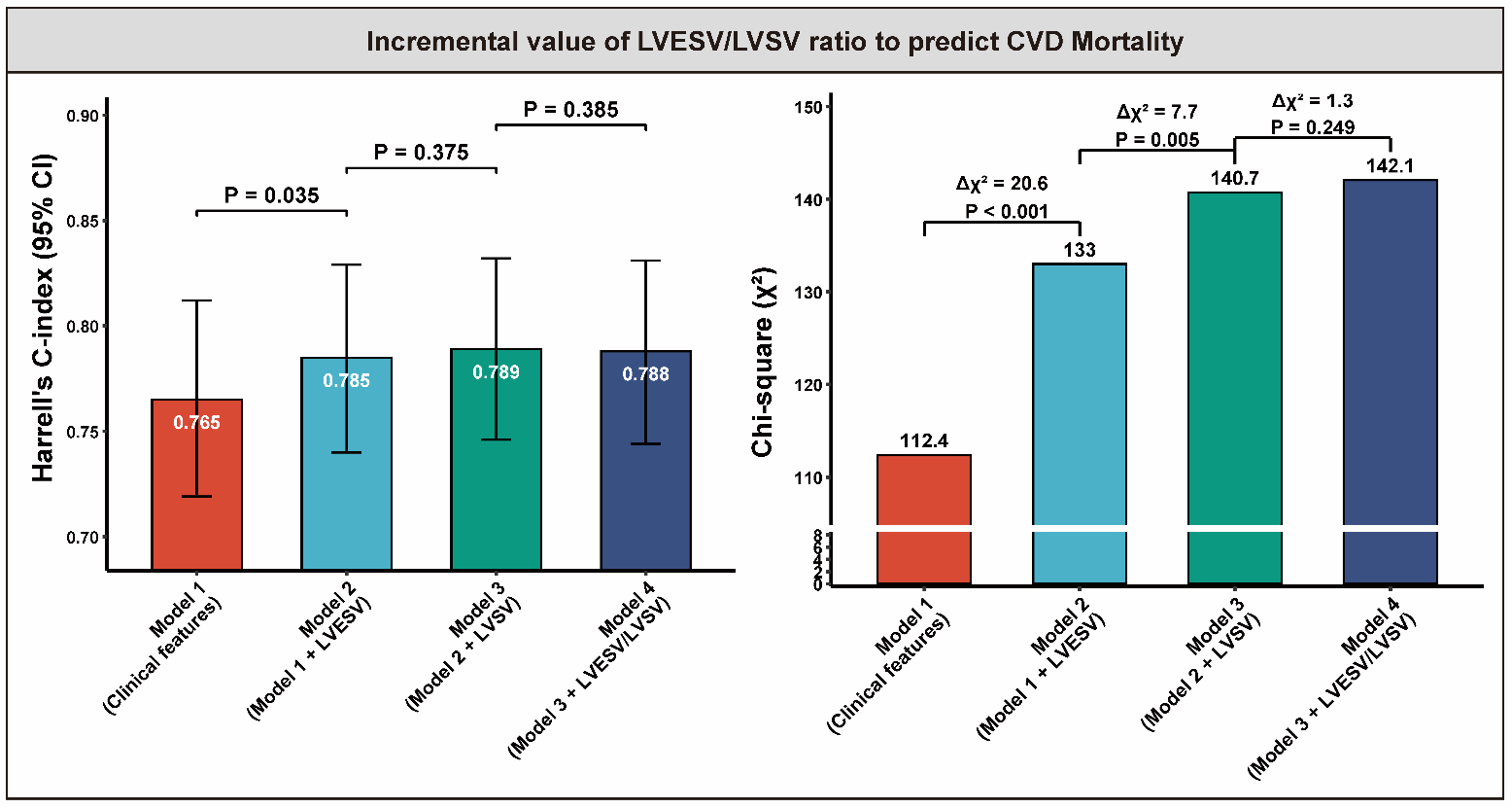


Incremental predictive value of ASI/GLS, ePWV/GLS, and LVESV/LVSV for predicting CVD mortality. The left panels display Harrell's C-indices, and the right panels display the likelihood ratio test chi-square (χ²) statistics. Clinical features included age, sex, race, education, body mass index, smoking status, alcohol intake frequency, healthy physical activity, family history of heart disease, prevalent hypertension, dyslipidemia, diabetes, and coronary heart disease.

Model 1 included clinical features only. Model 2 added the numerator component (ASI, ePWV, or LVESV) to Model 1. Model 3 added the denominator component (GLS or LVSV) to Model 2 (representing the model with both constituent components). Model 4 added the VAC parameter (ratio) to Model 3. *P*-values above brackets indicate the statistical significance of the comparison between the indicated models. Δχ² indicates the change in chi-square statistic.

Abbreviations: ASI/GLS, ratio of arterial stiffness index to global longitudinal strain; CVD, cardiovascular disease; ePWV/GLS, ratio of estimated pulse wave velocity to global longitudinal strain; LVESV/LVSV, ratio of left ventricular end-systolic volume to stroke volume; VAC, ventricular-arterial coupling.

## Figure S19. Subgroup analysis of the multivariable association between VAC parameters and adverse outcomes.


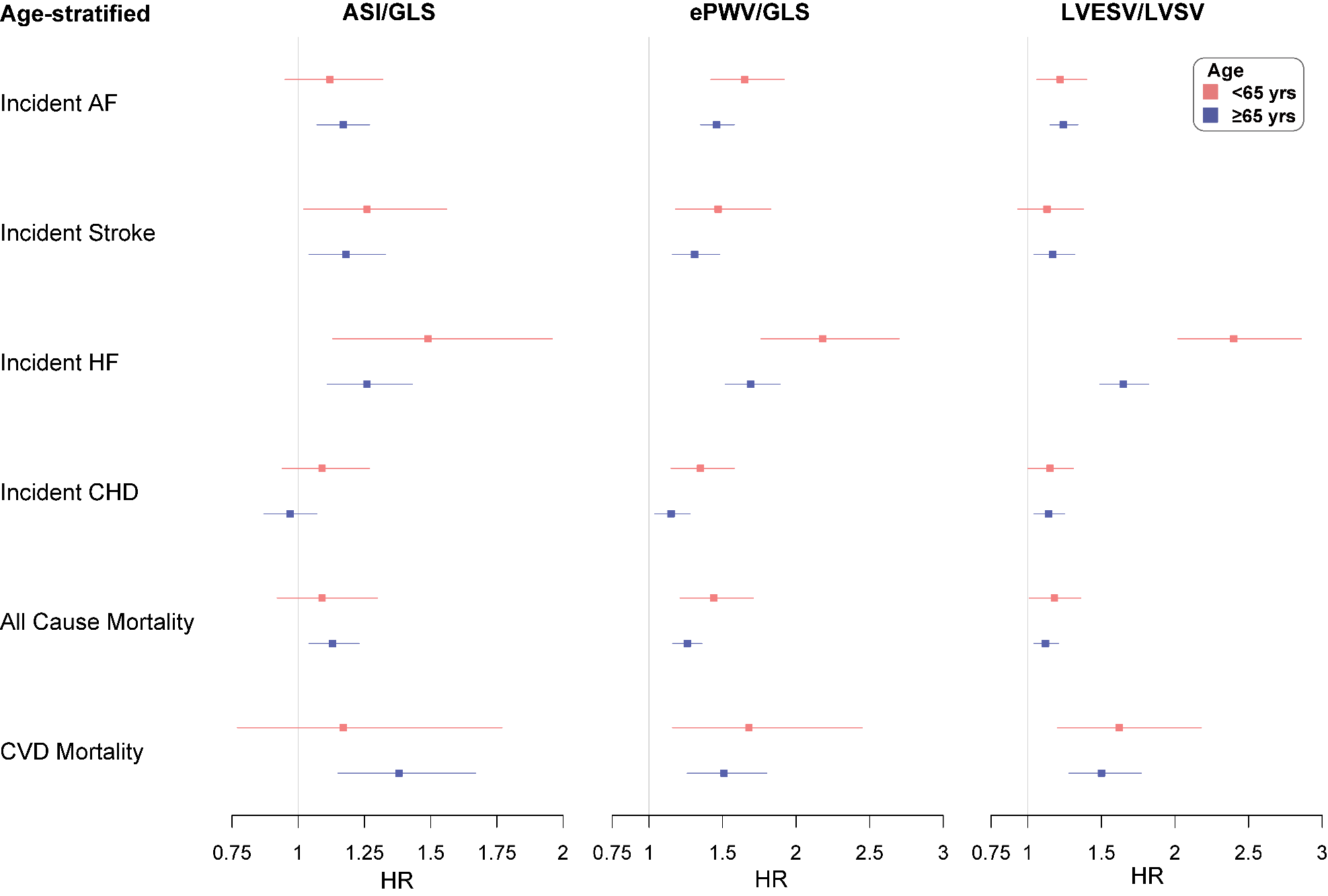


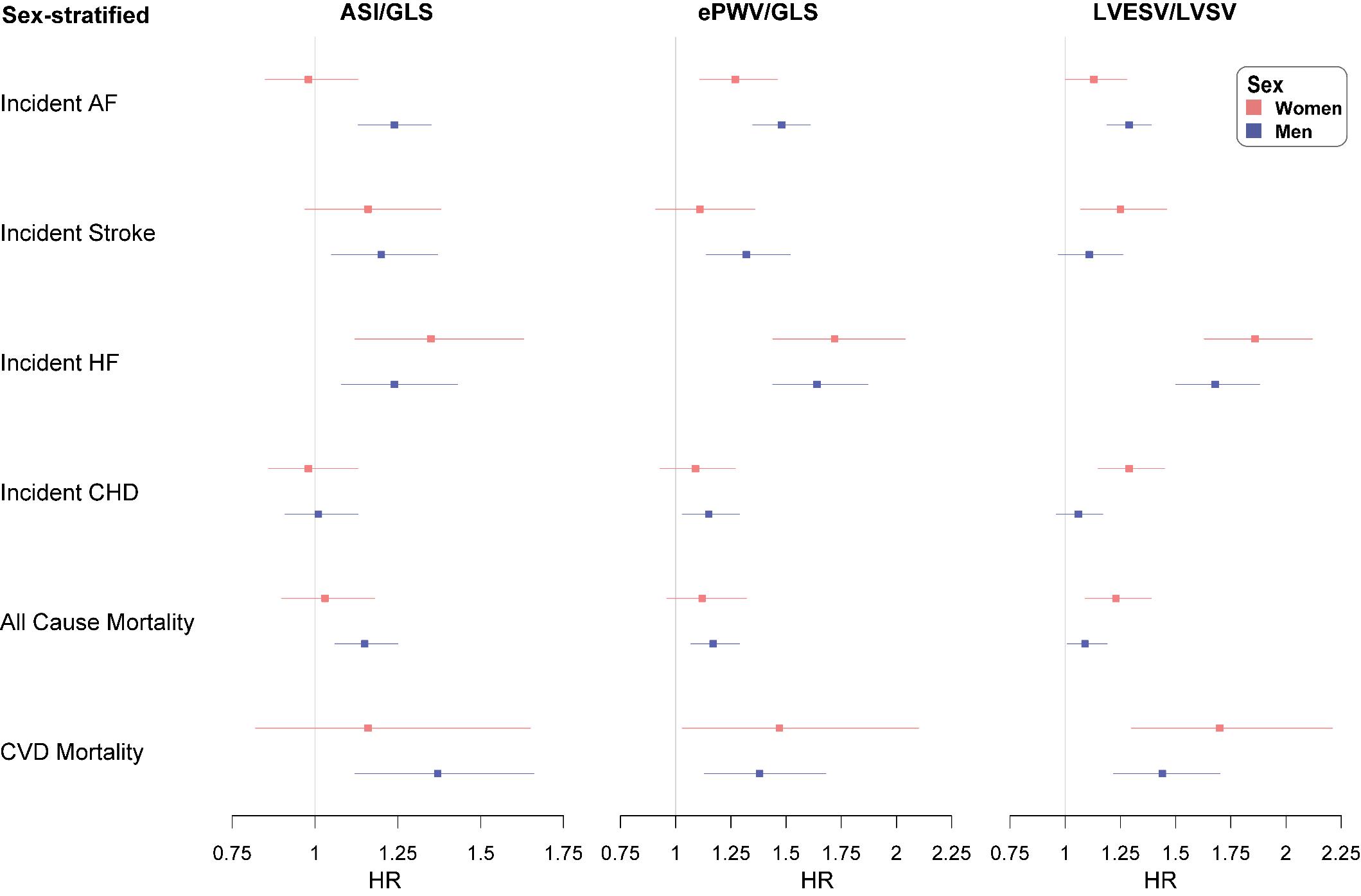


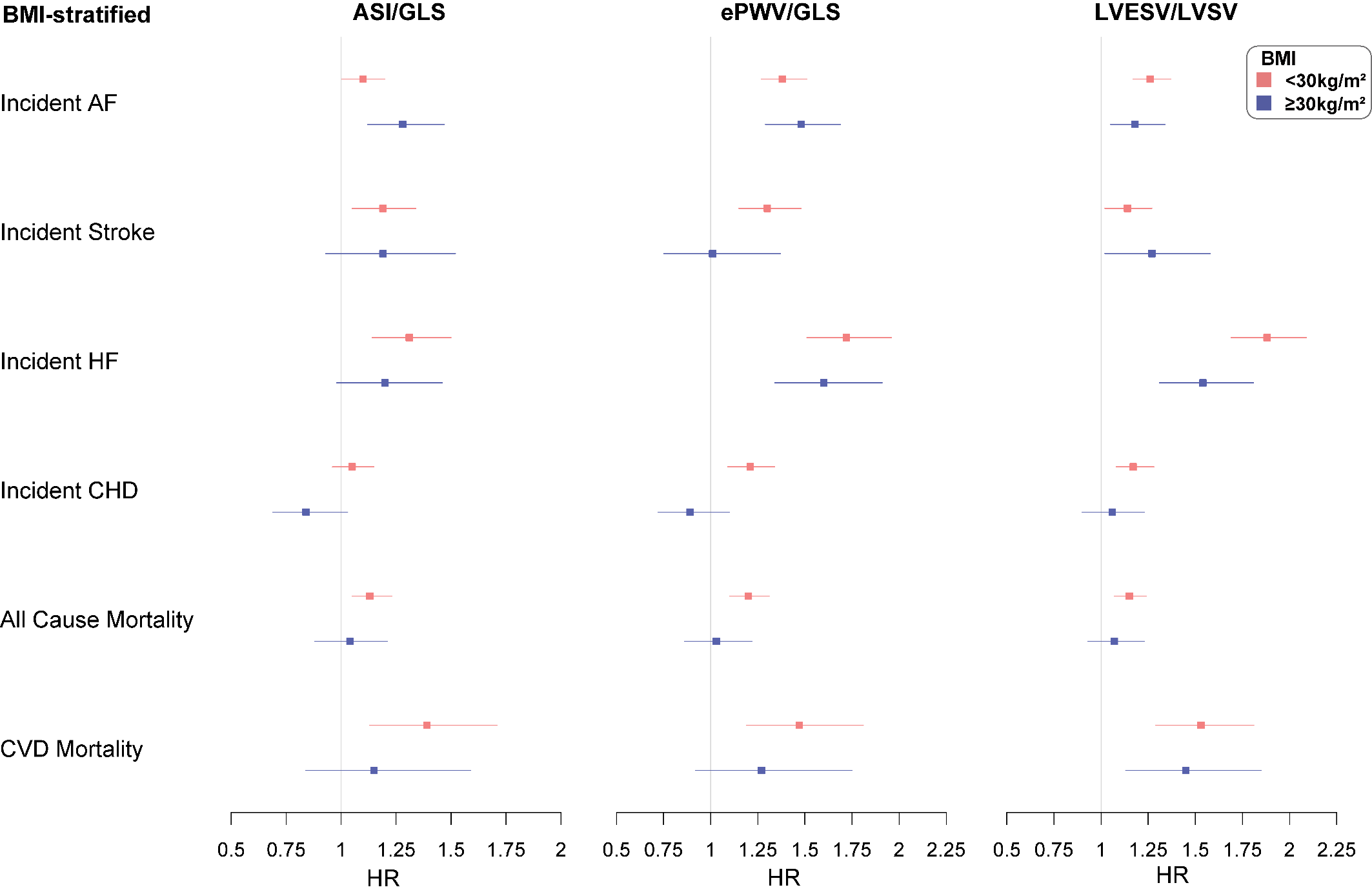


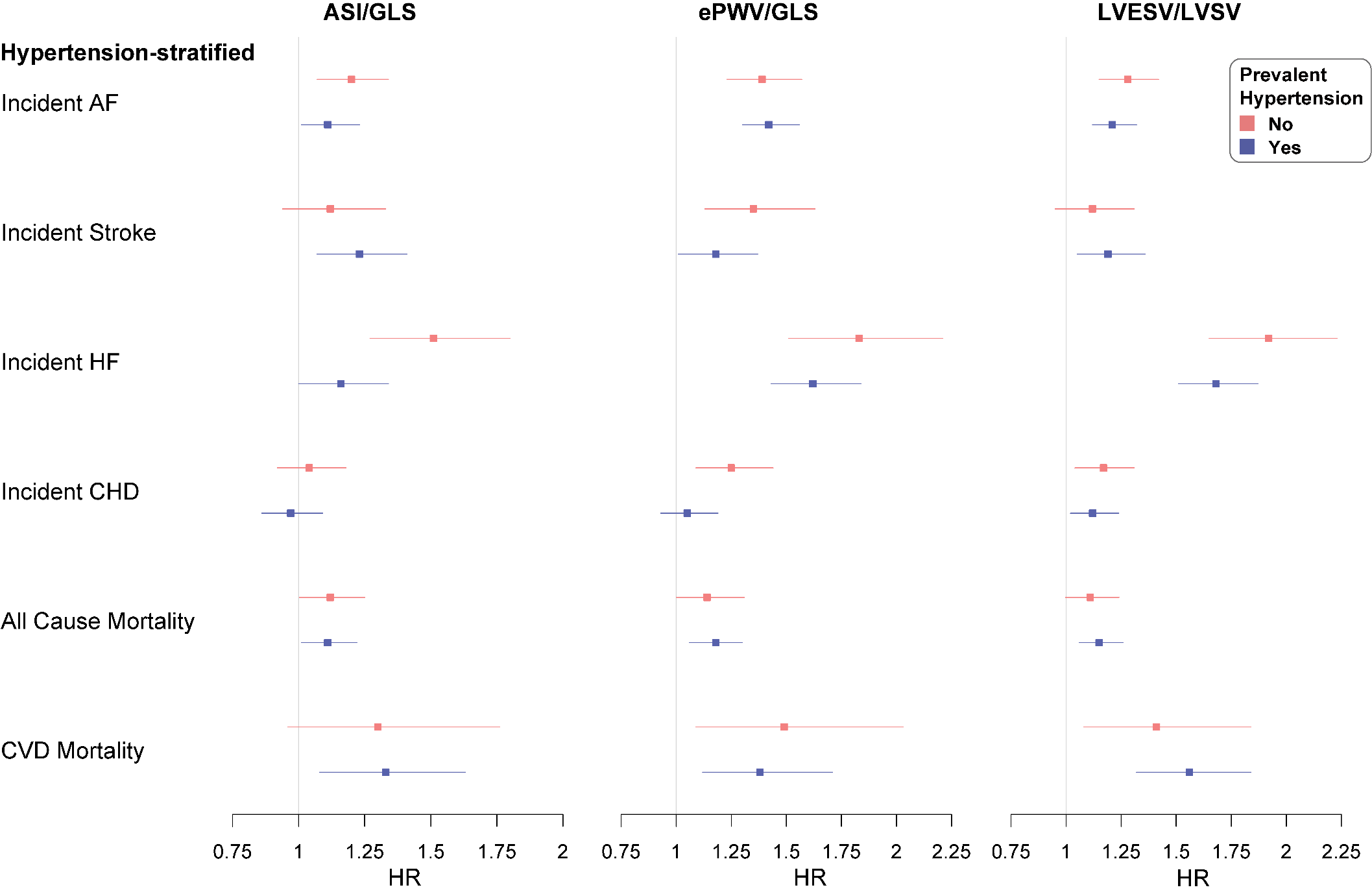


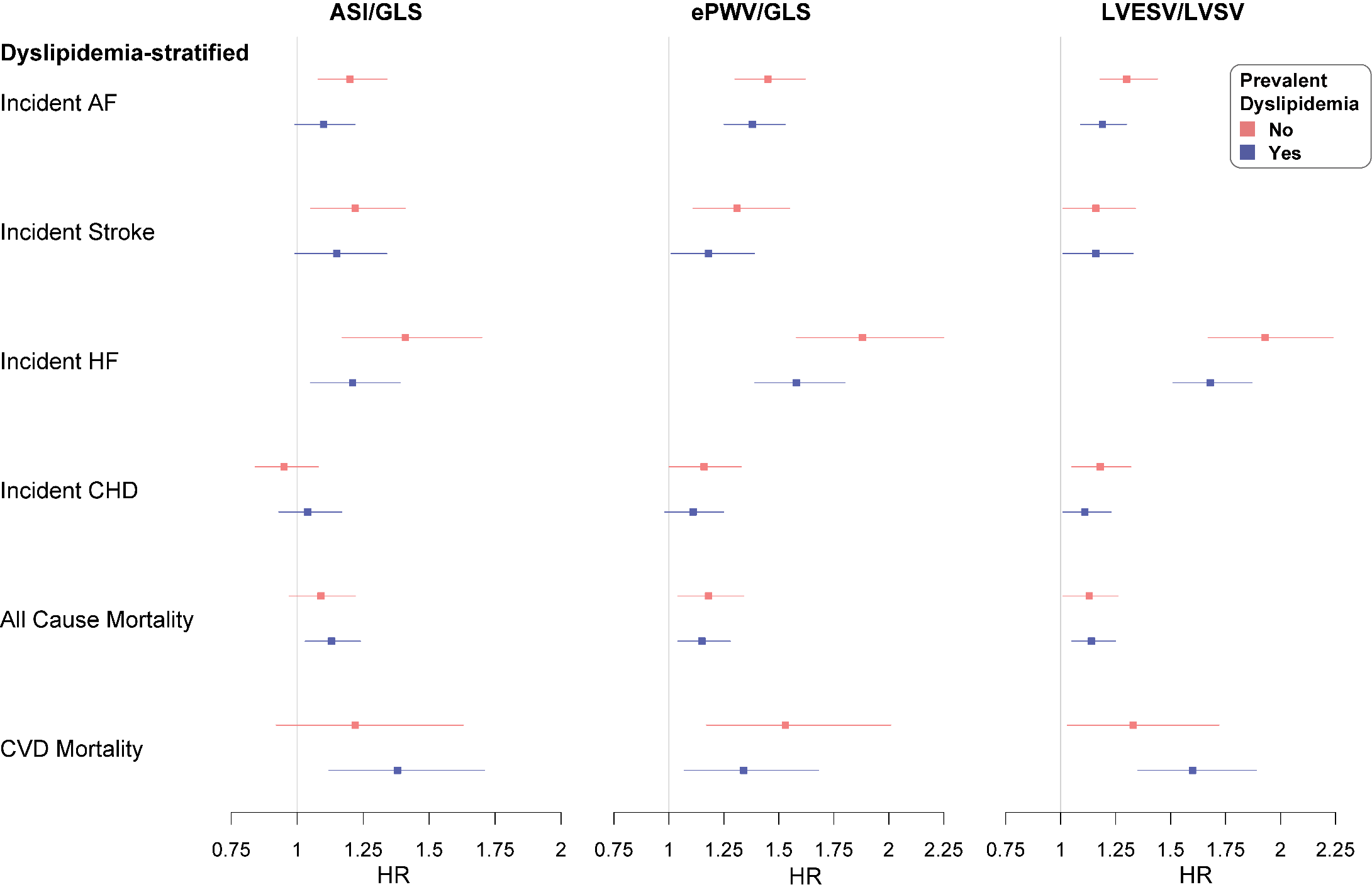


The forest plots show the adjusted HRs and corresponding 95% confidence intervals. Models were adjusted for age, sex, race, education, body mass index, smoking status, alcohol intake frequency, healthy physical activity, family history of heart disease, prevalent hypertension, dyslipidemia, diabetes, and CHD (prevalent CHD was excluded in the incident CHD model). For stratified analyses, the stratifying variable was not included as a covariate. The CVD mortality outcome model was constructed using the Fine-Gray subdistribution hazard model.

Abbreviations: AF, atrial fibrillation; ASI, arterial stiffness index; BMI, body mass index; CHD, coronary heart disease; CI, confidence interval; CVD, cardiovascular disease; ePWV, estimated pulse wave velocity; GLS, left ventricular global longitudinal strain; HF, heart failure; HR, hazard ratio; LVESV, left ventricular end-systolic volume; LVSV, left ventricular stroke volume; VAC, ventricular-arterial coupling.

## Figure S20. Pearson correlation between arterial stiffness, CMR metrics and VAC parameters.


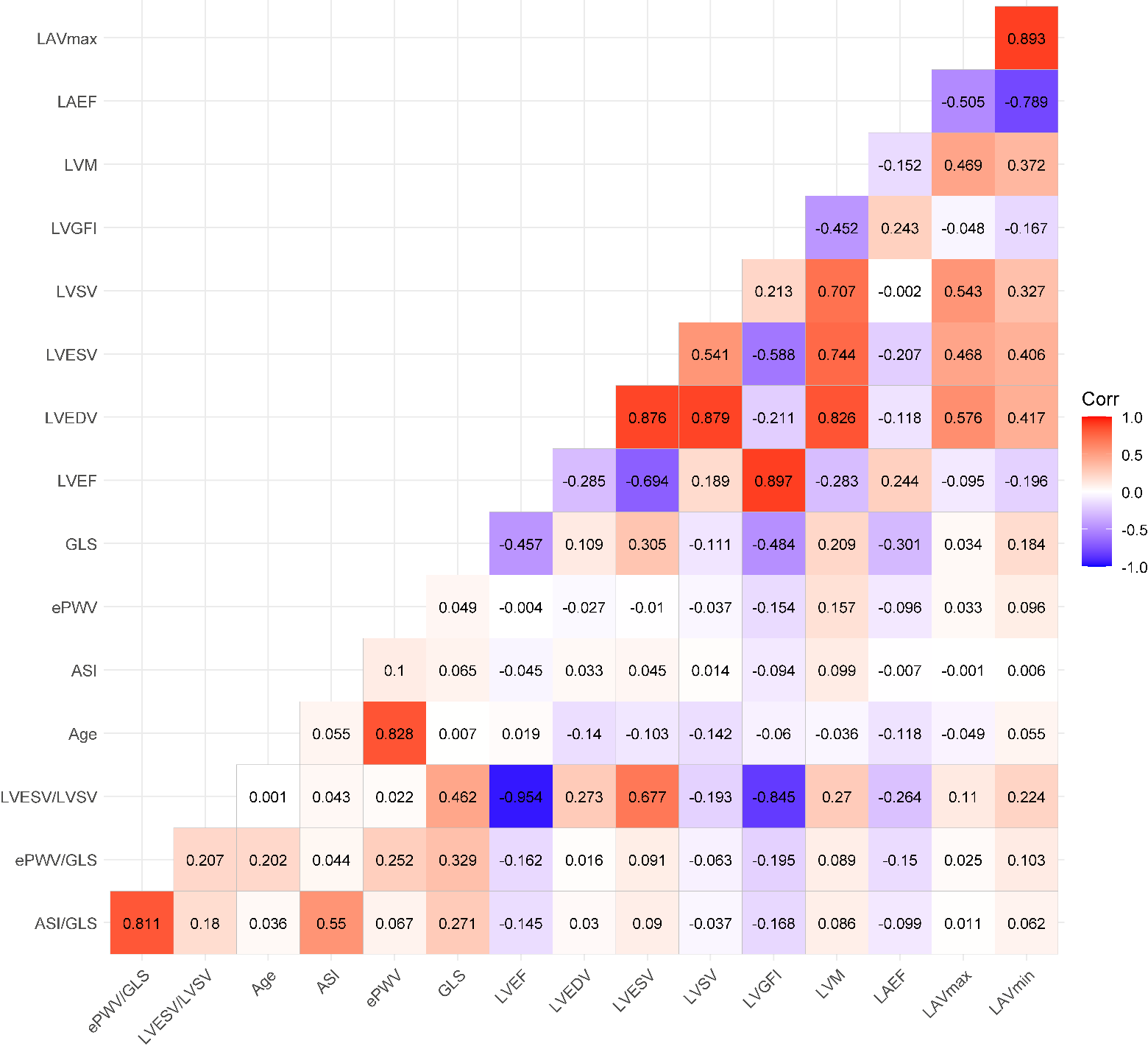


Abbreviations: ASI, arterial stiffness index; ePWV, estimated pulse wave velocity; GLS, left ventricular global longitudinal strain; LAEF, left atrial emptying fraction; LAVmax, maximal left atrial volume; LAVmin, minimal left atrial volume; LVEDV, left ventricular end-diastolic volume; LVEF, left ventricular ejection fraction; LVESV, left ventricular end-systolic volume; LVGFI, left ventricular global function index; LVM, left ventricular mass; LVSV, left ventricular stroke volume; VAC, ventricular-arterial coupling.
